# Supplementary material for: Gold(I) and Silver(I) Complexes Containing Hybrid Sulfonamide/Thiourea Ligands as Potential Leishmanicidal Agents
Source: Pharmaceutics. 2024 Mar 25;16(4):452. doi: 10.3390/pharmaceutics16040452 (PMC11053681; doi:10.3390/pharmaceutics16040452)

*Supplementary Material*

# Gold(I) and Silver(I) Complexes Containing Hybrid Sulfonamide/Thiourea Ligands as Potential Leishmanicidal Agents

**Alice P. Borges [1], Malu M. S. Obata [2], Silvia H. Libardi [3], Rafael O. Trevisan [2], Victor M. Deflon [3], Ulrich Abram [4], Francis B. Ferreira [5], Luiz Antônio S. Costa [6], Antonio O. T. Patrocínio [7], Marcos V. da Silva [2], Júlio C. Borges [3] and Pedro I. S. Maia [1,*]**

[1] Bioactive Compounds Development Research Group, Federal University of Triângulo Mineiro, Av. Dr. Randolfo Borges 1400, Uberaba 38025-440, MG, Brazil; aliceborges98@hotmail.com
[2] Department of Microbiology, Immunology and Parasitology, Federal University of Triângulo Mineiro, Av. Getúlio Guarita, Uberaba 38025-440, MG, Brazil; malu.mateus_21@hotmail.com (M.M.S.O.); rafaelotrevisan@gmail.com (R.O.T.); marcos.silva@uftm.edu.br (M.V.d.S.)
[3] São Carlos Institute of Chemistry, University of São Paulo, Av. Trabalhador São Carlense, 400, São Carlos 13566-590, SP, Brazil; libardish@gmail.com (S.H.L.); deflon@iqsc.usp.br (V.M.D.); borgesjc@iqsc.usp.br (J.C.B.)
[4] Institute of Chemistry and Biochemistry, Freie Universität Berlin, Fabeckstr. 34/36, 14195 Berlin, Germany; ulrich.abram@fu-berlin.de
[5] Associated Faculties of Uberaba, Av. do Tutuna 720, Uberaba 38061-500, MG, Brazil; francisbferreira@gmail.com
[6] Computational Chemistry Research Group, Institute of Exact Sciences, Federal University of Juiz de Fora, Campus Universitário s/n Martelos, Juiz de Fora 36036-900, MG, Brazil; luiz.costa@ufjf.br
[7] Laboratory of Photochemistry and Material Science, Institute of Chemistry, Federal University of Uberlândia, Av. João Naves de Ávila 2121, Uberlândia 38400-902, MG, Brazil; otaviopatrocinio@ufu.br
* Correspondence: pedro.maia@uftm.edu.br

# Table of Contents

**Part 1. IR spectroscopy .** 1

**Figure S1.1:** IR (ATR) spectrum of HL**3**$^{FPh}$;

**Figure S1.1:** IR (ATR) spectrum of HL**4**$^{ClPh}$;

**Figure S1.2:** IR (ATR) spectrum of HL**5**$^{NO2Ph}$;

**Figure S1.3:** IR (ATR) spectrum of HL**6**$^{Al}$;

**Figure S1.5:** IR (ATR) spectrum of $[Ag(HL3^{FPh})_2]NO_3$ (**Ag3**);

**Figure S1.6:** IR (ATR) spectrum of $[Ag(HL4^{ClPh})_2]NO_3$ (**Ag4**);

**Figure S1.7:** IR (ATR) spectrum of $[Ag(HL5^{NO2Ph})_2]NO_3$ (**Ag5**);

**Figure S1.8:** IR (ATR) spectrum of $[Ag(HL6^{Al})_2]NO_3$ (**Ag6**);

**Figure S1.9:** IR (ATR) spectrum of $[Au(HL1^{Ch})_2]Cl$ (**Au1**);

**Figure S1.10:** IR (ATR) spectrum of $[Au(HL2^{Ph})_2]Cl$ (**Au2**);

**Figure S1.11:** IR (ATR) spectrum of $[Au(HL3^{FPh})_2]Cl$ (**Au3**);

**Figure S1.12:** IR (ATR) spectrum of $[Au(HL4^{ClPh})_2]Cl$ (**Au4**);

**Figure S1.13:** IR (ATR) spectrum of $[Au(HL5^{NO2Ph})_2]Cl$ (**Au5**);

**Figure S1.14:** IR (ATR) spectrum of $[Au(HL6^{Al})_2]Cl$ (**Au6**);

**Table S1:** Selected absorption bands in the infrared region for free ligands and complexes;

**Part 2. NMR spectroscopy .** 9

**Figure S2.1:** $^{1}$H NMR spectrum of HL**3**$^{FPh}$ in DMSO-*d*$_6$;

**Figure S2.2:** $^{1}$H NMR spectrum of HL**4**$^{ClPh}$ in DMSO-*d*$_6$;

**Figure S2.3:** $^{1}$H NMR spectrum of HL**5**$^{NO2Ph}$ in DMSO-*d*$_6$;

**Figure S2.4:** $^{1}$H NMR spectrum of HL**6**$^{Al}$ in DMSO-*d*$_6$;

**Figure S2.5:** $^{19}$F NMR spectrum (377 MHz) of H**L3**$^{FPh}$ in DMSO-*d*$_6$;

**Figure S2.6:** $^{1}$H NMR spectrum of $[Ag(HL3^{FPh})_2]NO_3$ (**Ag3**) in DMSO-*d*$_6$;

**Figure S2.7:** $^{1}$H NMR spectrum of $[Ag(HL4^{ClPh})_2]NO_3$ (**Ag4**) in DMSO-*d*$_6$;

**Figure S2.8:** $^{1}$H NMR spectrum of $[Ag(HL5^{NO2Ph})_2]NO_3$ (**Ag5**) in DMSO-*d*$_6$;

**Figure S2.9:** $^{1}$H NMR spectrum of $[Ag(HL6^{Al})_2]NO_3$ (**Ag6**) in DMSO-*d*$_6$;

**Figure S2.10:** $^{1}$H NMR spectrum of $[Au(HL1^{Ch})_2]Cl$ (**Au1**) in DMSO-*d*$_6$;

**Figure S2.11:** $^{1}$H NMR spectrum of $[Au(HL2^{Ph})_2]Cl$ (**Au2**) in DMSO-*d*$_6$;

**Figure S2.12:** $^{1}$H NMR spectrum of $[Au(HL3^{FPh})_2]Cl$ (**Au3**) in DMSO-*d*$_6$;

**Figure S2.13:** $^{1}$H NMR spectrum of $[Au(HL4^{ClPh})_2]Cl$ (**Au4**) in DMSO-*d*$_6$

**Figure S2.14:** $^{1}$H NMR spectrum of $[Au(HL5^{NO2Ph})_2]Cl$(**Au5**) in DMSO-*d*$_6$;

**Figure S2.15:** $^{1}$H NMR spectrum of $[Au(HL6^{Al})_2]Cl$ (**Au6**) in DMSO-*d*$_6$;

**Figure S2.16:** $^{19}$F NMR spectrum (377 MHz) of $[Ag(HL3^{FPh})_2]NO_3$ (**Ag3**) in DMSO-*d*$_6$;

**Figure S2.17:** $^{19}$F NMR spectrum (377 MHz) of $[Au(HL3^{FPh})_2]Cl$ (**Au3**) in DMSO-*d*$_6$;

**Part 3. ESI+ MS spectra .** 18

**Figure S3.1:** ESI(+) MS spectrum of $[Ag(HL3^{FPh})_2]NO_3$ (**Ag3**);

**Figure S3.2:** ESI(+) MS spectrum of $[Au(HL1^{Ch})_2]Cl$ (**Au1**);

**Figure S3.4:** ESI(+) MS spectrum of $[Au(HL2^{Ph})_2]Cl$ (**Au2**);

**Figure S3.5:** ESI(+) MS spectrum of $[Au(HL3^{FPh})_2]Cl$ (**Au3**);

**Figure S3.6:** ESI(+) MS spectrum of $[Au(HL4^{ClPh})_2]Cl$ (**Au4**);

**Figure S3.7:** ESI(+) MS spectrum of $[Au(HL6^{Al})_2]Cl$ (**Au6**);

**Part 4. Crystallographic data.** 24

**Figure S4.1:** Hydrogen bonds involved in the crystalline structure of the **HL3**$^{FPh}$ ligand. Intermolecular hydrogen bonds: [N(1)···O(1) = 2.910(3) Å, N(1)-H(1)···O(1)= 115.4°], [N(2)···S(1) = 3.311(2) Å, N(2)-H(2)···S(1) = 160.8°], [N(1)···O(2) = 3.420(3) Å, N(1)-H(1)···O(2) =

98.1°], [N(3)···O(2) = 2.959(2) Å, N(3)-H(3)···O(2) = 147.7°]. Intramolecular hydrogen bonding: [N(3)···N(1) = 2.659(3) Å, N(3)-H(3)···N(1)= 110.3 °]. Used symmetry operations (′) x-1, y, z, (′′) -x, -y+1, -z+1 and (′′′) -x, -y+2, -z+1. Red dotted lines (between donor and acceptor atoms) represent hydrogen bonds;

**Figure S4.2:** Hydrogen bonds involved in the crystalline structure of the **HL5$^{NO2Ph}$** ligand. Intermolecular hydrogen bonds: [N(1)···O(1′) = 2.887(4) Å, N(1)-H(1)···O(1′)= 117.4°], [N(2)···S(1′′) = 3.347(3) Å, N(2)-H(2)···S(1′′) = 165.2°], [N(3) ···O(2′′′) = 3.040(4) Å, N(3)-H(3)···O(2′′′) = 151.8 °]. Used symmetry operations (′) x-1, y, z, (′′) -x, -y+1, -z+1 and (′′′) -x, -y, -z+1. Blue dotted lines (between donor and acceptor atoms) represent;

**Figure S4.8:** Hydrogen bonds involved in the crystalline structure of the **HL6$^{Al}$** ligand. Intermolecular hydrogen bonds: [N(3)···O(2) = 3.024(3) Å, N(3)-H(3)···O(2)= 129.0°], [N(2)···S(1) = 3.3475(18) Å, N(2)-H(2)···S(1) = 159.0 °], [N(1)···S(1) = 3.356(2) Å, N(1)-H(1)···S(1)= 118.4 °]. Used symmetry operations (′) -x, -y, -z+1, (′′) -x+1, -y+1, -z+1 and (′′′) x-1, y, z. Blue dotted lines (between donor and acceptor atoms) represent hydrogen bonds;

**Figure S4.4** Ellipsoid representation of [Au(HL1$^{Ch}$)$_2$]Cl·$CH_3OH$ (**Au1**·$CH_3OH$). The hydrogen atoms have been omitted for clarity. A solvent mask was calculated and 108 electrons were found in a volume of 518 Å$^3$ in 1 void per unit cell. This is consistent with the presence of 0.5[CH2Cl2;2H2O] per Asymmetric Unit which account for 120 electrons per unit cell.

**Figure S4.5** Ellipsoid representation of [Au(HL2$^{Ph}$)$_2$]Cl·$CH_3OH$ (**Au2**·$CH_3OH$). The hydrogen atoms have been omitted for clarity.

**Figure S4.6:** Hydrogen bonds involved in the crystalline structure of the **Au2** complex. Intermolecular hydrogen bonds: [N(1)···Cl(1) = 3.173(4) Å, N(1)-H(1)···Cl(1)= 113.8 °], [O(1S)···Cl(1) = 3.135(5) Å, O(1S)-H(1S)···Cl(1) = 152.4°], [N(3A)···O(1) = 2.775(14) Å, N(3A)-H(3A)···O(1) = 146.1 °], [N(3B)···O(1) = 3.149(14) Å, N(3B)-H(3B)···O(1) = 136.9 °]. Intramolecular hydrogen bonding: [N(2)···O(1S) = 2.905(5) Å, N(2)-H(2)···O(1S)= 135.6 °]. Used symmetry operations (′) -x, +y, -z. Blue dotted lines (between donor and acceptor atoms) represent hydrogen bonds;

**Part 5. Photophysical studies** 27

**Figure S5.1:** Electronic spectra of **Ag3** (green line), **Ag4** (orange line), **Ag5** (red line) and **Ag6** (purple line) in the 210 to 350 nm range ($10^{-6}$ M $CH_3CN$ solution);

**Figure S5.2:** Electronic spectra of **Au1** (blue line), **Au2** (pink line), **Au3** (green line), **Au4** (orange line), **Au5** (red line) and **Au6** (purple line) in the 210 to 350 nm range ($10^{-6}$ M $CH_3OH$ solution for **Au1**, **Au2**, **Au3**, **Au6** and $10^{-6}$ M solution $CH_3CN$ for **Au4** and **Au5**);

**Figure S5.9:** Linear regression for **Ag3** (green line), **Ag4** (orange line), **Ag5** (red line) and **Ag6** (purple line);

**Table S2:** Electronic spectra data obtained for the **Ag3** and **Ag4** complexes;

**Table S3:** Electronic spectra data obtained for the **Ag5** and **Ag6** complexes;

**Figure S5.10:** Linear regression for **Au1** (blue line), **Au2** (pink line), **Au3** (green line), **Au4** (orange line), **Au5** (red line) and **Au6** (purple line);

**Table S4:** Electronic spectra data obtained for the **Au1** and **Au2** complexes;

**Table S5:** Electronic spectra data obtained for the **Au3** and **Au4** complexes;

**Table S6:** Electronic spectra data obtained for the **Au5** and **Au6** complexes;

**Figure S5.5:** (a) Emission spectra of **Au1** (blue line) and **Au2** (pink line) in $CH_3OH$ at 298 K; λexc = 255 nm for **Au1** and 270 nm for **Au2**; (b) Emission spectra of $CH_3OH$ at 298 K; λexc = 250 nm;

**Figure S5.6:** Emission spectra of **Au1** (blue dashed line) and **Au2** (pink dashed line) in MeOH at 77 K; λexc = 255 nm for **Au1** and 270 nm for **Au2**;

**Part 6. DFT studies** 32

**Table S7. Basis set functions scheme used for the gold complexes.**

**Table S8:** Vibrational modes and their theoretical assignments related to the C=S bond;
**Figure S6.1:** Optimized structures of the silver complexes;
**Table S9:** Main theoretical structural data of the silver complexes;
**Figure S6.2:** Optimized structures of the gold complexes;
**Table S10:** Main theoretical structural data of the gold complexes;
**Figure S6.3:** HOMO and LUMO orbitals in the silver complexes;
**Figure S6.4 -** HOMO and LUMO orbitals in the gold complexes;
**Figure S6.5:** Optimized structure of $[Au(HL3^{FPh})_2]^+$ (**Au3**) with the charges obtained for the AIM data;
**Table S11:** Electronic properties of the gold(I) complexes. The values are shown, respectively, for each bond in the order they appear;

**Part 7. Biological studies** 41

**Figure S7.1:** Graph of the viability versus concentrations (µM) of the compounds;
**Figure S7.2:** Leishmanicidal activity against to the *Leishmania infantum* parasite by concentrations of the compound. Blue curve represents the reference leishmanicidal drug, Glucantime. Black curve represents each compound;
**Figure S7.3:** Leishmanicidal activity against to the *Leishmania braziliensis* parasite by concentrations of the compound. Blue curve represents the reference leishmanicidal drug, Glucantime. Black curve represents each compound;
**Figure S7.4:** (a) percentage of the number of infected in 200 macrophages by *Leishmania infantum*; (b) percentage of leishmaniasis that infect 100 cells/macrophages; (c) average percentage of leishmaniasis that infect 200 cells/macrophages; of the selected compounds;
**Figure S7.5:** Image captured under a fluorescence microscope (EVOS) of Raw macrophages infected by *Leishmania infantum* after treatment with a Glucantime (leishmanicidal drug) (A) and the selected compounds;
**Figure S7.6:** (A) Fluorescence emission spectra ($\lambda_{ex}$ = 295 nm) of LbOYE protein in the concentration of 1 µmol $L^{-1}$ in the presence of increasing concentrations of the $HL1^{Ch}$ compound. (B) Variation of maximum fluorescence intensities ($\lambda_{max}$ = 333 nm) and non-linear fit to the Hill equation (Equation 1). (C) Logarithmic relationship to obtain the number of bonding sites according to Equation 2. (D) Relationship of temperatures of 25 and 37 °C. The spectra were obtained in Tris-HCl buffer, pH 8.0; 100 mmol $L^{-1}$ of NaCl and 2.5 % of DMSO;
**Figure S7.7:** (A) Fluorescence emission spectra ($\lambda_{ex}$ = 295 nm) of LbOYE protein in the concentration of 1 µmol $L^{-1}$ in the presence of increasing concentrations of the $HL3^{FPh}$ compound. (B) Variation of maximum fluorescence intensities ($\lambda_{max}$ = 333 nm) and non-linear fit to the Hill equation (Equation 1). (C) Logarithmic relationship to obtain the number of bonding sites according to Equation 2. (D) Relationship of temperatures of 25 and 37 °C. The spectra were obtained in Tris-HCl buffer, pH 8.0; 100 mmol $L^{-1}$ of NaCl and 2.5 % of DMSO.
**Figure S7.8.** Fluorescence emission spectra ($\lambda_{ex}$ = 295 nm) of LbOYE protein in the concentration of 1 µmol $L^{-1}$ in the presence of increasing concentrations of the $[Au(HL1^{Ch})_2]Cl$ (**Au1**) complex. (B) Variation of maximum fluorescence intensities ($\lambda_{max}$ = 333 nm) and non-linear fit to the Hill equation (Equation 1). (C) Logarithmic relationship to obtain the number of bonding sites according to Equation 2. (D) Comparison between the temperatures of 25 °C and 37 °C. The spectra were obtained in Tris-HCl buffer, pH 8.0; 100 mmol $L^{-1}$ of NaCl and 2.5 % of DMSO.

**Part 8. Docking interactions** 50

**Figure S8.1:** Representative figure of the $HL1^{Ch}$ ligand at the site of the LbOYE**;**
**Figure S8.2:** Representative figure of the $HL3^{FPh}$ ligand at the site of the LbOYE**;**

**Figure S8.11:** Representative figure of the HL4$^{ClPh}$ ligand at the site of the LbOYE**;**
**Figure S8.12:** Representative figure of the HL5$^{NO2Ph}$ ligand at the site of the LbOYE**;**
**Figure S8.13:** Representative figure of the HL6$^{Al}$ ligand at the site of the LbOYE**;**
**Figure S8.14:** Representative figure of the [Ag(HL1$^{Ch}$)$_2$]$NO_3$ complex at the site of the LbOYE**;**
**Figure S8.15:** Representative figure of the [Ag(HL2$^{Ph}$)$_2$]$NO_3$ complex at the site of LbOYE**;**
**Figure S8.16:** Representative figure of the [Ag(HL3$^{FPh}$)$_2$]$NO_3$ complex at the site of the LbOYE**;**
**Figure S8.17:** Representative figure of the [Ag(HL4$^{ClPh}$)$_2$]$NO_3$ complex at the site of the LbOYE**; Figure S8.18:** Representative figure of the [Ag(HL5$^{NO2Ph}$)$_2$]$NO_3$ complex at the site of the LbOYE**;**
**Figure S8.19:** Representative figure of the [Au(HL1$^{Ch}$)$_2$]Cl complex at the site of the LbOYE;
**Figure S8.20:** Representative figure of the [Au(HL2$^{Ph}$)$_2$]Cl complex at the site of the LbOYE**;**
**Figure S8.21:** Representative figure of the [Au(HL3$^{FPh}$)$_2$]Cl complex at the site of the LbOYE**;**
**Figure S8.22:** Representative figure of the [Au(HL4$^{ClPh}$)$_2$]Cl complex at the site of the LbOYE**;**
**Figure S8.23:** Representative figure of the [Au(HL5$^{NO2Ph}$)2]Cl complex at the site of the LbOYE.

**Figure S1.1** - IR (ATR) spectrum of HL**3**$^{FPh}$.

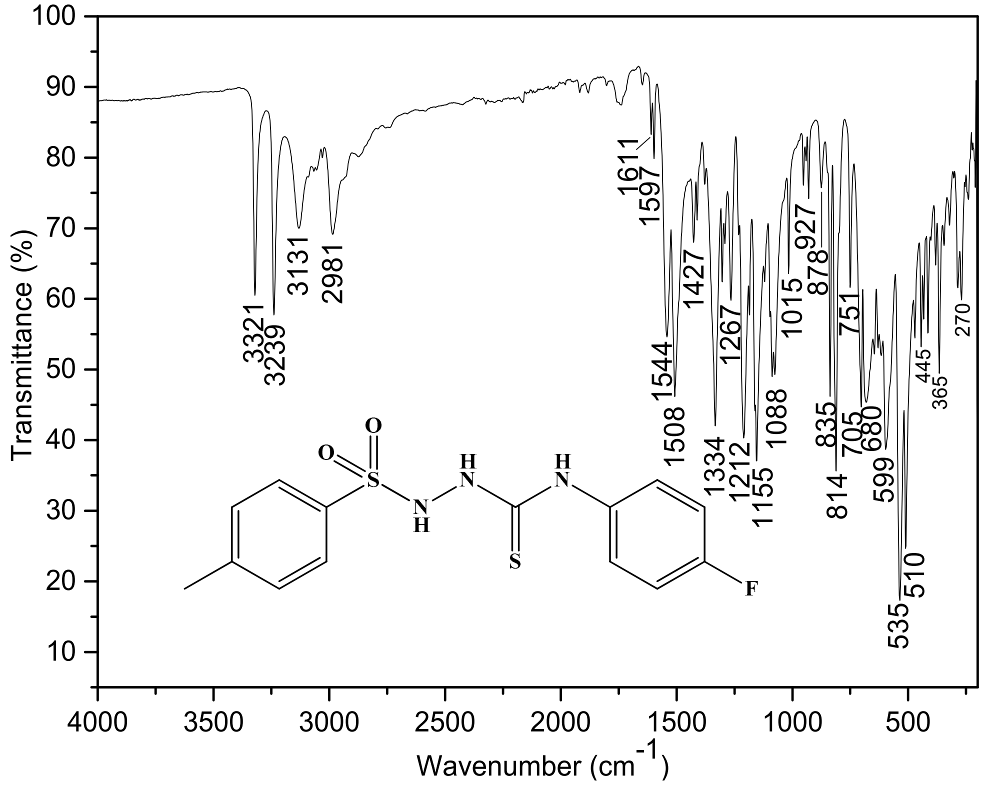


**Figure S1.24** – IR (ATR) spectrum of HL**4**$^{ClPh}$.

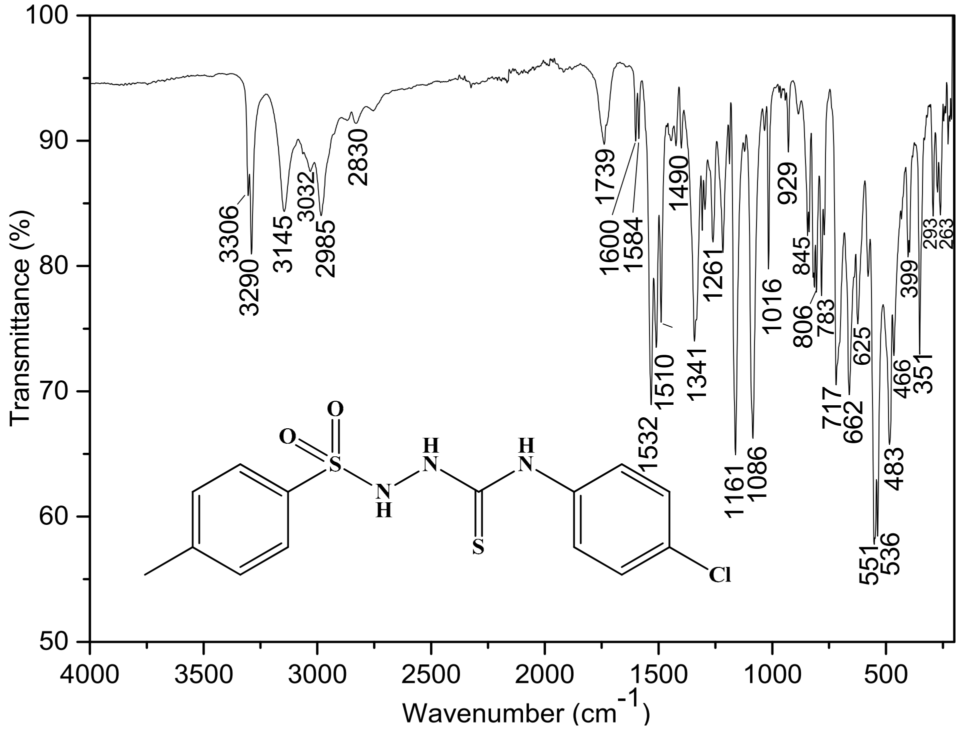

**Figure S1.25 -** IR (ATR) spectrum of HL**5**$^{NO2Ph}$.

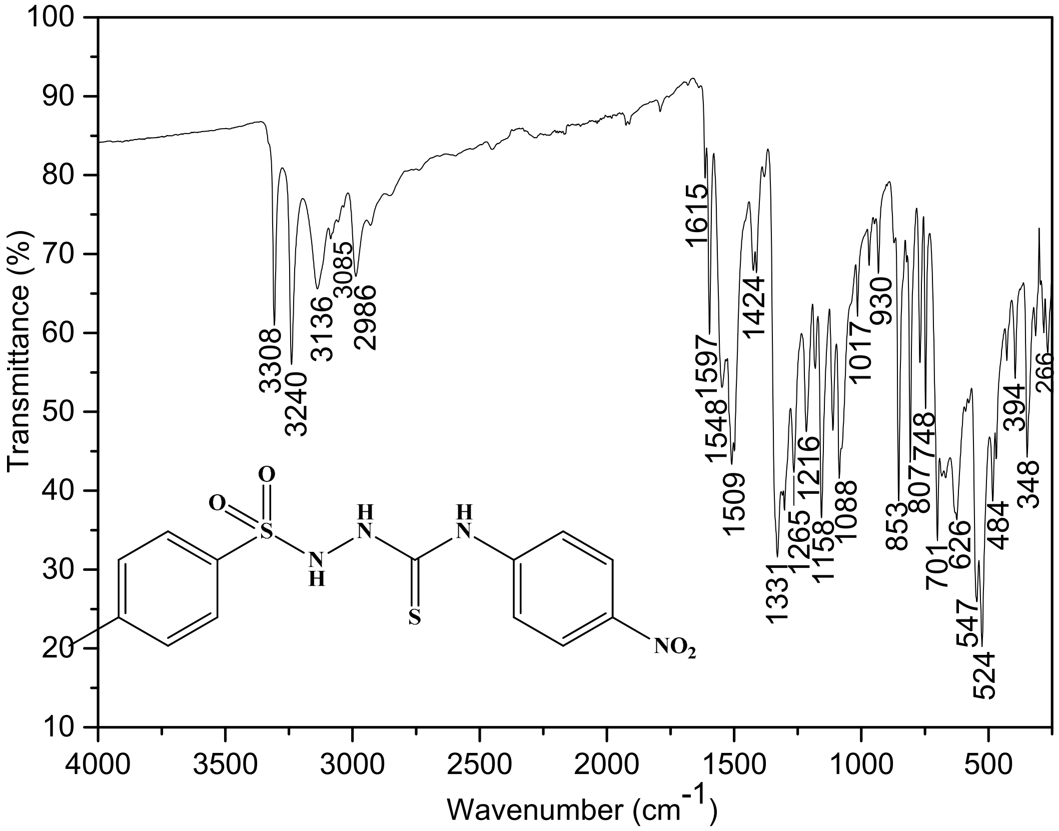


**Figure S1.26 -** IR (ATR) spectrum of HL**6**$^{Al}$.

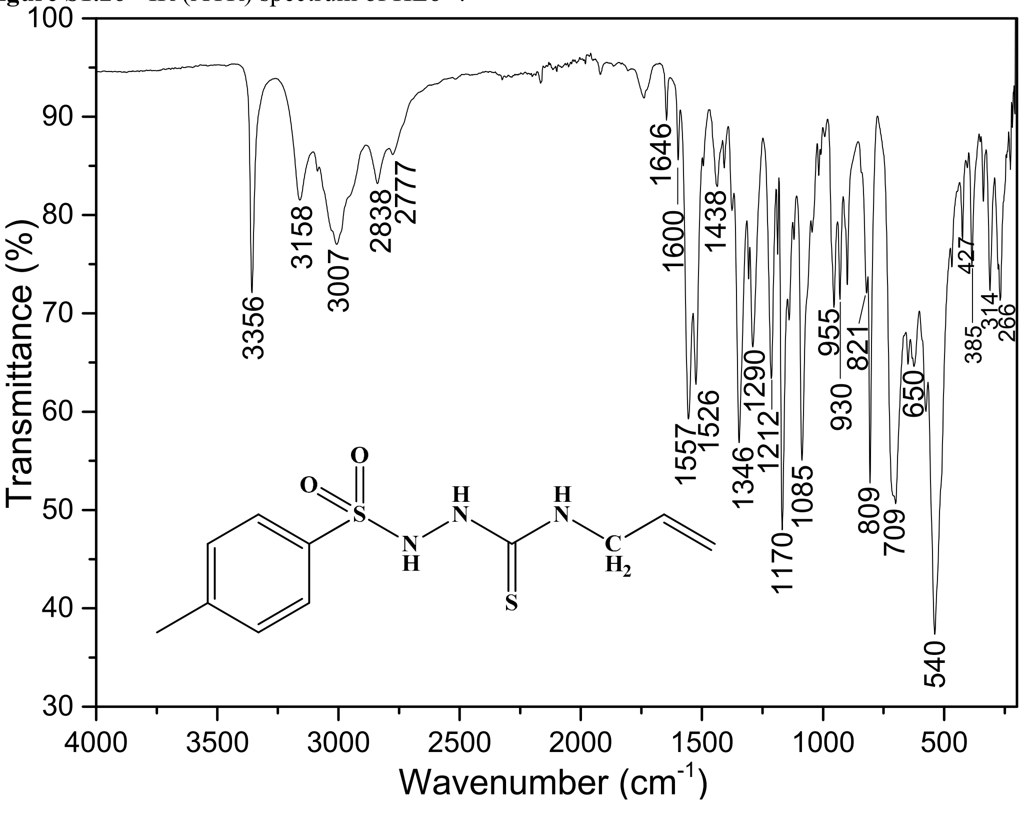

**Figure S1.5 -** IR (ATR) spectrum of $[Ag(HL3^{FPh})_2]NO_3$ (**Ag3**).

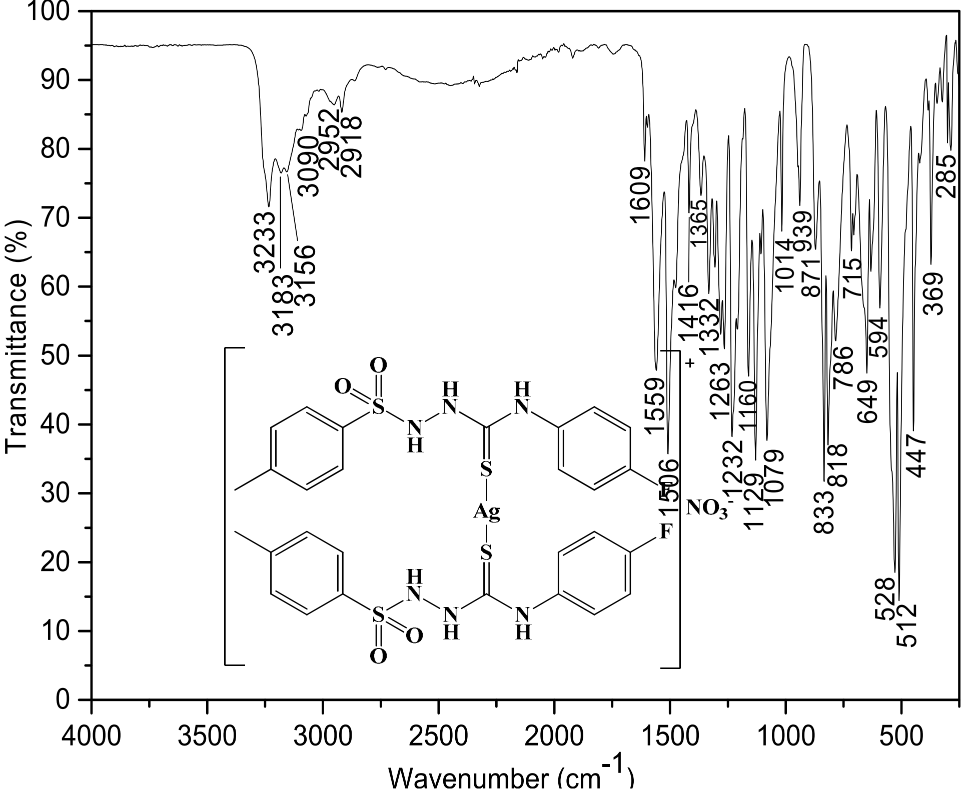


**Figure S1.6 -** IR (ATR) spectrum of $[Ag(HL4^{ClPh})_2]NO_3$ (**Ag4**).

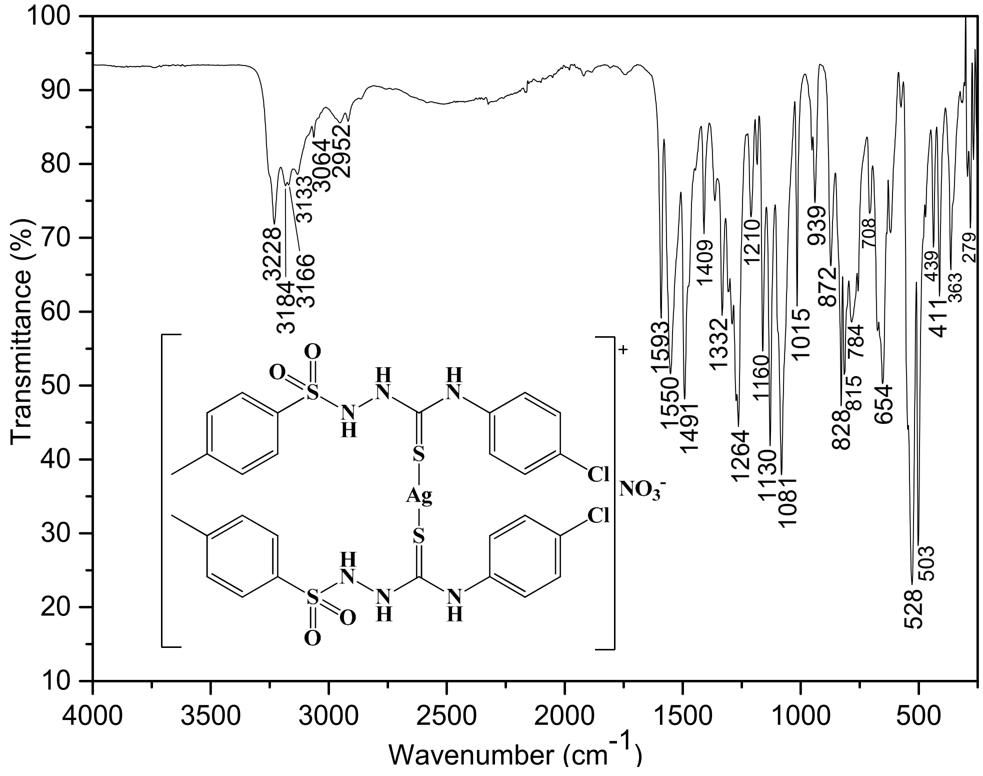


**Figure S1.7 -** IR (ATR) spectrum of $[Ag(HL5^{NO2Ph})_2]NO_3$ (**Ag5**).

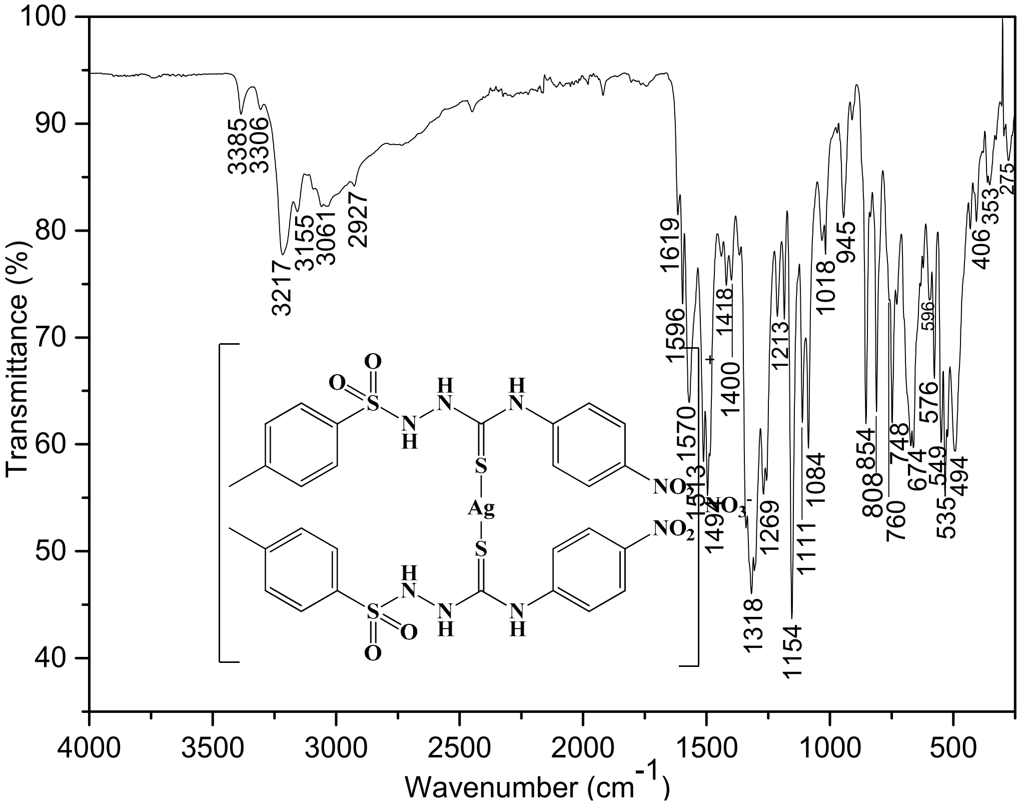


**Figure S1.8 -** IR (ATR) spectrum of $[Ag(HL6^{Al})_2]NO_3$ (**Ag6**).

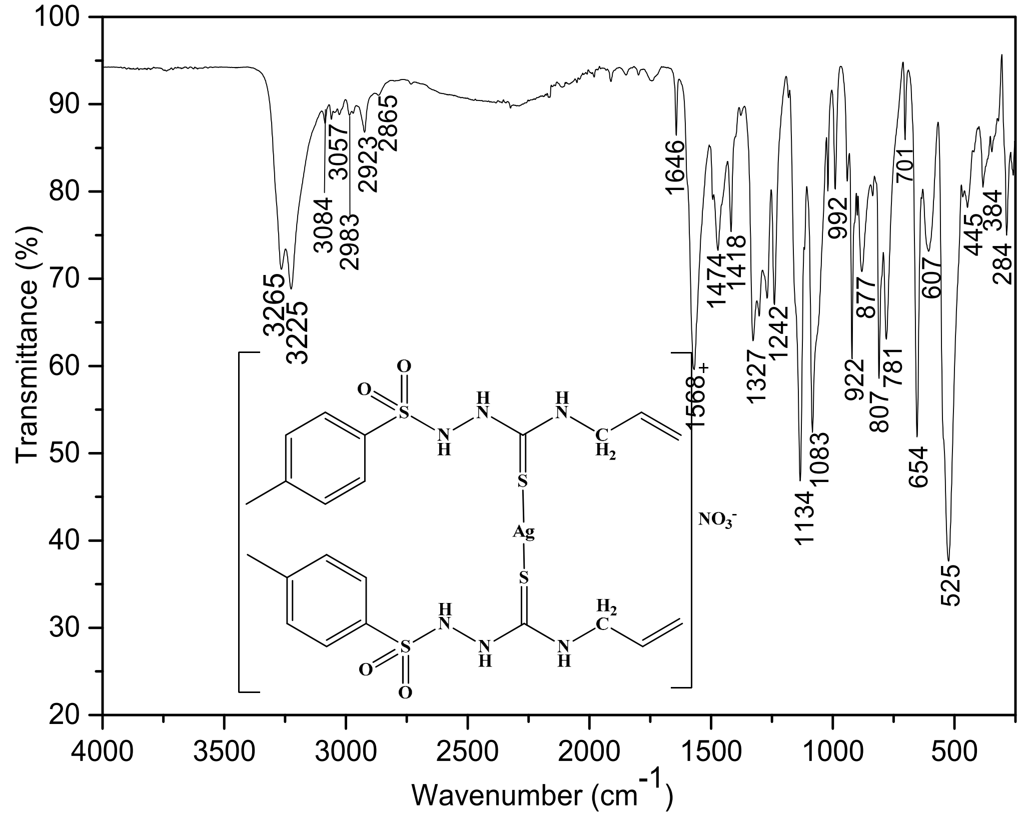

**Figure S1.9** – IR (ATR) spectrum of [Au(HL1$^{Ch}$)$_2$]Cl (**Au1**).

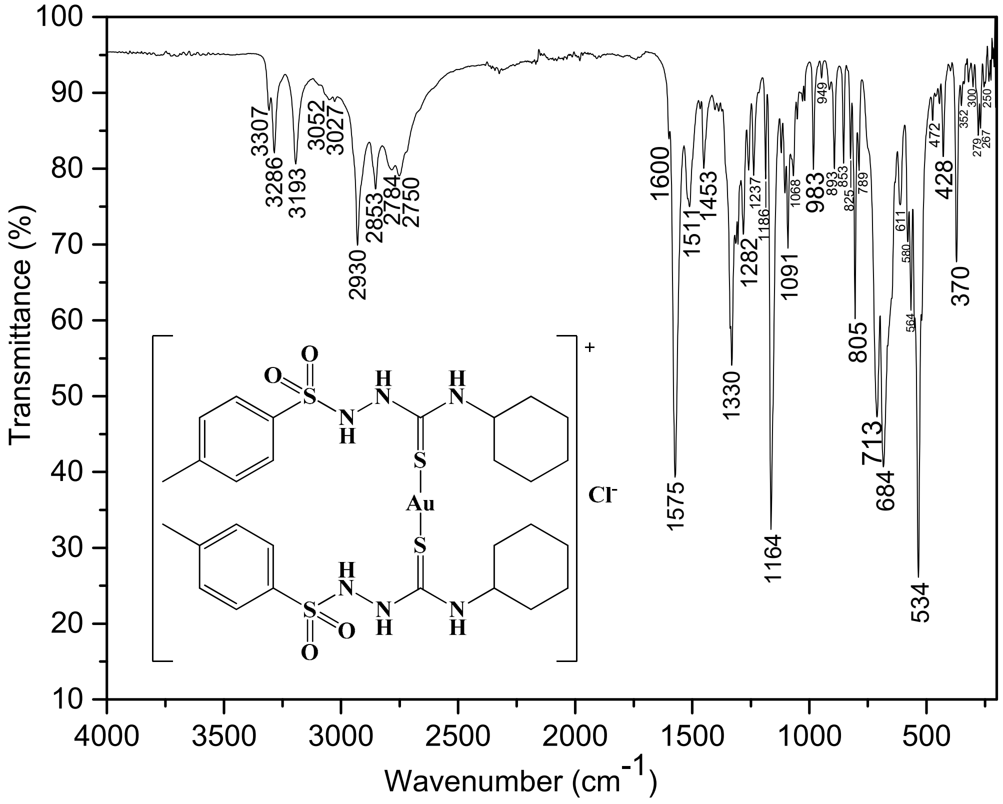

**Figure S1.10** – IR (ATR) spectrum of [Au(HL2$^{Ph}$)$_2$]Cl (**Au2**).

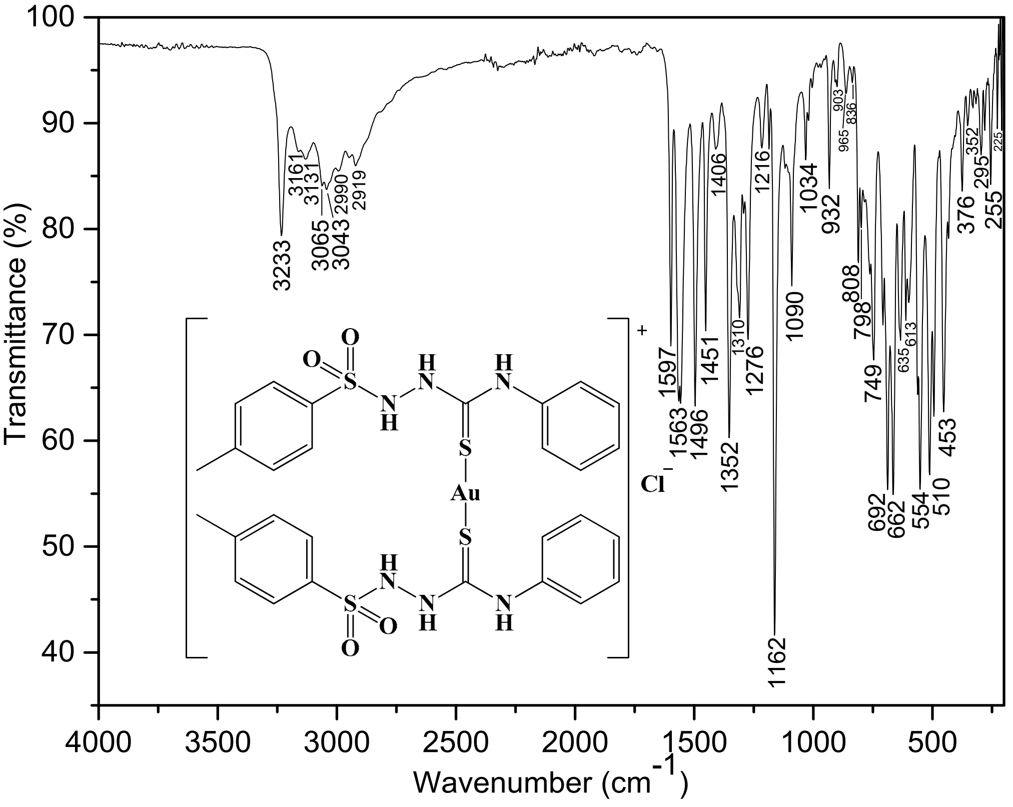

**Figure S1.11** – IR (ATR) spectrum of [Au(HL3$^{FPh}$)$_2$]Cl (**Au3**).

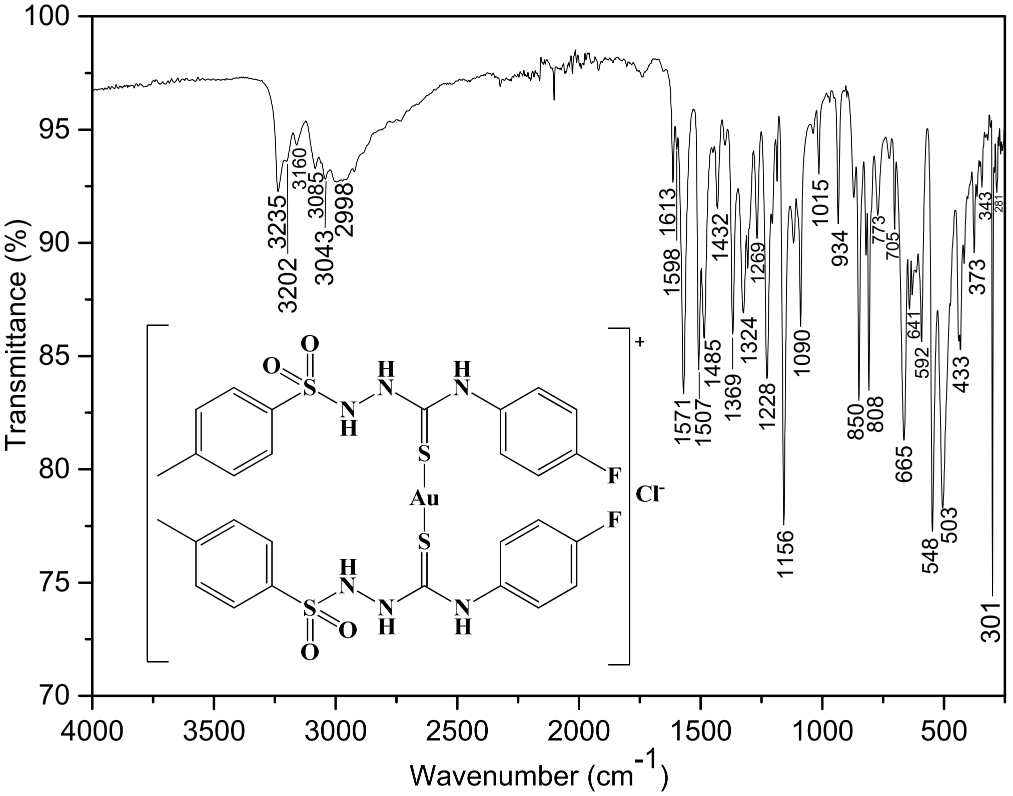


**Figure S1.12** – IR (ATR) spectrum of [Au(HL4$^{ClPh}$)$_2$]Cl (**Au4**).

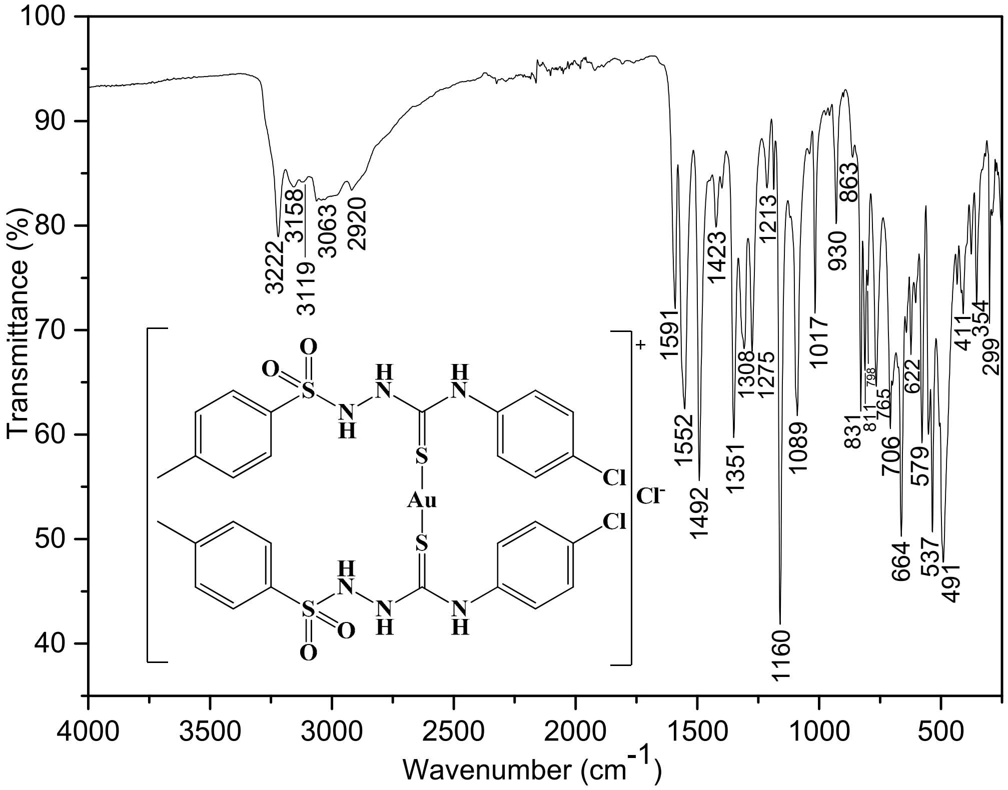

**Figure S1.13** – IR (ATR) spectrum of [Au($HL5^{NO2Ph}$)$_2$]Cl (**Au5**).

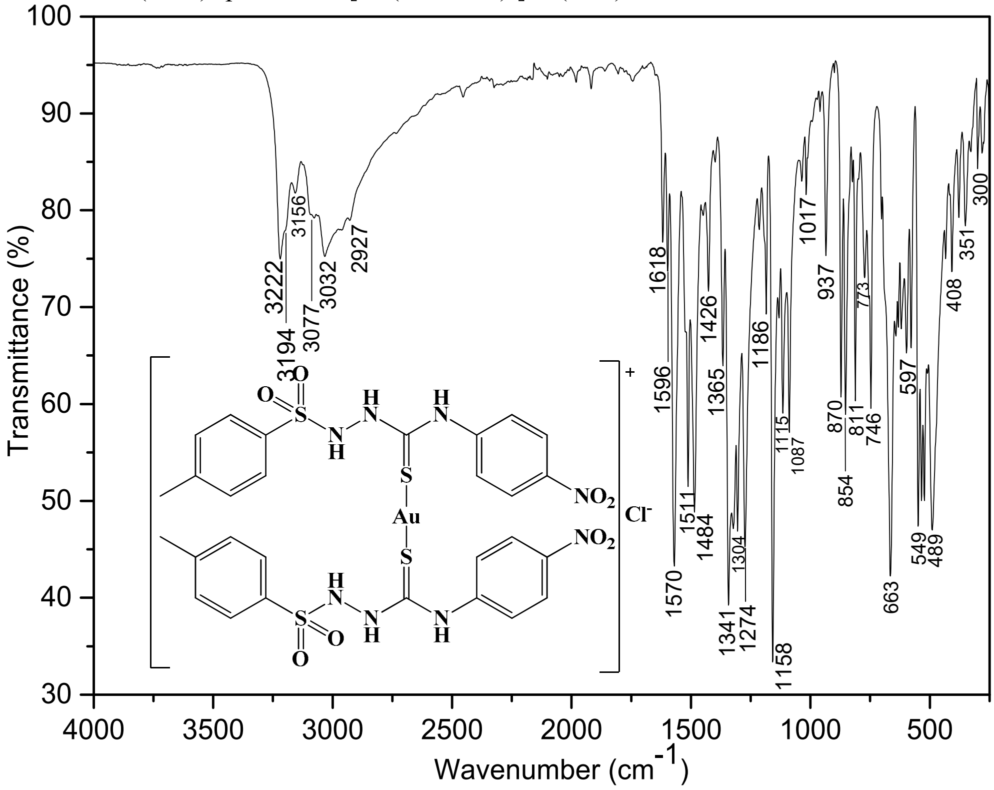


**Figure S1.14** - IR (ATR) spectrum of [Au($HL6^{Al}$)$_2$]Cl (**Au6**).

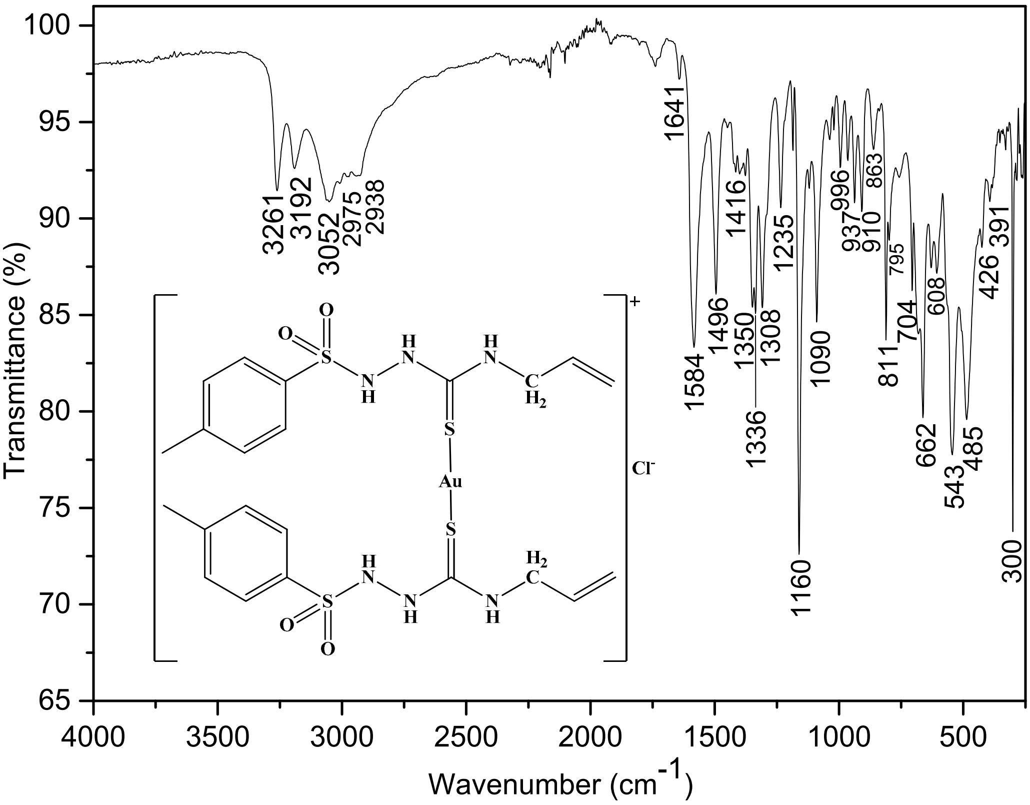

**Table S1 -** Selected absorption bands in the infrared region for free ligands and complexes.

| | Selected bands/ $cm^{-1}$ | | | | | |
|---|---|---|---|---|---|---|
| **Compound** | ν (N-H) | ν(-NH-CS-NH-) | ν(N-O) | $\nu_{ass}$(S=O) | $\nu_s$(S=O) | ν(C=S) |
| **$HL1^{Ch}$** | 3371/3228/3167 | 1597 | — | 1352/1330 | 1166 | 808 |
| **$HL2^{Ph}$** | 3329/3228/3136 | 1597 | — | 1342/1328 | 1165 | 806 |
| **$HL3^{FPh}$** | 3320/3240/3133 | 1596 | — | 1333 | 1156 | 814 |
| **$HL4^{ClPh}$** | 3306/3290/3145 | 1600/1584 | — | 1341 | 1161 | 806 |
| **$HL5^{NO2Ph}$** | 3308/3240/3136 | 1615/1597 | 1424 | 1331 | 1158 | 807 |
| **$HL6^{Al}$** | 3358/3160 | 1600 | — | 1348 | 1170 | 809 |
| **Ag3** | 3233/3183/3156 | 1609 | 1416 | 1332 | 1160 | 786 |
| **Ag4** | 3228/3184/3166 | 1593 | 1409 | 1332 | 1160 | 784 |
| **Ag5** | 3385/3306/3217/3155 | 1619/1596 | 1418/1400 | 1318 | 1154 | — |
| **Ag6** | 3265/3225 | 1646 | 1418 | 1327 | 1134 | 781 |
| **Au1** | 3307/3286/3193 | 1600 | — | 1330 | 1164 | 789 |
| **Au2** | 3233/3161/3131 | 1597 | — | 1352 | 1162 | 798 |
| **Au3** | 3235/3202/3160 | 1613/1598 | — | 1324 | 1156 | 773 |
| **Au4** | 3222/3158/3119 | 1591 | — | 1351 | 1160 | 798 |
| **Au5** | 3222/3194/3156 | 1641 | 1426 | 1350 | 1160 | 795 |
| **Au6** | 3261/3192 | 1618/1596 | — | 1341 | 1158 | 773 |

**Figure S2.1 –** $^{1}$H NMR spectrum of HL3$^{FPh}$ in DMSO-$d_6$.

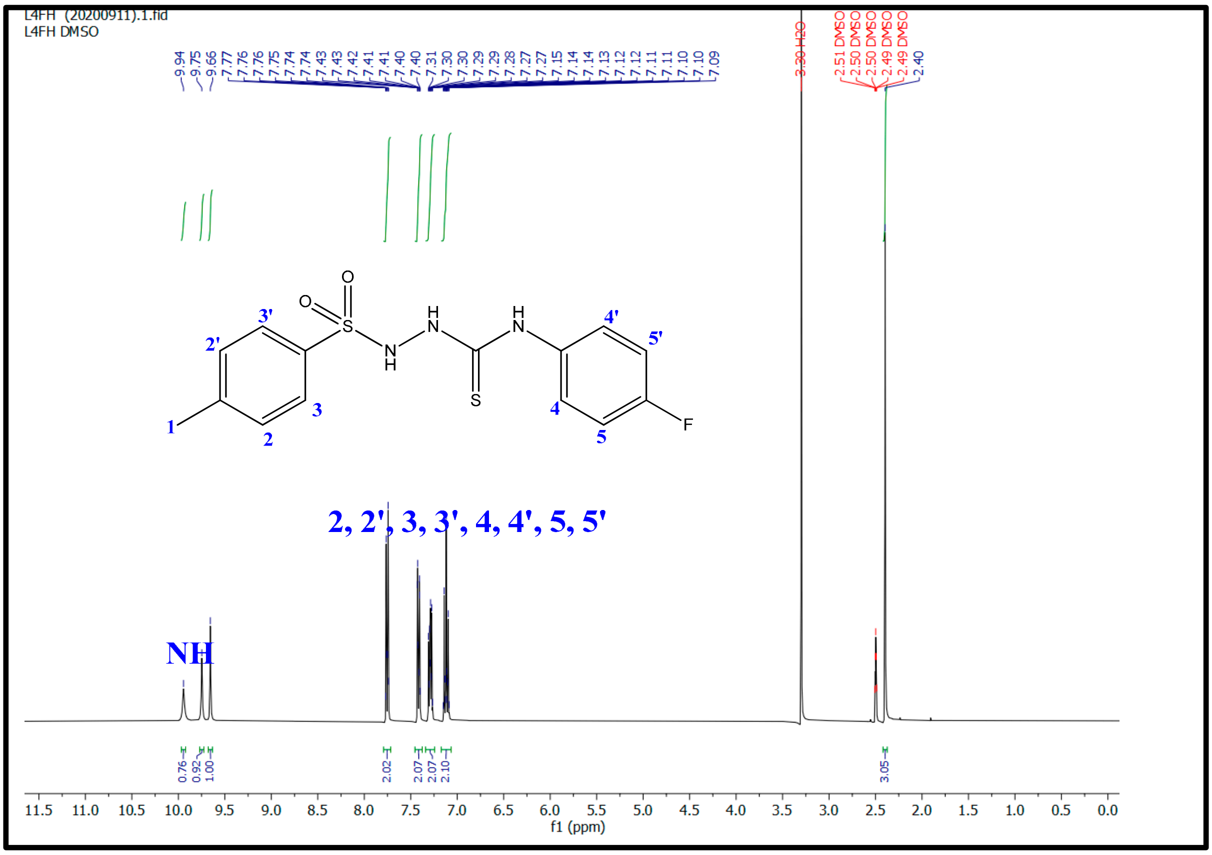


**Figure S2.2 –** $^{1}$H NMR spectrum of HL4$^{ClPh}$ in DMSO-$d_6$.

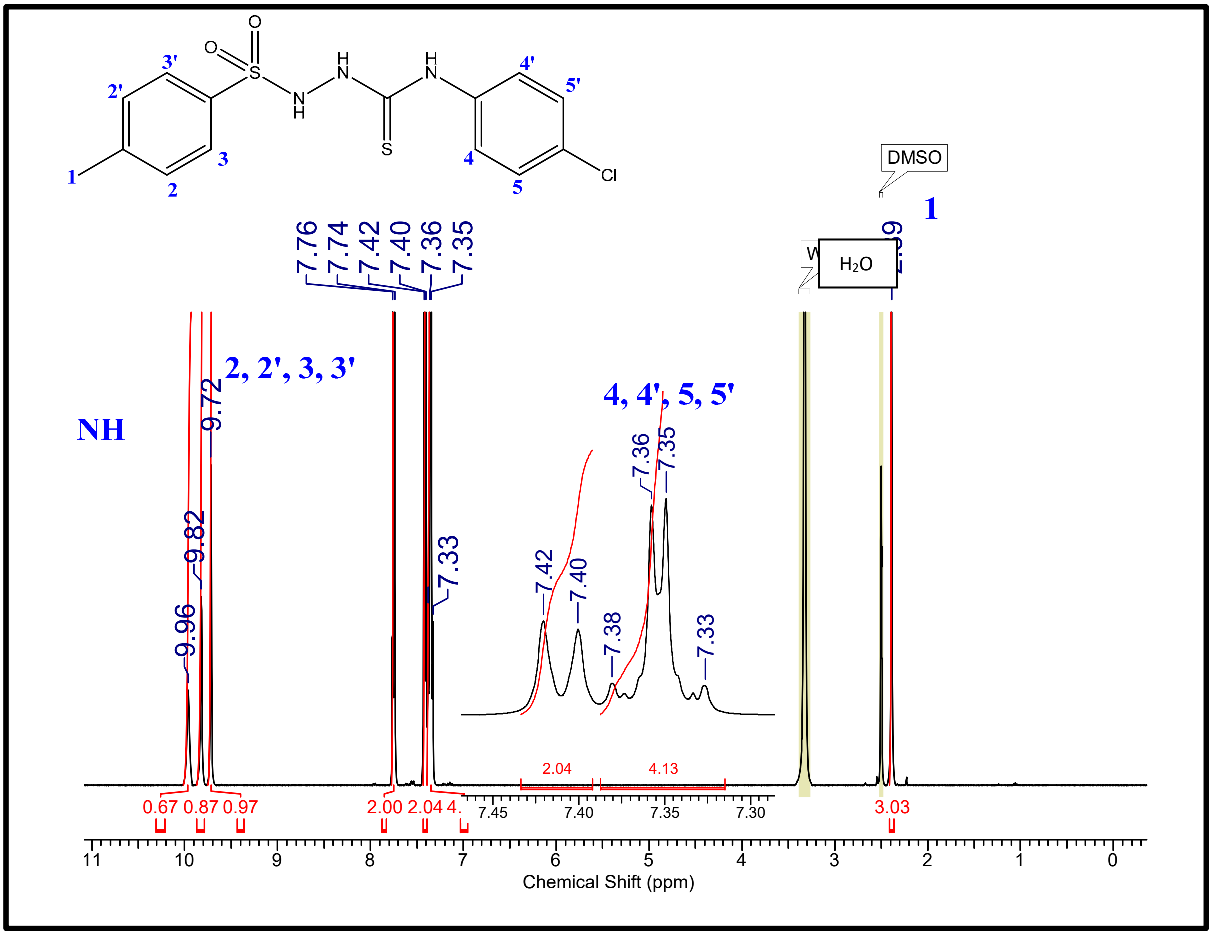

**Figure S2.3** – $^{1}$H NMR spectrum of HL**5**$^{NO2Ph}$ in DMSO-$d_6$.

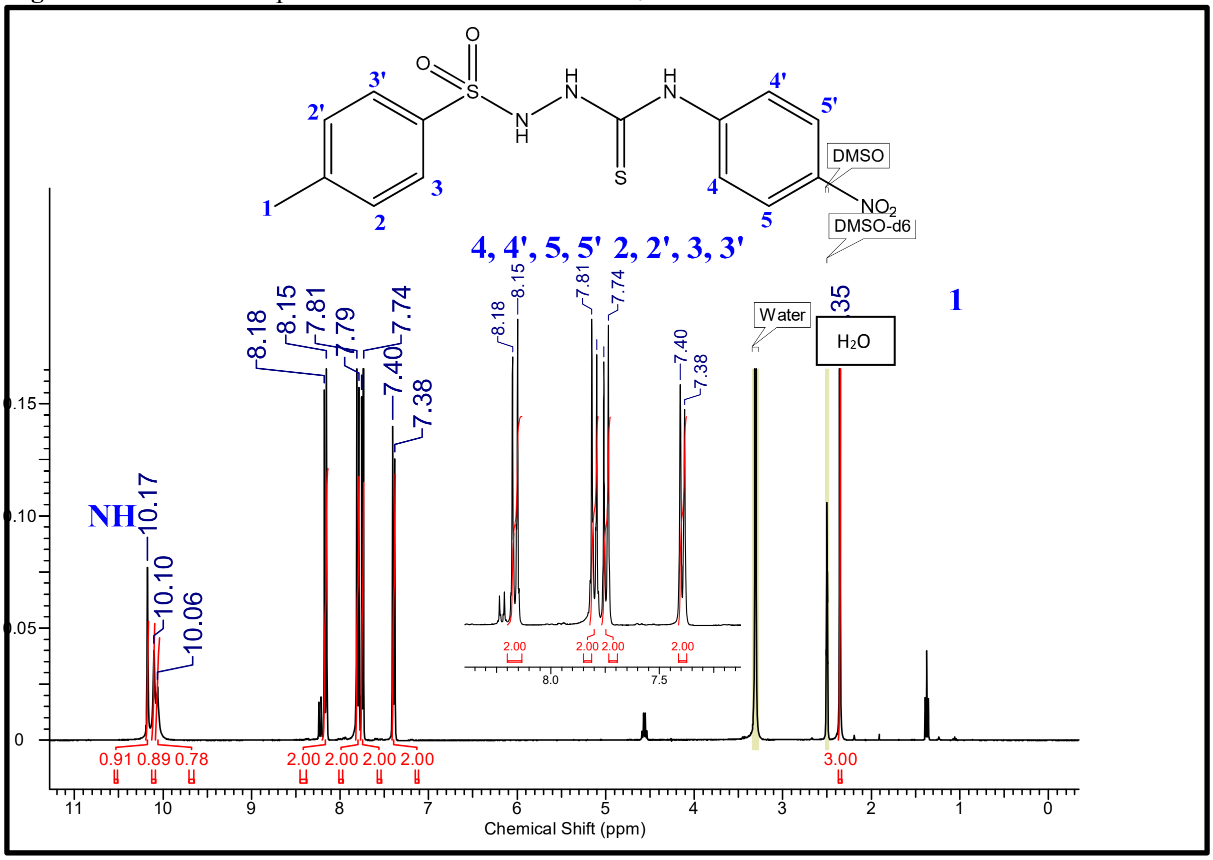

**Figure S2.4** – $^{1}$H NMR spectrum of HL**6**$^{Al}$ in DMSO-$d_6$.

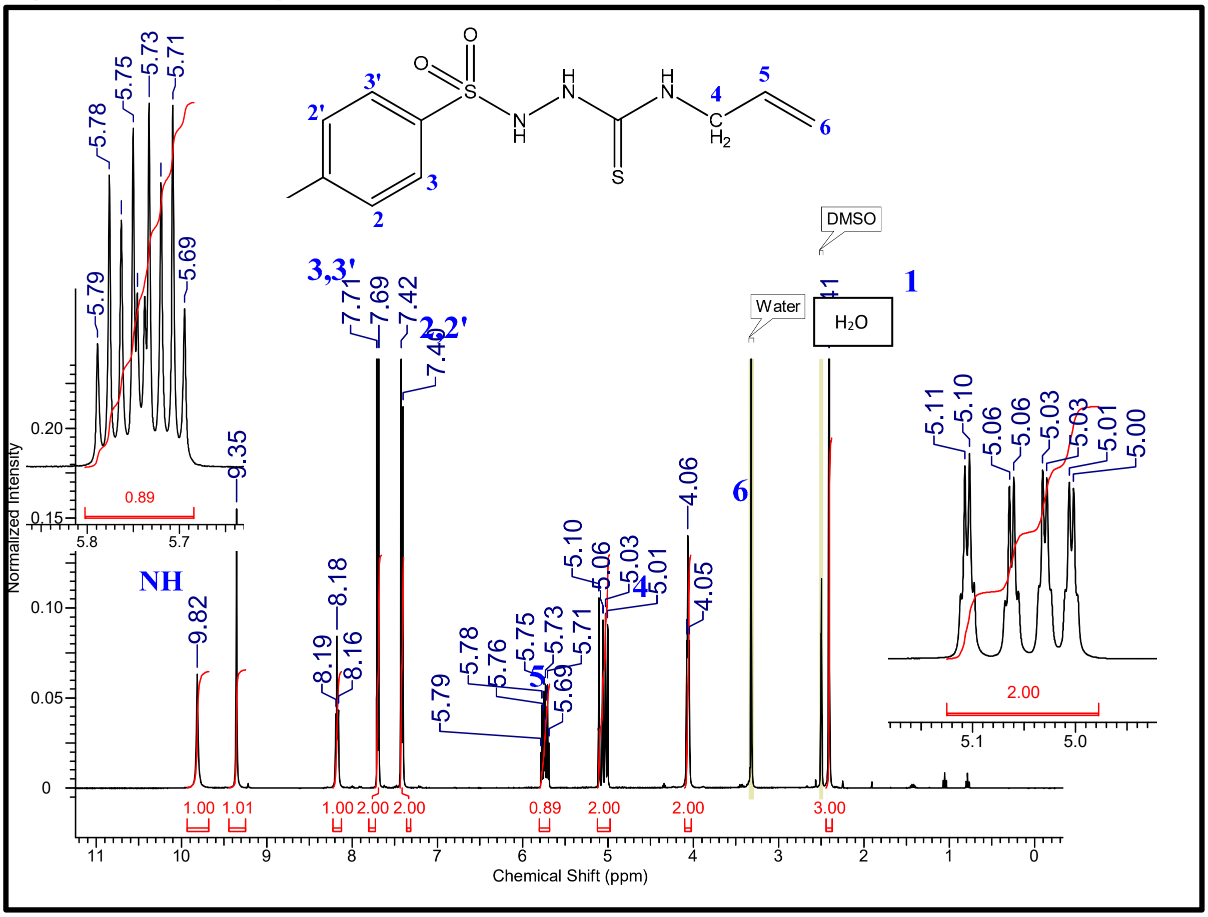

**Figure S2.5 –** $^{19}$F NMR spectrum (377 MHz) of H**L3**$^{\mathbf{FPh}}$ in DMSO-$d_6$.

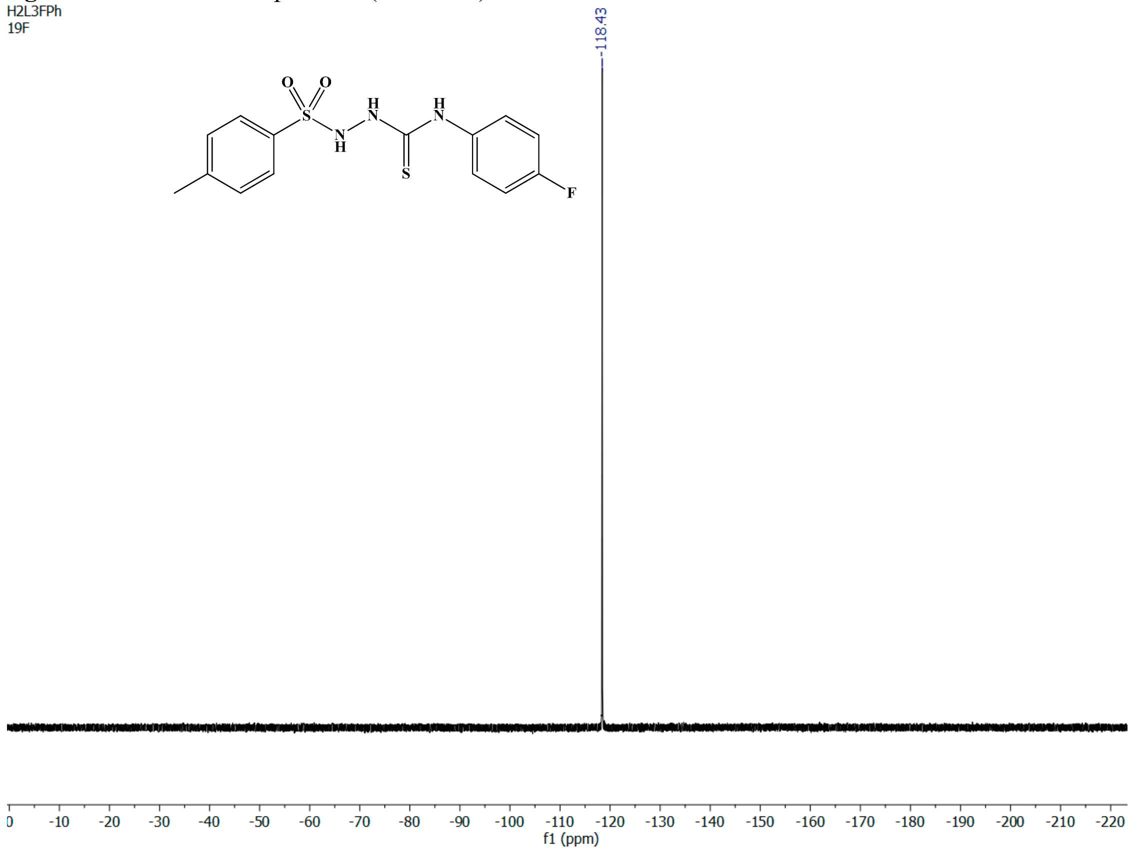


**Figure S2.6 –** $^{1}$H NMR spectrum of [Ag(HL3$^{FPh}$)$_2$]$NO_3$ (**Ag3**) in DMSO-$d_6$.

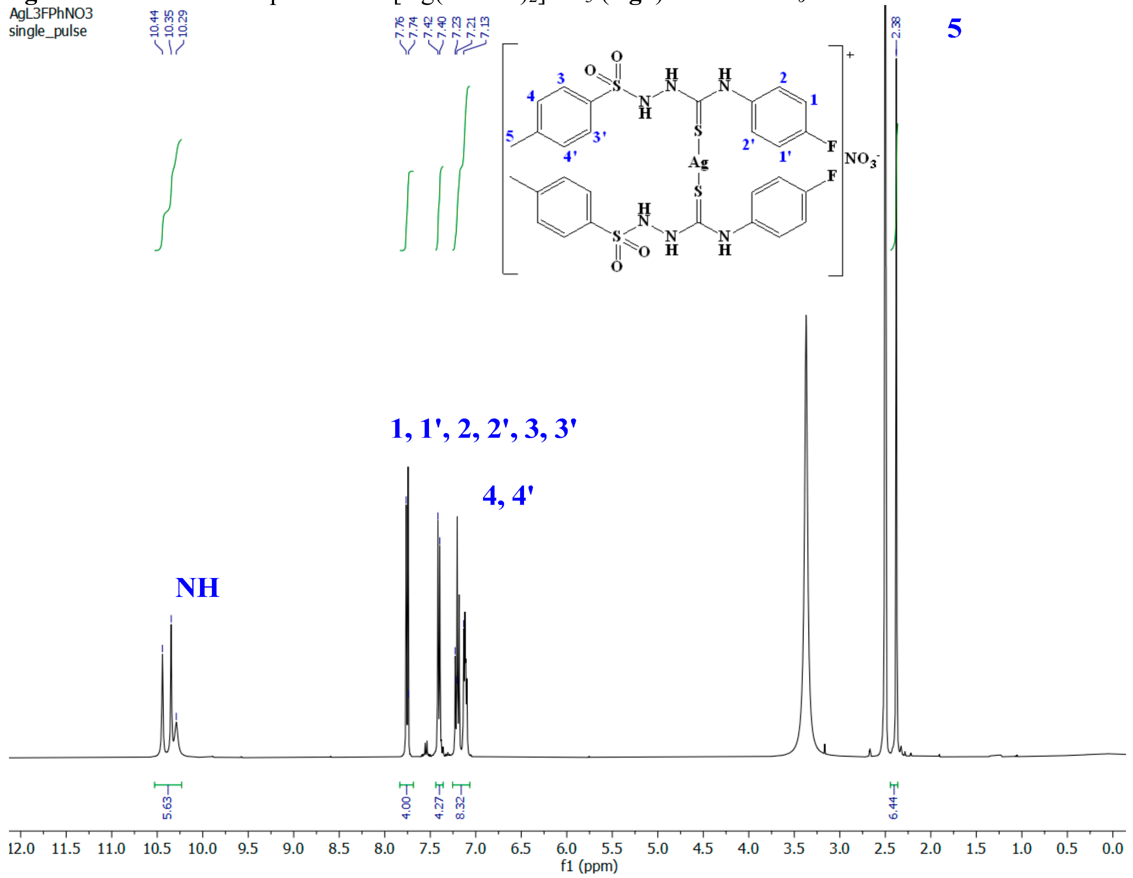

**Figure S2.7 –** $^{1}$H NMR spectrum of $[Ag(HL4^{ClPh})_2]NO_3$ (**Ag4**) in DMSO-$d_6$.

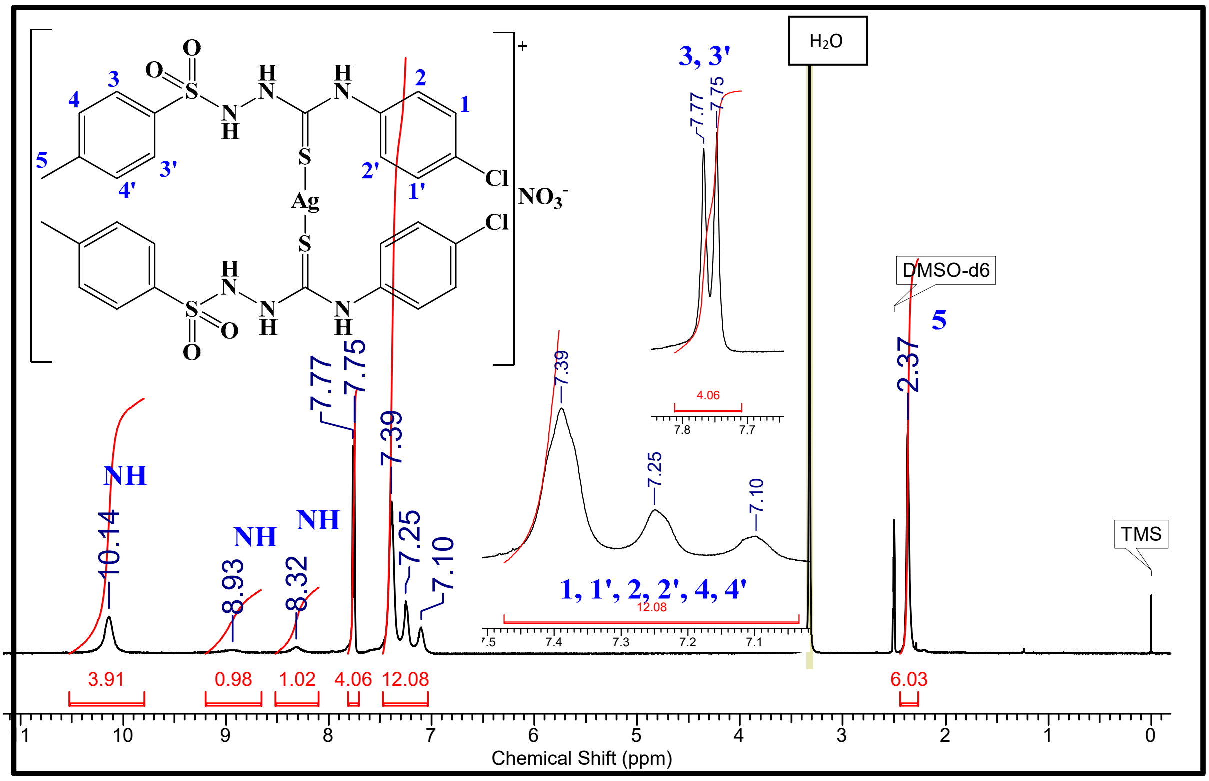


**Figure S2.8 –** $^{1}$H NMR spectrum of $[Ag(HL5^{NO2Ph})_2]NO_3$ (**Ag5**) in DMSO-$d_6$.

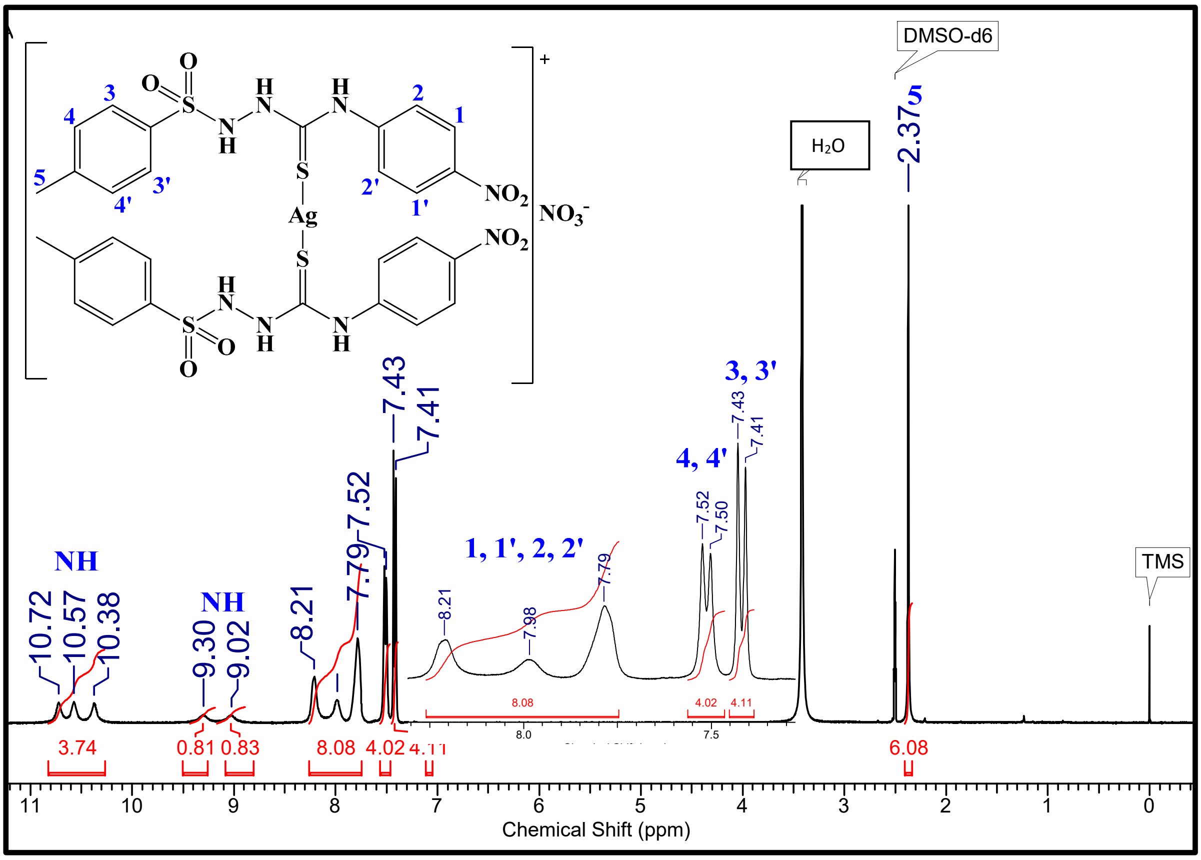

**Figure S2.9 –** $^{1}H$ NMR spectrum of $[Ag(HL6^{Al})_2]NO_3$ (**Ag6**) in DMSO-$d_6$.

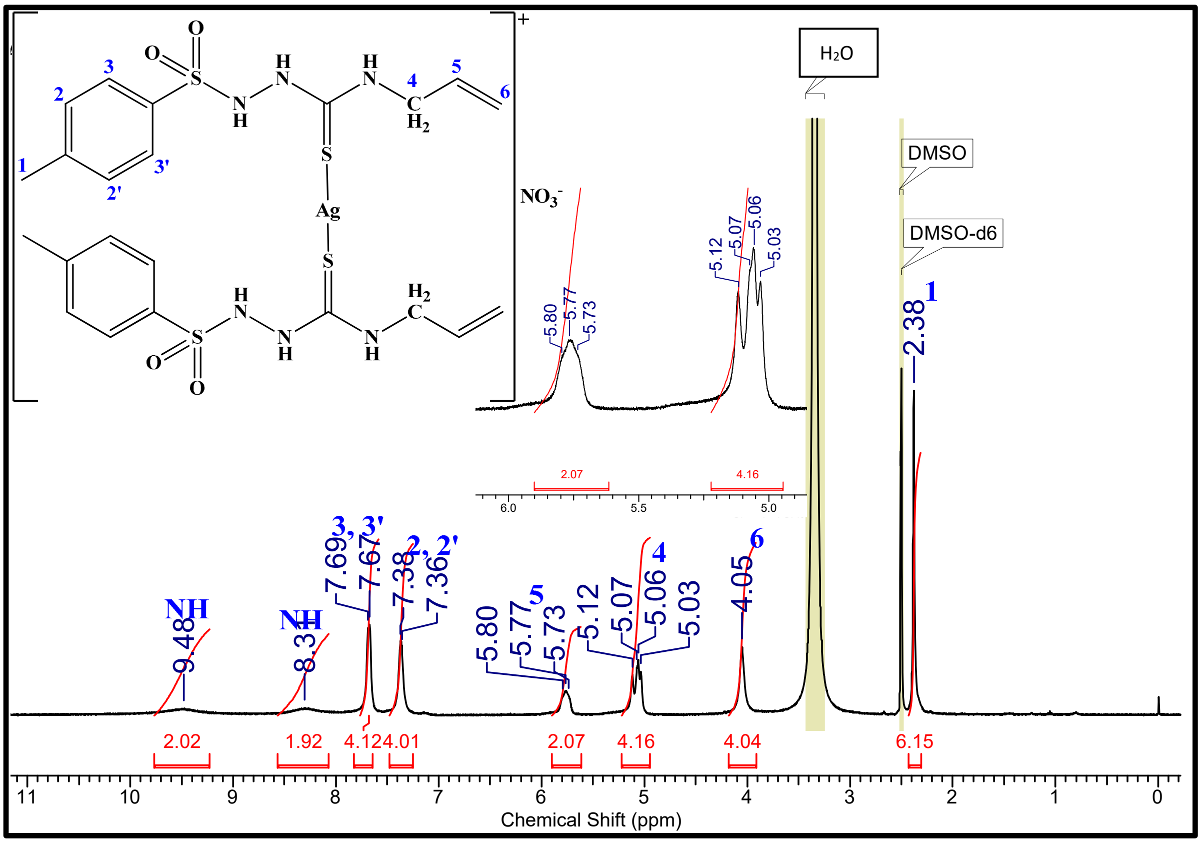


**Figure S2.10 –** $^{1}H$ NMR spectrum of $[Au(HL1^{Ch})_2]Cl$ (**Au1**) in DMSO-$d_6$.

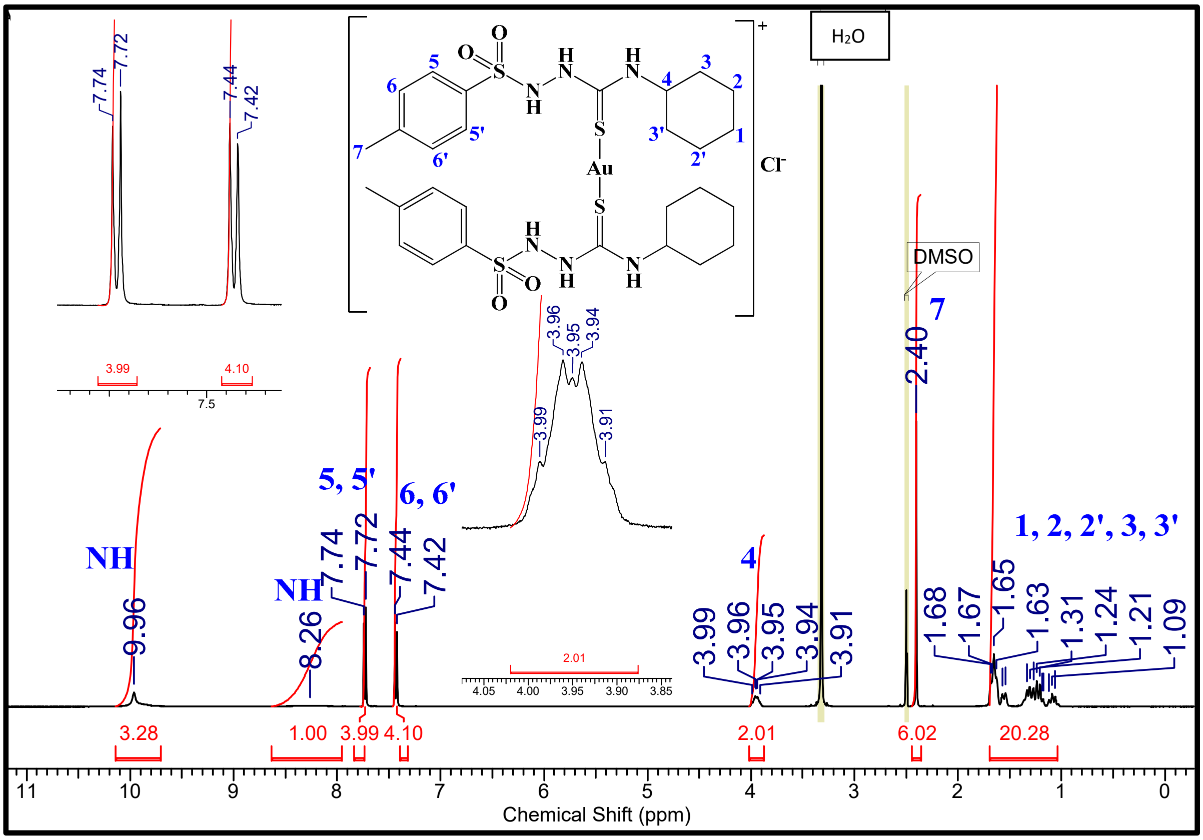

**Figure S2.11 –** $^{1}$H NMR spectrum of $[Au(HL2^{Ph})_2]Cl$ (**Au2**) in DMSO-$d_6$.

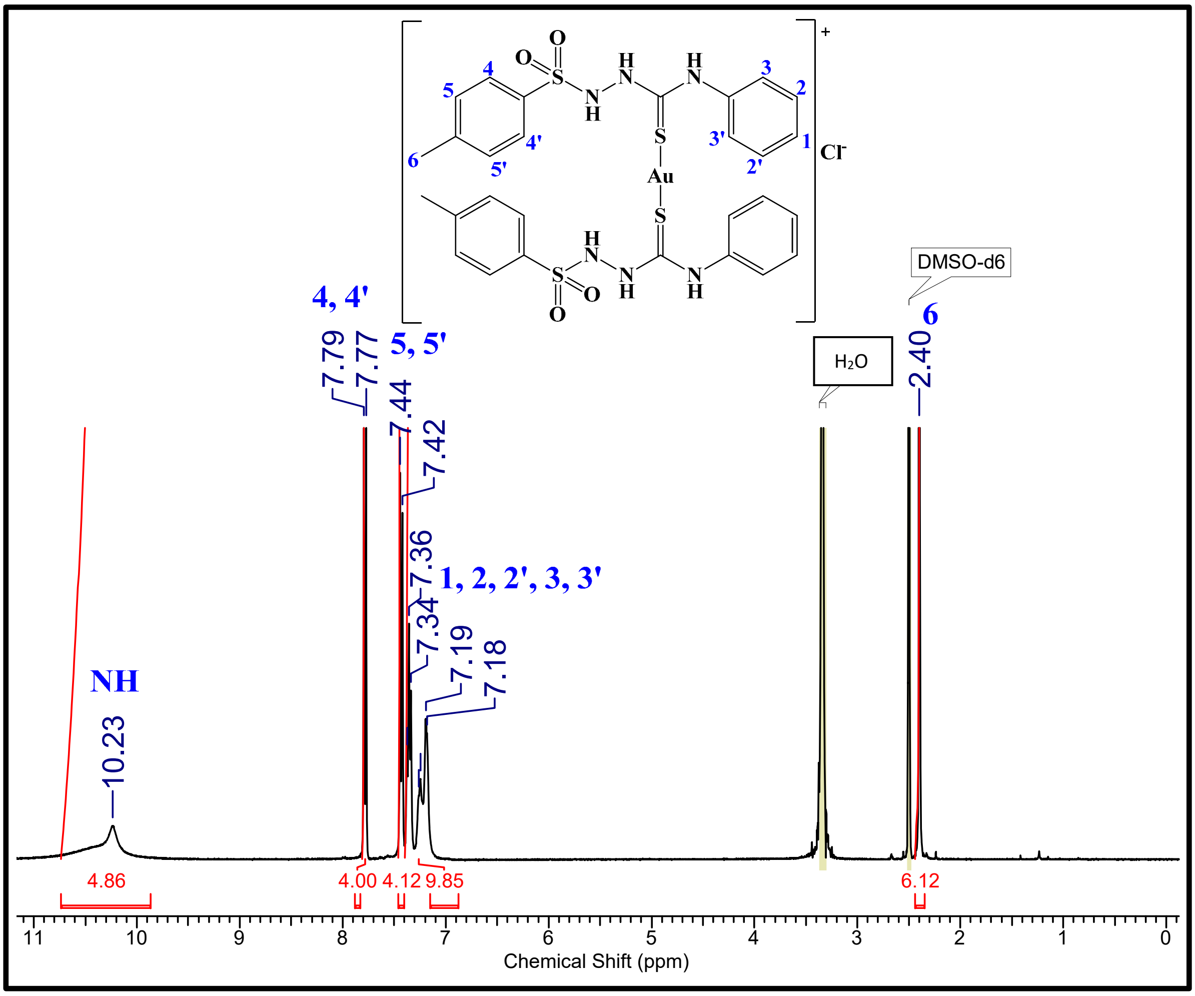


**Figure S2.12 –** $^{1}$H NMR spectrum of $[Au(HL3^{FPh})_2]Cl$ (**Au3**) in DMSO-$d_6$.

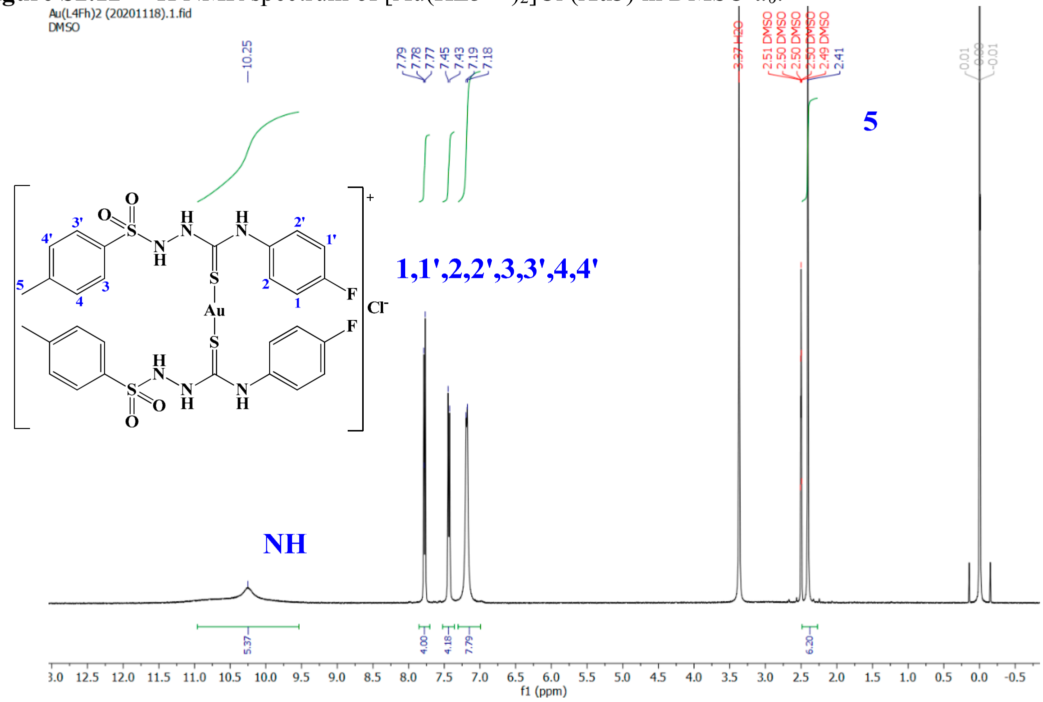

**Figure S2.13 –** $^{1}$H NMR spectrum of [Au(HL4$^{ClPh}$)$_2$]Cl (**Au4**) in DMSO-$d_6$.

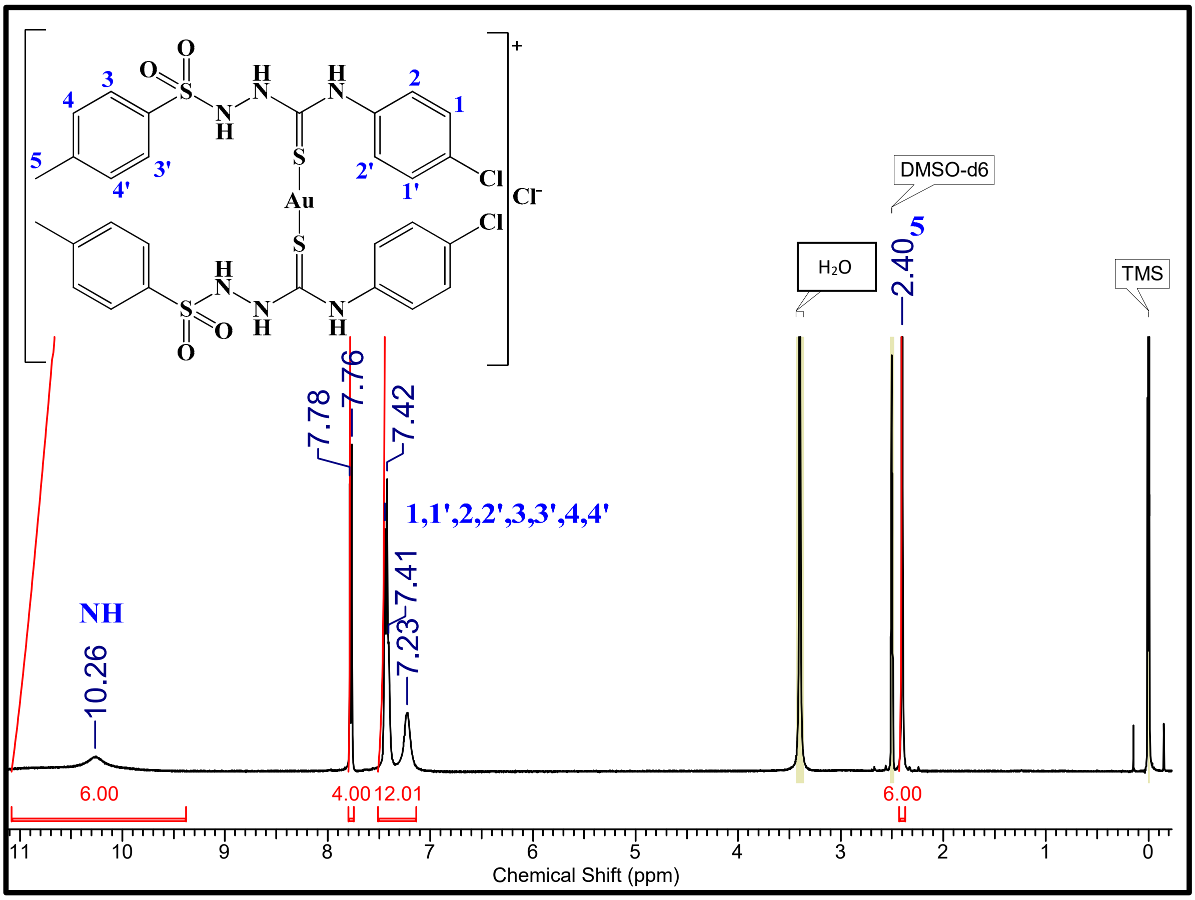


**Figure S2.14 –** $^{1}$H NMR spectrum of [Au(HL5$^{NO2Ph}$)$_2$]Cl(**Au5**) in DMSO-$d_6$.

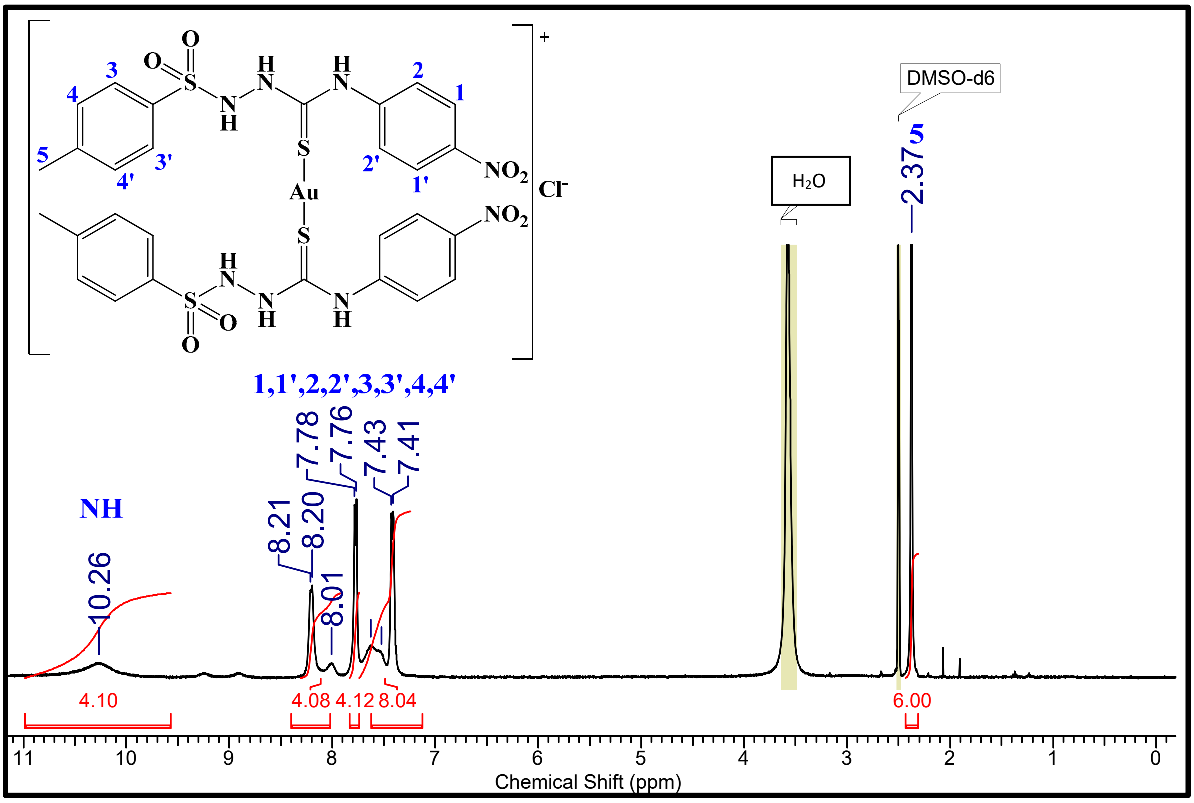

**Figure S2.15 –** $^{1}$H NMR spectrum of $[Au(HL6^{Al})_2]Cl$ (**Au6**) in DMSO-$d_6$.

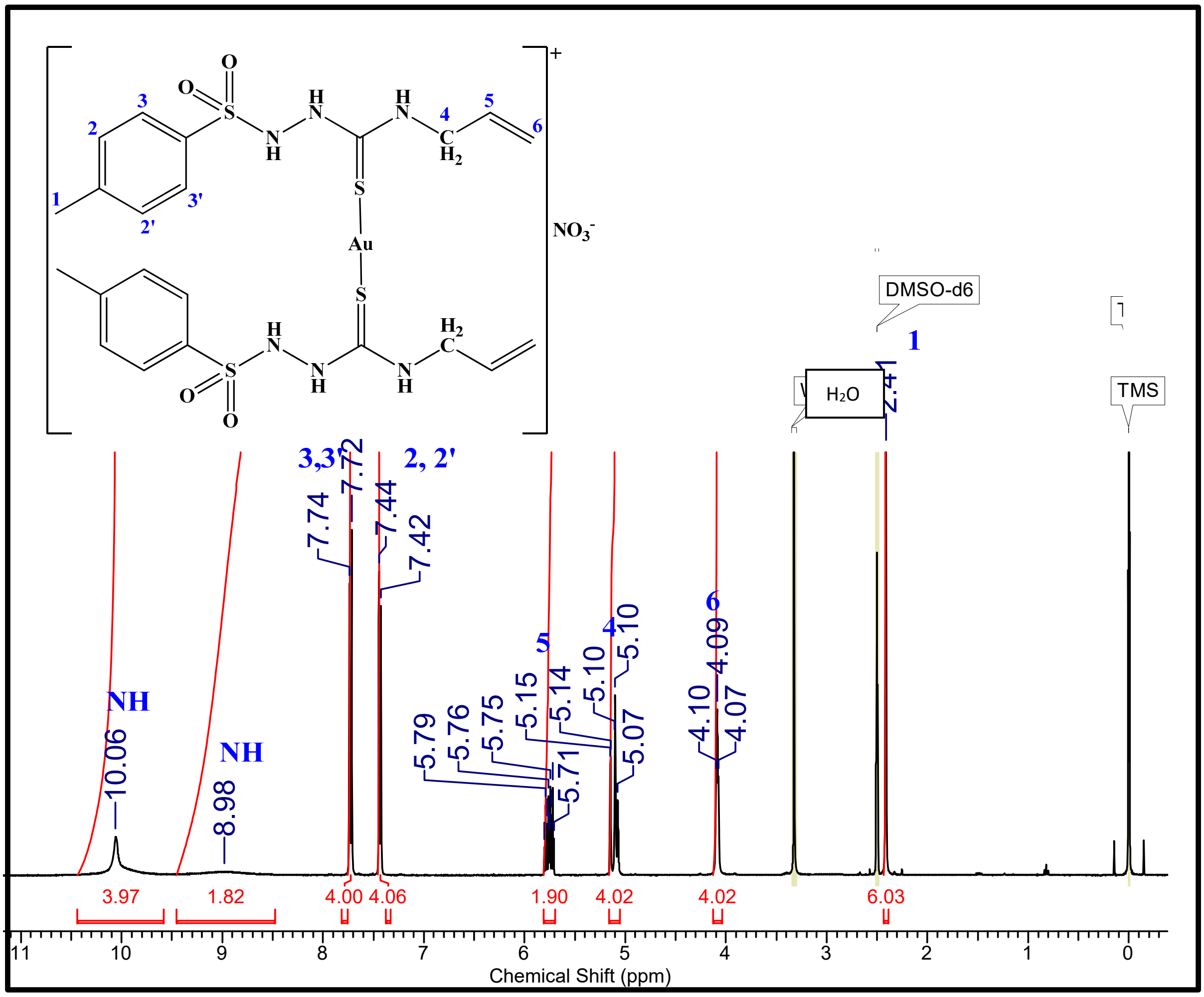

**Figure S2.16 –** $^{19}$F NMR spectrum (377 MHz) of $[Ag(HL3^{FPh})_2]NO_3$ (**Ag3**) in DMSO-$d_6$.

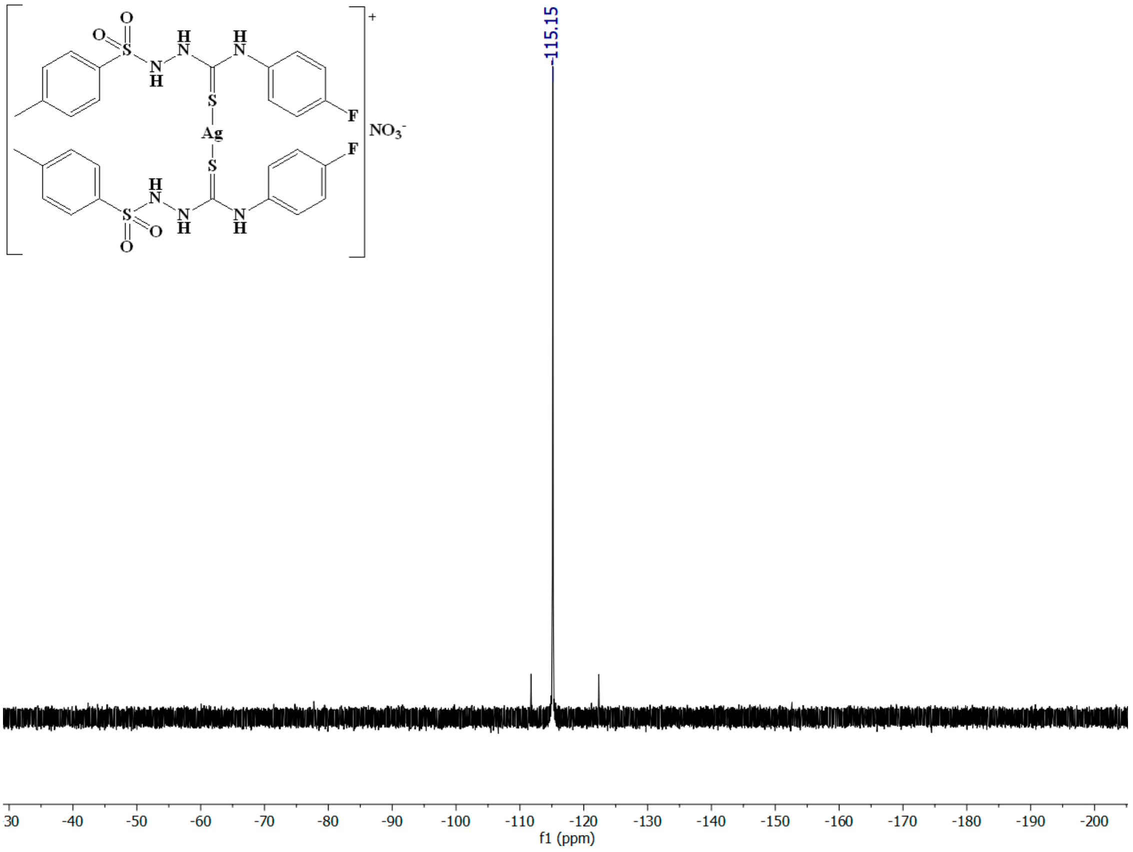


**Figure S2.17 –** $^{19}$F NMR spectrum (377 MHz) of $[Au(HL3^{FPh})_2]Cl$ (**Au3**) in DMSO-$d_6$.

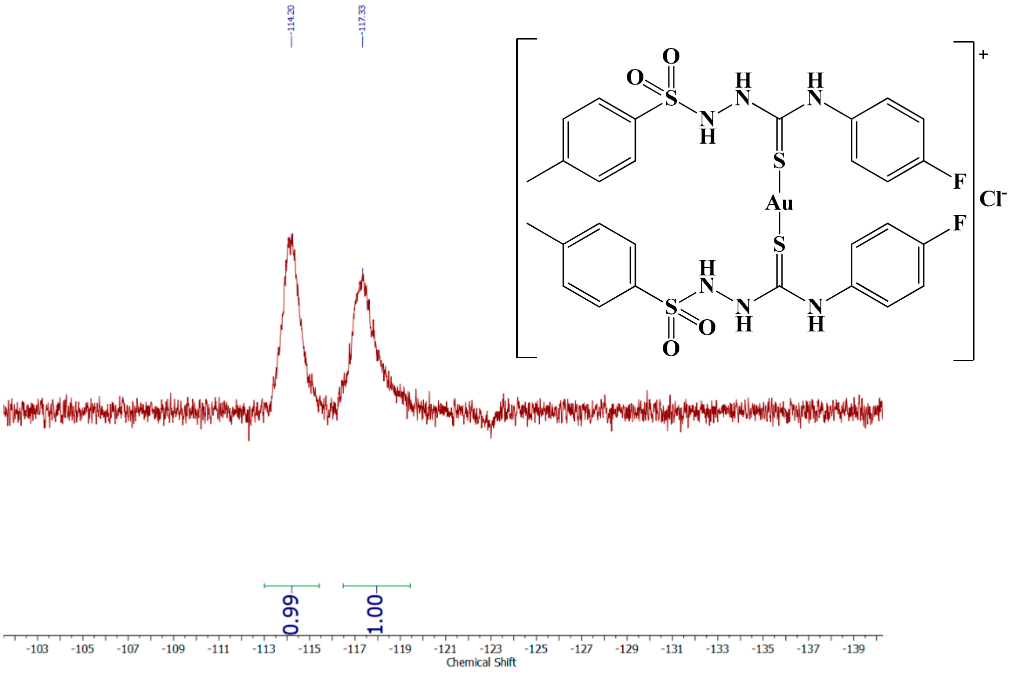

**Figure S3.1** – ESI(+) MS spectrum of $[Ag(HL3^{FPh})_2]NO_3$ (**Ag3**).

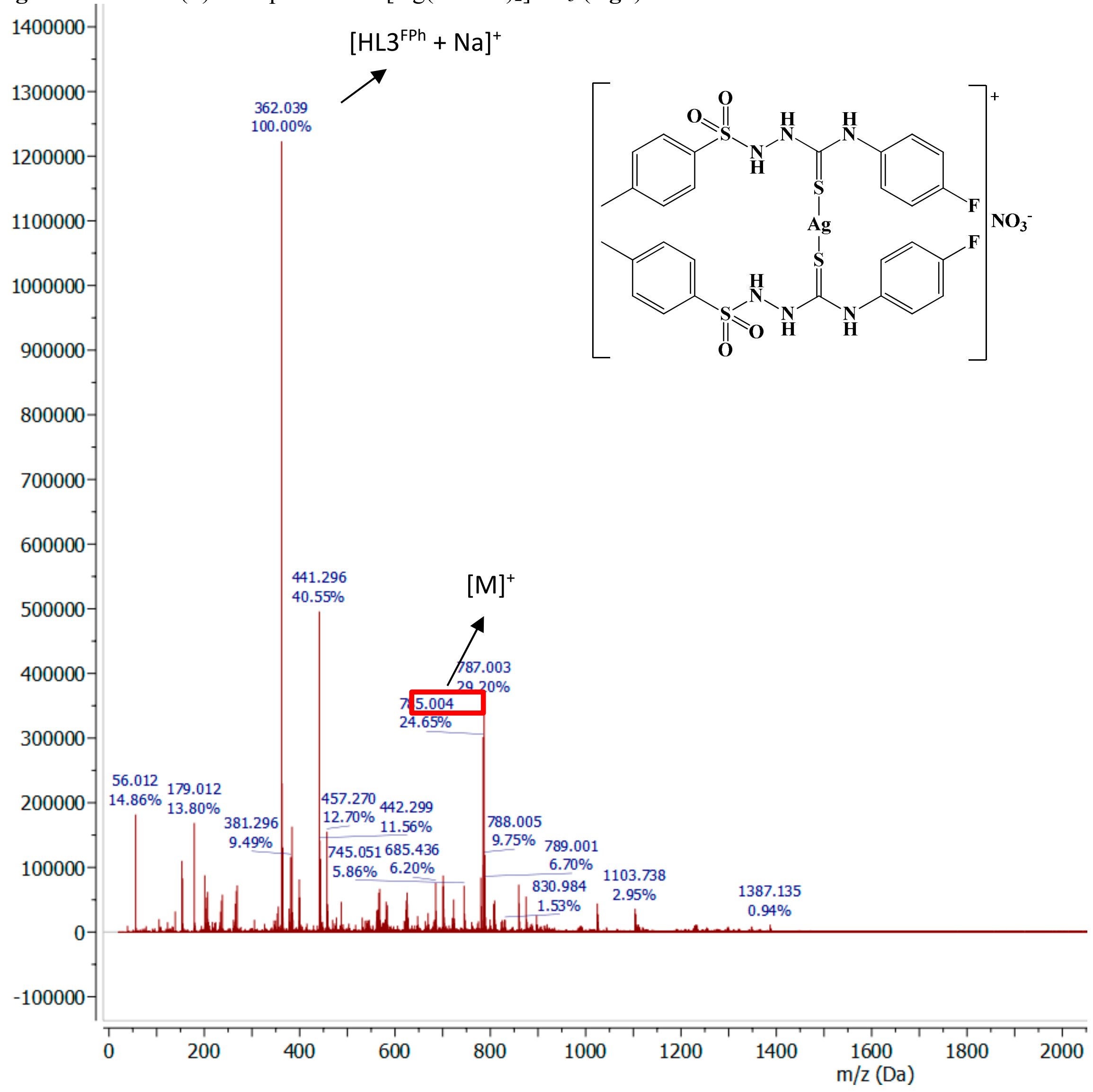

**Figure S3.2** – ESI(+) MS spectrum of [Au(HL1$^{Ch}$)$_2$]Cl (**Au1**).

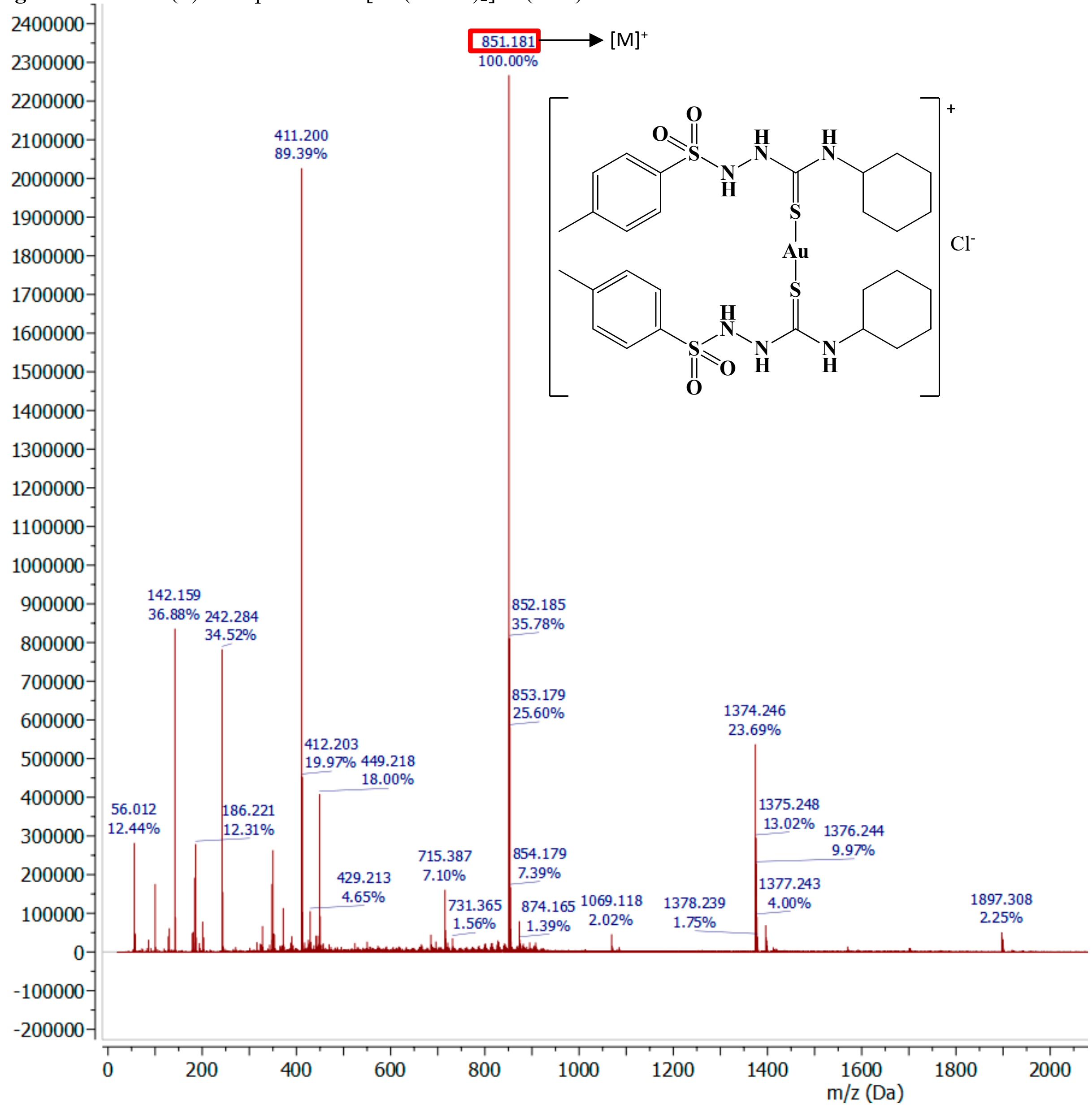

**Figure S3.27** – ESI(+) MS spectrum of [Au(HL2$^{Ph}$)$_2$]Cl (**Au2**).

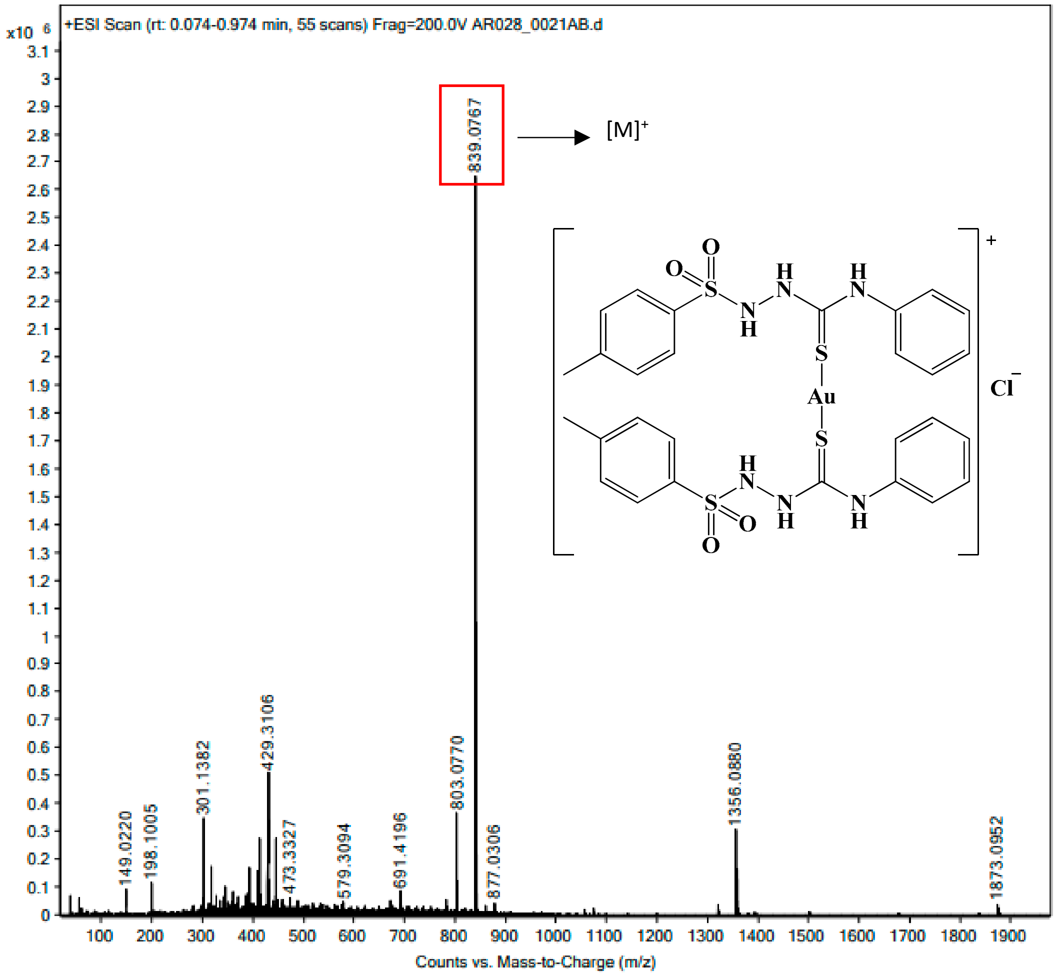

**Figure S3.28 –** ESI(+) MS spectrum of [Au(HL3$^{FPh}$)$_2$]Cl (**Au3**).

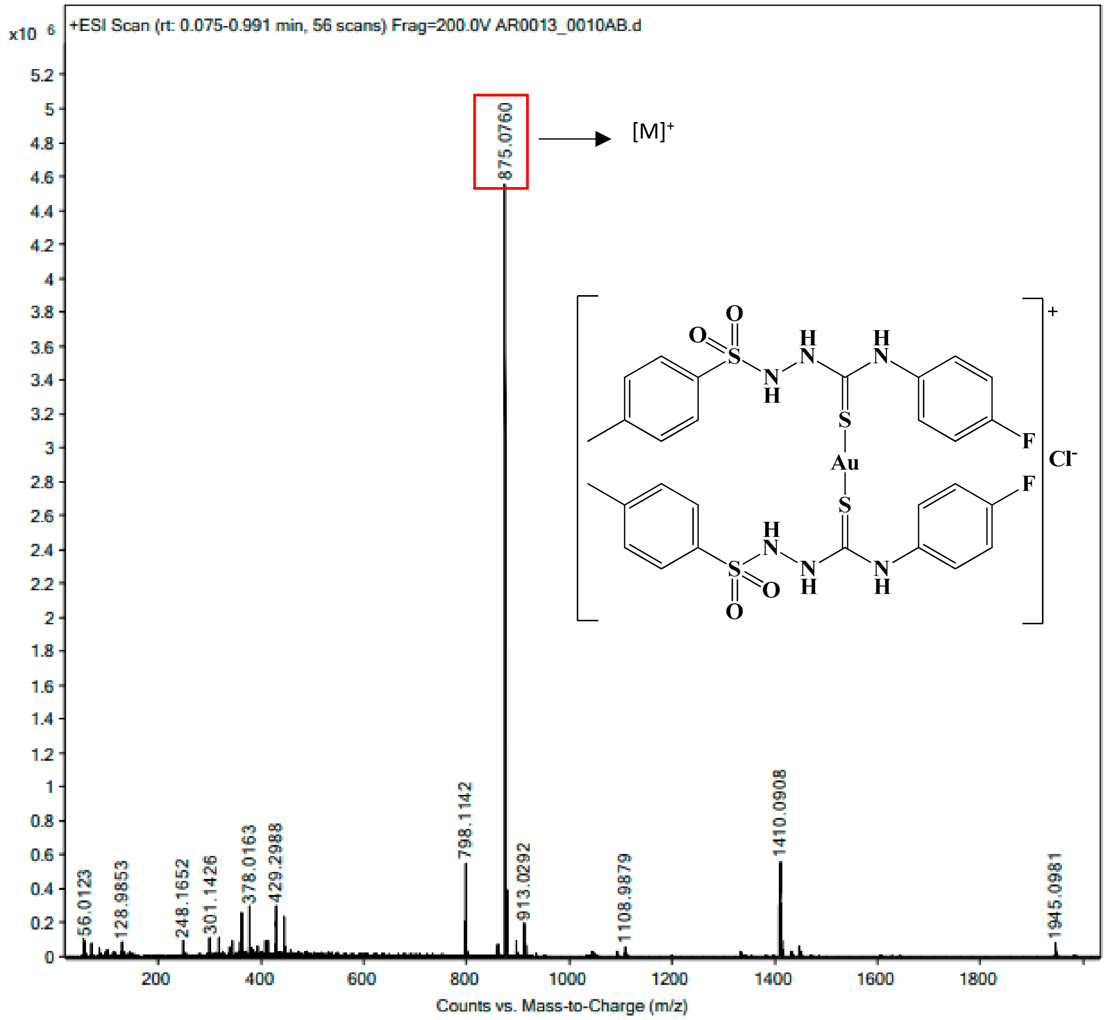

**Figure S3.29 –** ESI(+) MS spectrum of [Au(HL4$^{ClPh}$)$_2$]Cl (**Au4**).

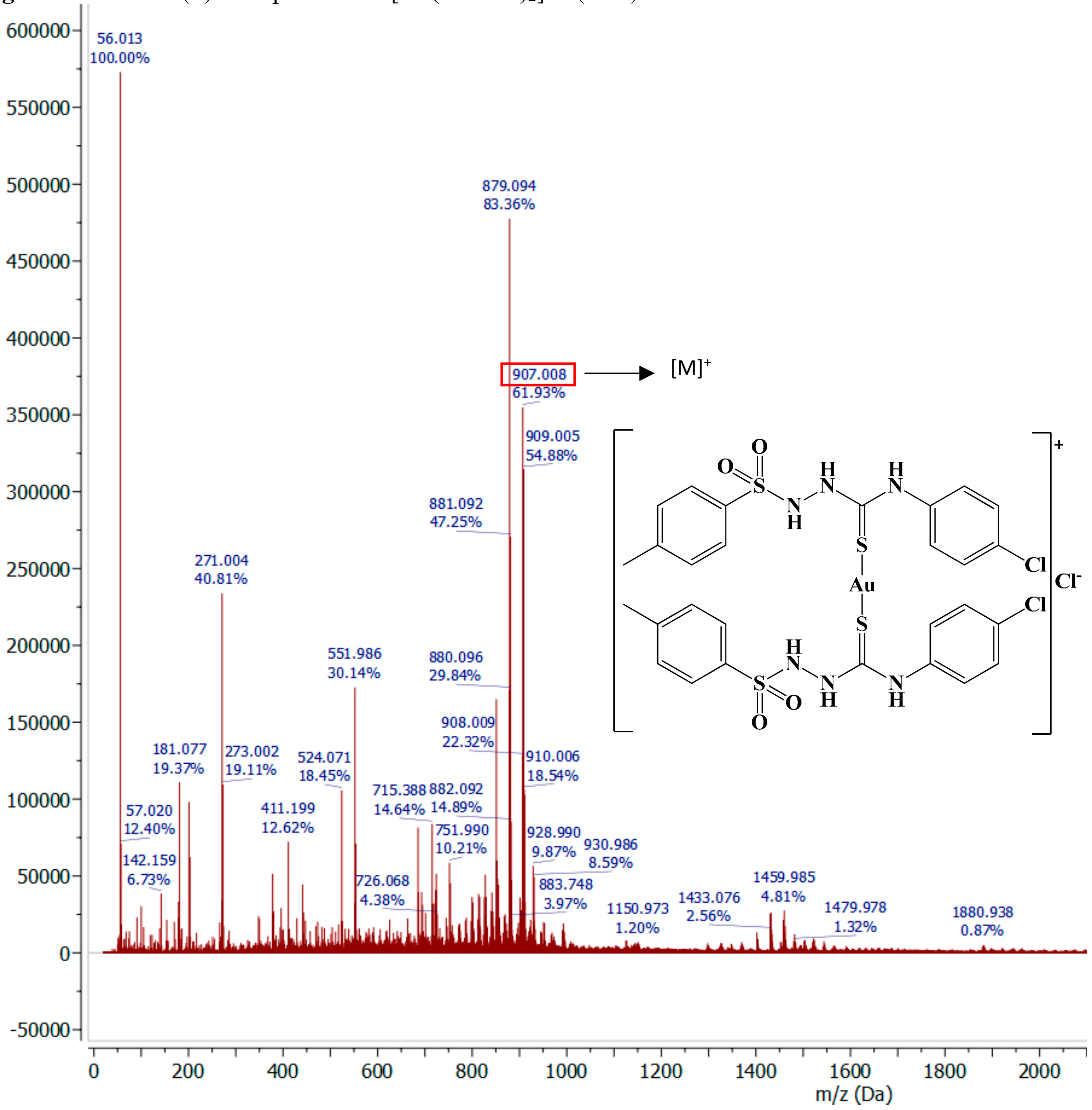

**Figure S3.30 –** ESI(+) MS spectrum of [Au($HL6^{Al}$)$_2$]Cl (**Au6**).

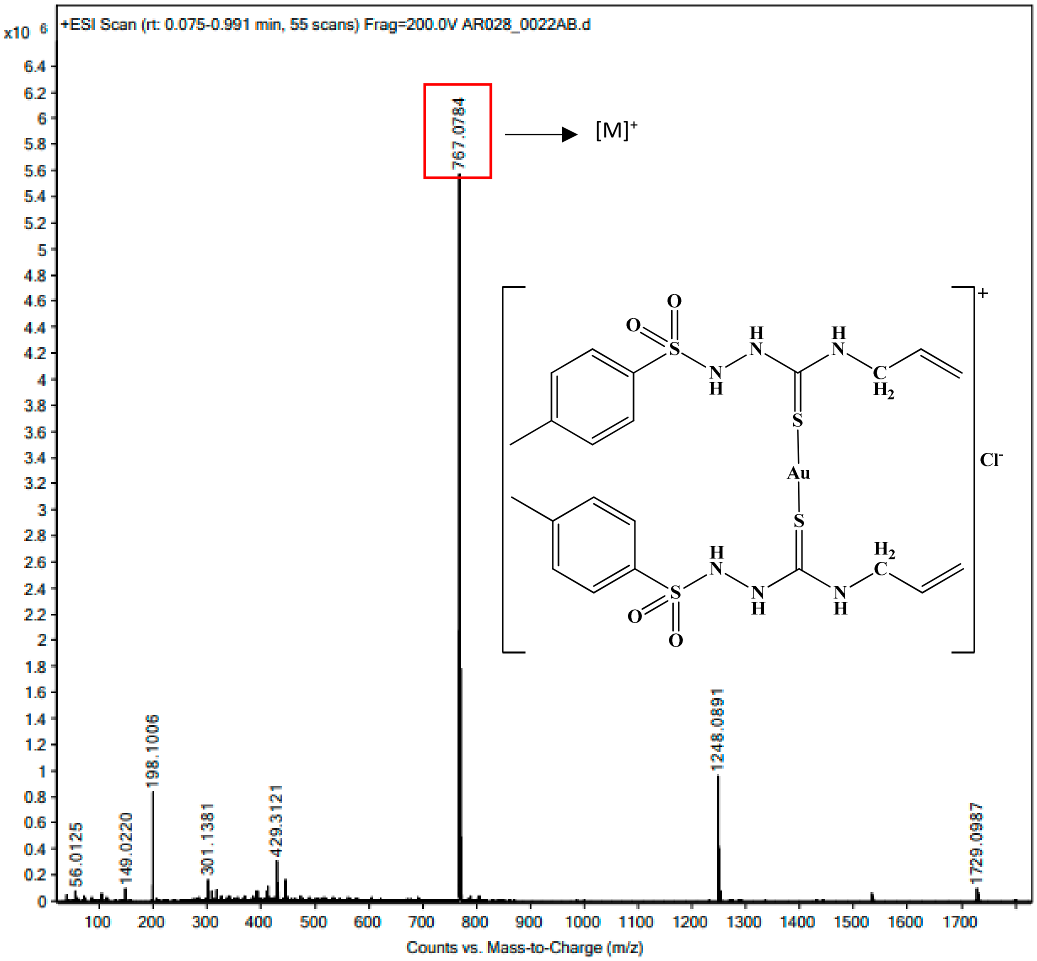

**Figure S4.1 –** Hydrogen bonds involved in the crystalline structure of the **HL3**$^{\mathbf{FPh}}$ ligand. Intermolecular hydrogen bonds: [N(1)···O(1) = 2.910(3) Å, N(1)-H(1)···O(1)= 115.4º], [N(2)···S(1) = 3.311(2) Å, N(2)-H(2)···S(1) = 160.8º], [N(1)···O(2) = 3.420(3) Å, N(1)-H(1)···O(2) = 98.1º], [N(3)···O(2) = 2.959(2) Å, N(3)-H(3)···O(2) = 147.7º]. Intramolecular hydrogen bonding: [N(3)···N(1) = 2.659(3) Å, N(3)-H(3)···N(1)= 110.3 º]. Symmetry operations used (') x-1, y, z, ('') -x, -y+1, -z+1 and (''') -x, -y+2, -z+1. Red dotted lines (between donor and acceptor atoms) represent hydrogen bonds.

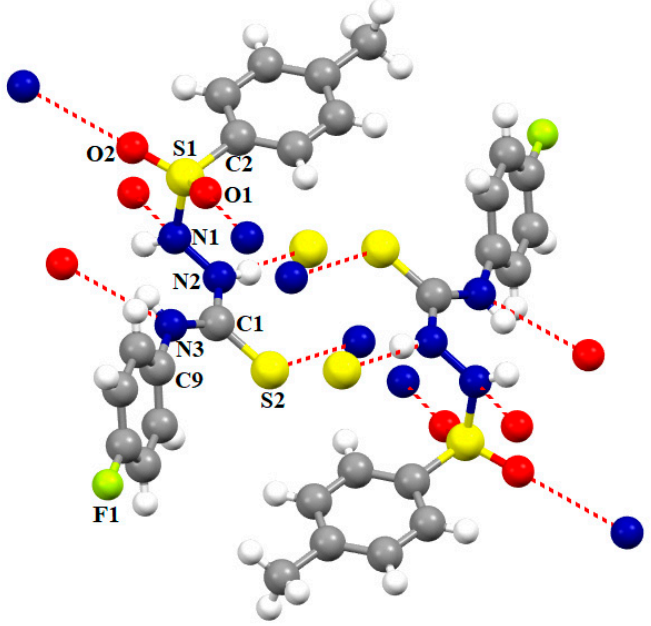


**Figure S4.2 -** Hydrogen bonds involved in the crystalline structure of the **HL5**$^{\mathbf{NO2Ph}}$ ligand. Intermolecular hydrogen bonds: [N(1)···O(1') = 2.887(4) Å, N(1)-H(1)···O(1')= 117.4º], [N(2)···S(1'') = 3.347(3) Å, N(2)-H(2)···S(1'') = 165.2º], [N(3) ···O(2''') = 3.040(4) Å, N(3)-H(3)···O(2''') = 151.8º]. Symmetry operations used (') x-1,y,z, ('') -x, -y+1, -z+1 and (''') -x,-y,-z+1. Blue dotted lines (between donor and acceptor atoms) represent hydrogen bonds.

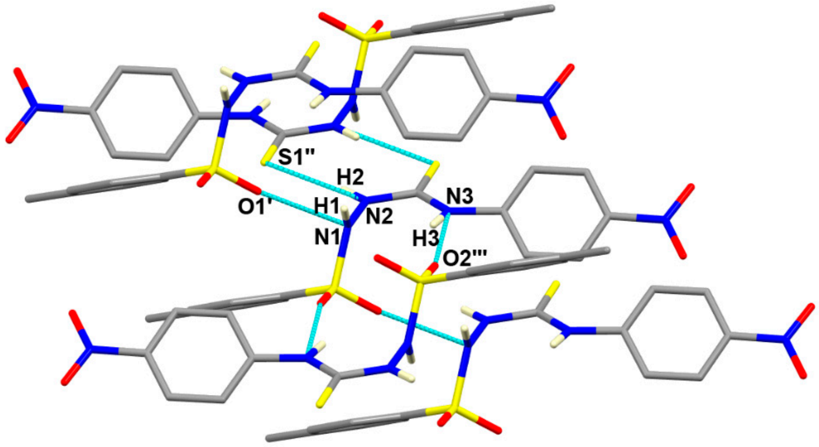

**Figure S4.31 -** Hydrogen bonds involved in the crystalline structure of the **HL6$^{Al}$** ligand. Intermolecular hydrogen bonds: [N(3)···O(2) = 3.024(3) Å, N(3)-H(3)···O(2)= 129.0º], [N(2)···S(1) = 3.3475(18) Å, N(2)-H(2)···S(1) = 159.0º], [N(1)···S(1) = 3.356(2) Å, N(1)-H(1)···S(1)= 118.4º]. Symmetry operations used (') -x, -y, -z+1, ('') -x+1, -y+1, -z+1 and (''') x-1, y, z. Blue dotted lines (between donor and acceptor atoms) represent hydrogen bonds.

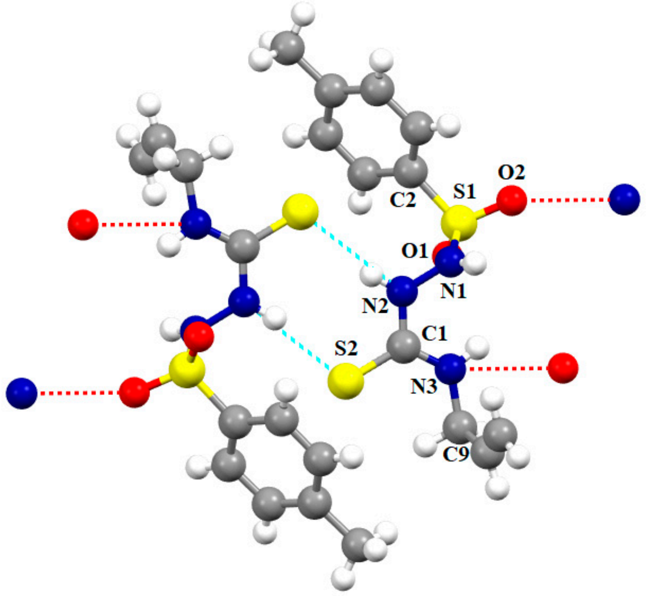


**Figure S4.4** Ellipsoid representation of [Au(HL1$^{Ch}$)$_2$]Cl·$CH_3OH$ (**Au1**·$CH_3OH$). The hydrogen atoms have been omitted for clarity. A solvent mask was calculated and 108 electrons were found in a volume of 518 Å$^3$ in 1 void per unit cell. This is consistent with the presence of 0.5[$CH_2Cl_2$; $2H_2O$] per Asymmetric Unit which account for 120 electrons per unit cell.

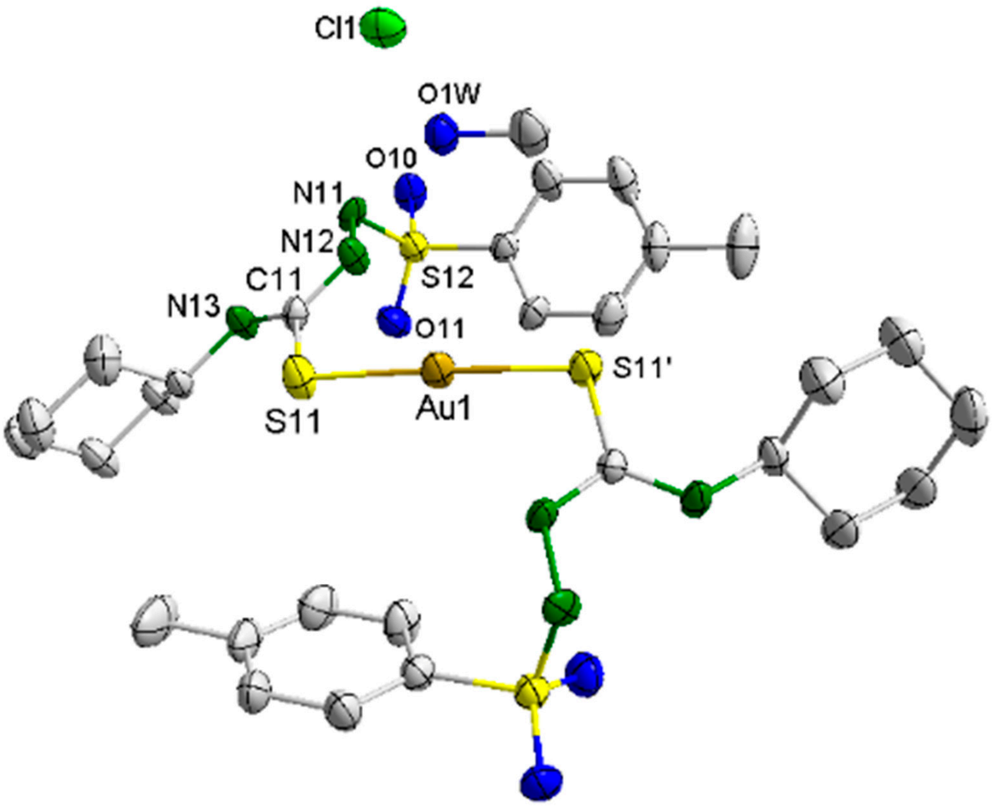

**Figure S4.5** Ellipsoid representation of $[Au(HL2^{Ph})_2]Cl{\cdot}CH_3OH$ (**Au2**$\cdot CH_3OH$). The hydrogen atoms have been omitted for clarity.

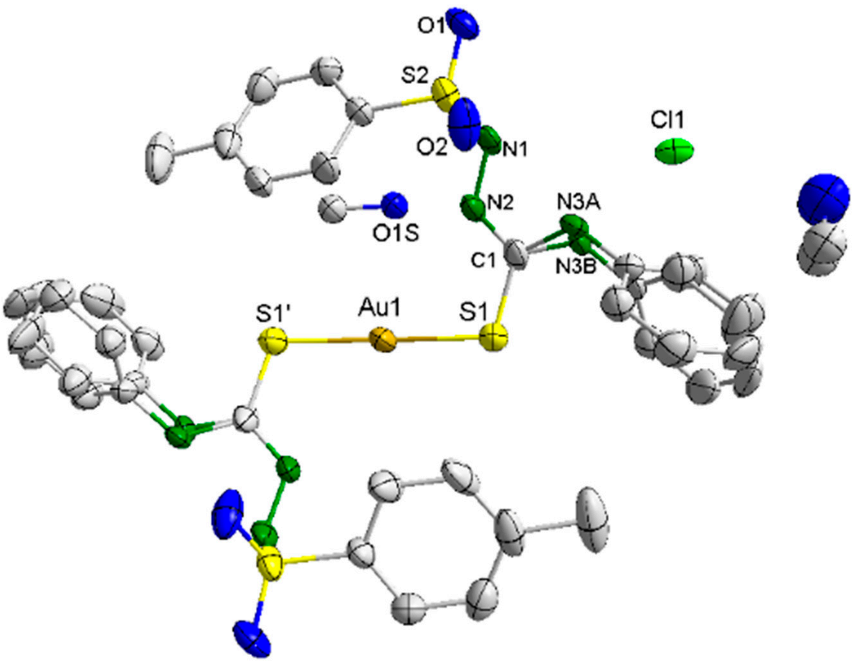


**Figure S4.6 - -** Hydrogen bonds involved in the crystalline structure of the **Au2** complex. Intermolecular hydrogen bonds: [N(1)···Cl(1) = 3.173(4) Å, N(1)-H(1)···Cl(1)= 113.8º], [O(1S)···Cl(1) = 3.135(5) Å, O(1S)-H(1S)···Cl(1) = 152.4º], [N(3A)···O(1) = 2.775(14) Å, N(3A)-H(3A)···O(1) = 146.1º], [N(3B)···O(1) = 3.149(14) Å, N(3B)-H(3B)···O(1) = 136.9º]. Intramolecular hydrogen bonding: [N(2)···O(1S) = 2.905(5) Å, N(2)-H(2)···O(1S)= 135.6 º]. Symmetry operations used (') -x, +y, -z. Blue dotted lines (between donor and acceptor atoms) represent hydrogen bonds.

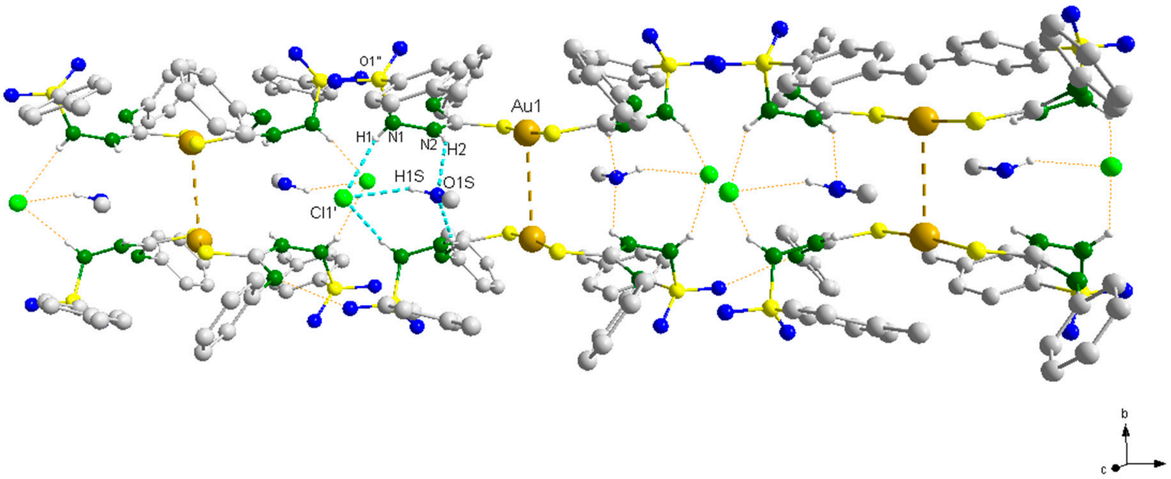

**Figure S5.1 -** Electronic spectra of **Ag3** (green line), **Ag4** (orange line), **Ag5** (red line) and **Ag6** (purple line) in the 210 to 350 nm range ($10^{-6}$ M $CH_3CN$ solution).

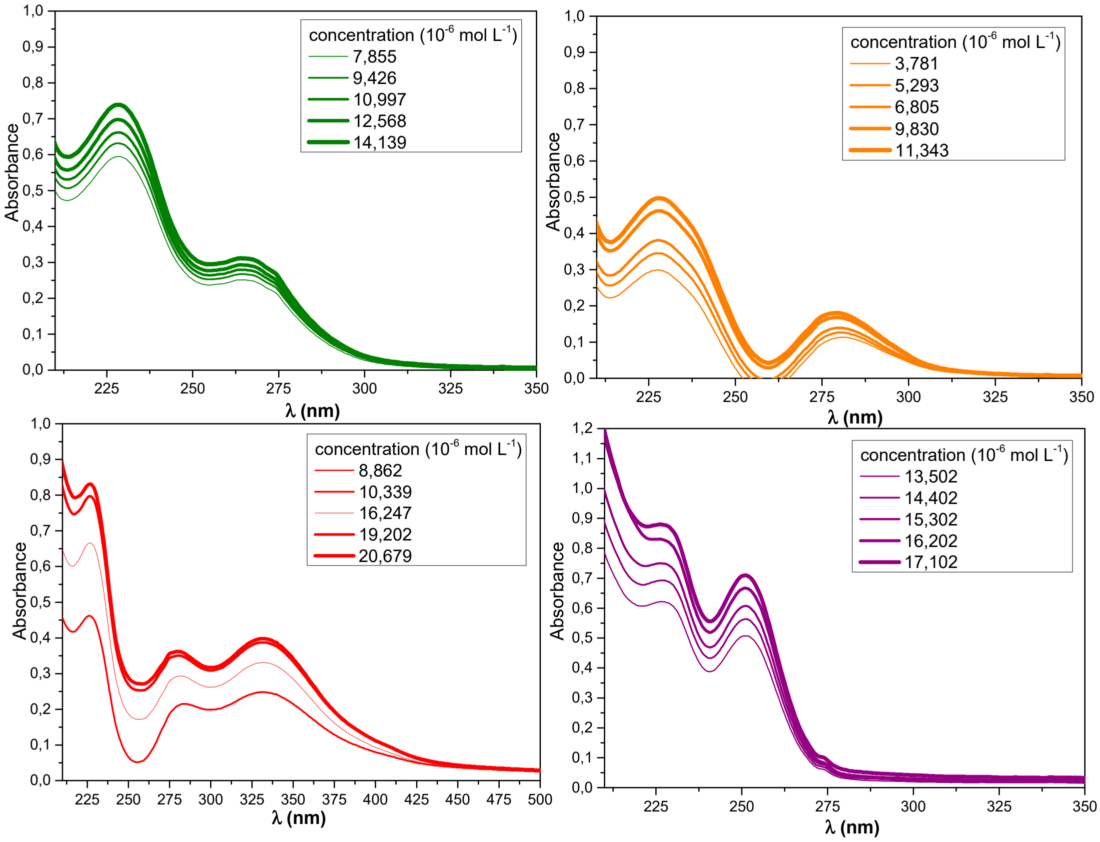

**Figure S5.2 –** Electronic spectra of **Au1** (blue line), **Au2** (pink line), **Au3** (green line), **Au4** (orange line), **Au5** (red line) and **Au6** (purple line) in the 210 to 350 nm range ($10^{-6}$ M $CH_3OH$ solution for **Au1**, **Au2**, **Au3**, **Au6** and $10^{-6}$ M solution $CH_3CN$ for **Au4** and **Au5**).

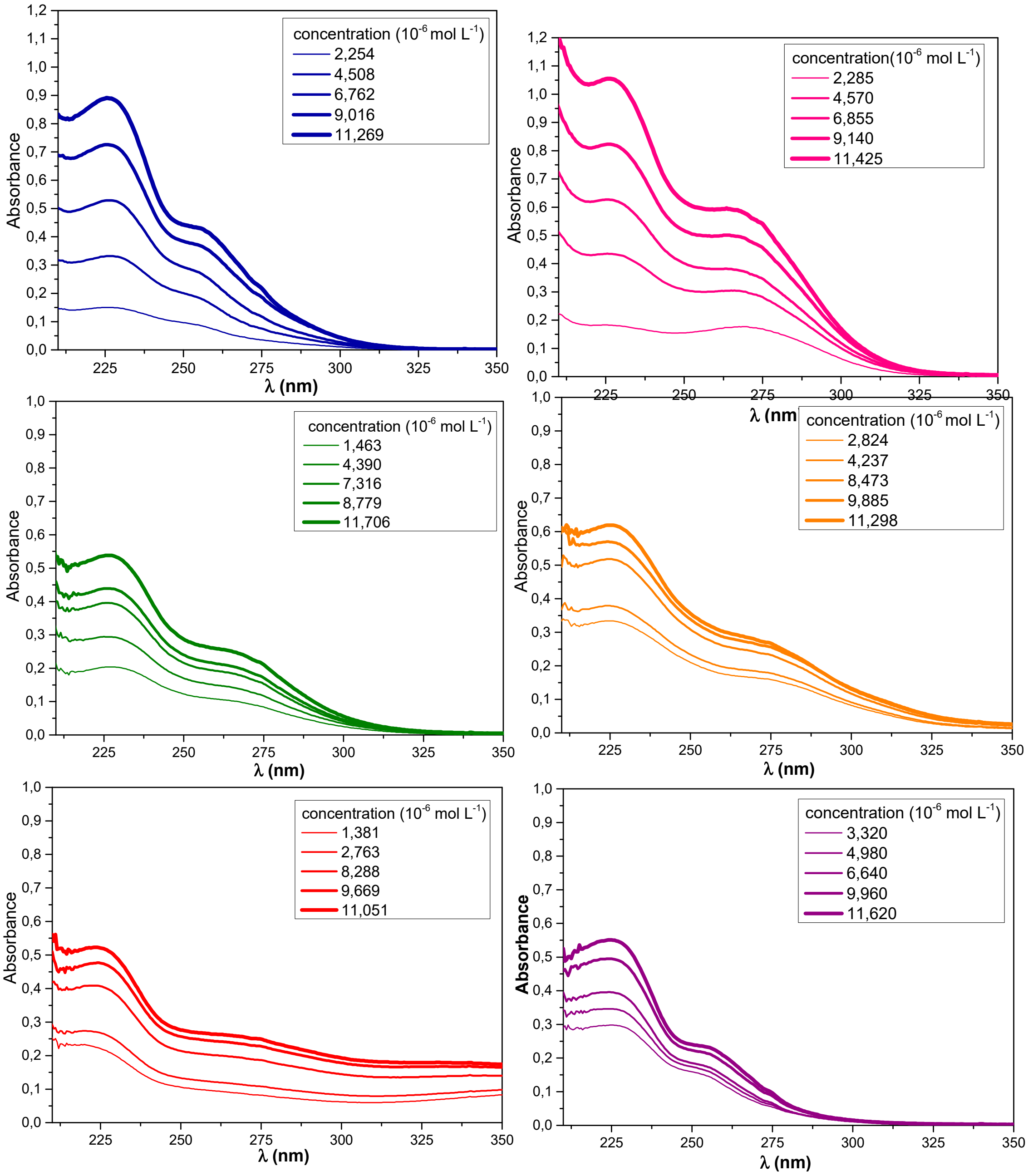

**Figure S5.32 –** Linear regression for **Ag3** (green line), **Ag4** (orange line), **Ag5** (red line) and **Ag6** (purple line).

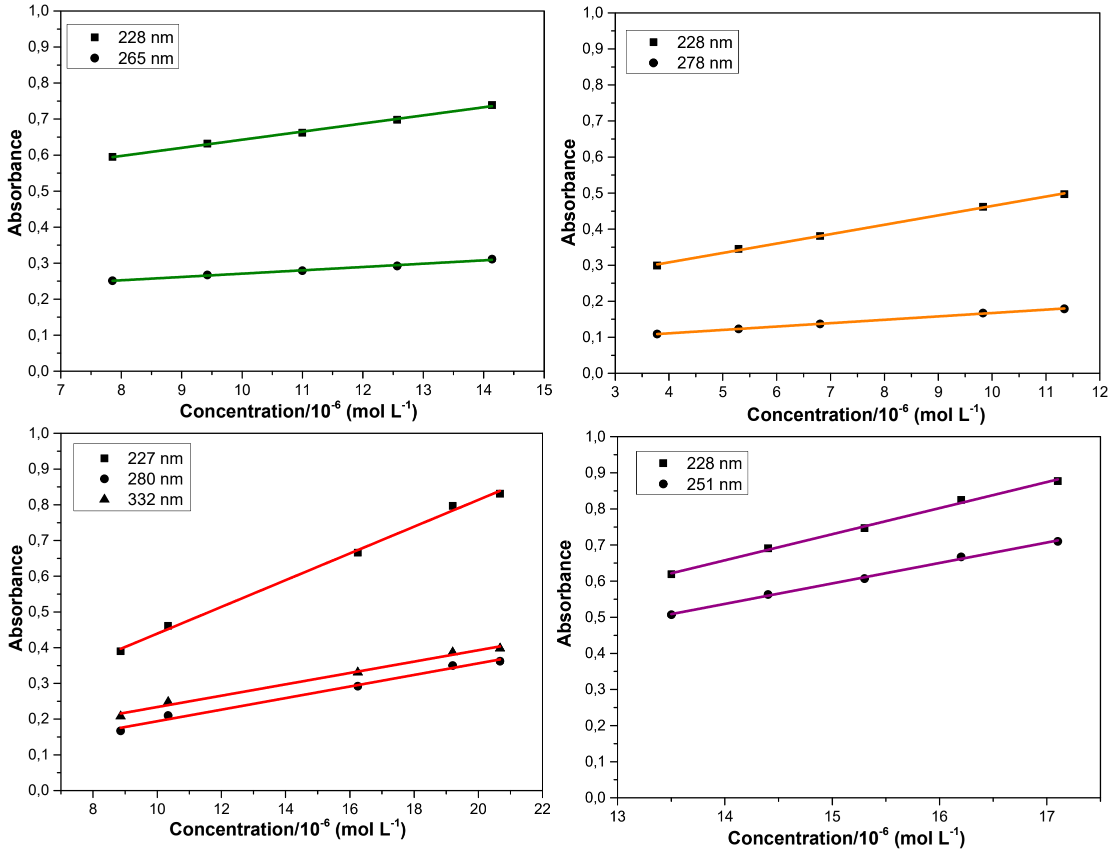


**Table S2 –** Electronic spectra data obtained for the **Ag3** and **Ag4** complexes.

| **Ag3 complex** | | | **Ag4 complex** | | |
|---|---|---|---|---|---|
| **Concentration ($10^{-6}$ mol L$^{-1}$)** | **$A_{228}$** | **$A_{265}$** | **Concentration ($10^{-6}$ mol L$^{-1}$)** | **$A_{228}$** | **$A_{278}$** |
| 7.855 | 0.595 | 0.251 | 3.781 | 0.299 | 0.109 |
| 9.426 | 0.632 | 0.267 | 5.293 | 0.345 | 0.123 |
| 10.997 | 0.662 | 0.279 | 6.805 | 0.381 | 0.137 |
| 12.568 | 0.698 | 0.292 | 9.830 | 0.462 | 0.167 |
| 14.139 | 0.739 | 0.311 | 11.343 | 0.497 | 0.179 |

**Table S3 -** Electronic spectra data obtained for the **Ag5** and **Ag6** complexes.

| **Ag5 complex** | | | | **Ag6 complex** | | |
|---|---|---|---|---|---|---|
| **Concentration ($10^{-6}$ mol L$^{-1}$)** | **$A_{227}$** | **$A_{265}$** | **$A_{332}$** | **Concentration ($10^{-6}$ mol L$^{-1}$)** | **$A_{228}$** | **$A_{251}$** |
| 8.862 | 0.390 | 0.167 | 0.208 | 13.502 | 0.619 | 0.507 |
| 10.339 | 0.461 | 0.210 | 0.248 | 14.402 | 0.691 | 0.563 |
| 16.247 | 0.666 | 0.292 | 0.331 | 15.302 | 0.747 | 0.607 |
| 19.202 | 0.797 | 0.350 | 0.387 | 16.202 | 0.825 | 0.667 |
| 20.679 | 0.831 | 0.362 | 0.398 | 17.102 | 0.877 | 0.71 |

**Figure S5.33–** Linear regression for **Au1** (blue line), **Au2** (pink line), **Au3** (green line), **Au4** (orange line), **Au5** (red line) and **Au6** (purple line).

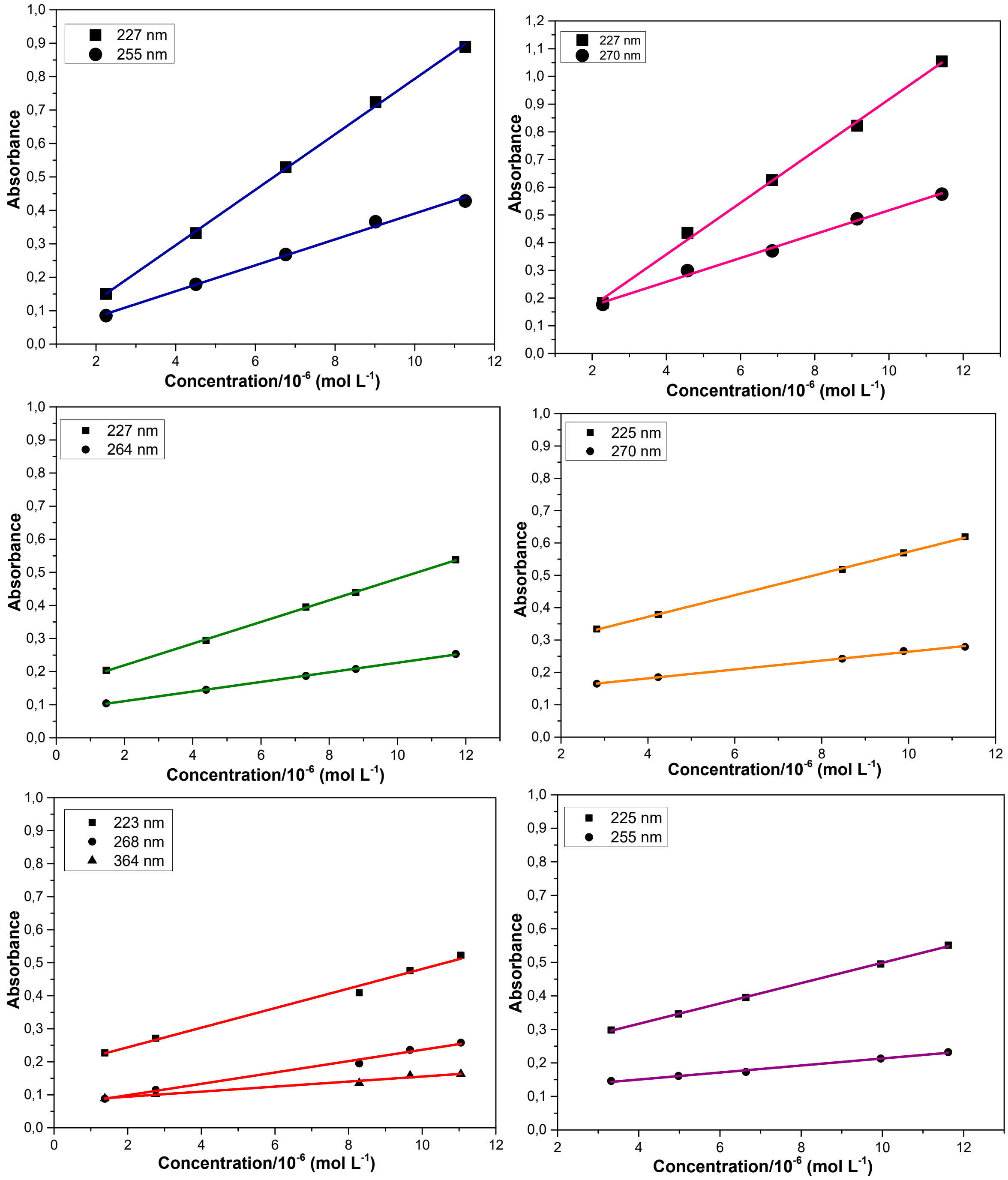


**Table S4 -** Electronic spectra data obtained for the **Au1** and **Au2** complexes.

| **Au1 complex** | | | **Au2 complex** | | |
|---|---|---|---|---|---|
| **Concentration ($10^{-6}$ mol L$^{-1}$)** | **$A_{227}$** | **$A_{255}$** | **Concentration ($10^{-6}$ mol L$^{-1}$)** | **$A_{227}$** | **$A_{270}$** |
| 2.254 | 0.150 | 0.085 | 2.285 | 0.182 | 0.177 |
| 4.508 | 0.332 | 0.179 | 4.570 | 0.435 | 0.299 |
| 6.762 | 0.529 | 0.268 | 6.855 | 0.626 | 0.370 |
| 9.016 | 0.724 | 0.366 | 9.140 | 0.822 | 0.486 |
| 11.269 | 0.889 | 0.428 | 11.425 | 1.054 | 0.575 |

**Table S5-** Electronic spectra data obtained for the **Au3** and **Au4** complexes.

| Au3 complex | | | Au4 complex | | |
|---|---|---|---|---|---|
| Concentration ($10^{-6}$ mol $L^{-1}$) | $A_{227}$ | $A_{264}$ | Concentration ($10^{-6}$ mol $L^{-1}$) | $A_{225}$ | $A_{270}$ |
| 1.463 | 0.204 | 0.104 | 2.824 | 0.334 | 0.165 |
| 4.390 | 0.294 | 0.145 | 4.237 | 0.379 | 0.185 |
| 7.316 | 0.395 | 0.187 | 8.473 | 0.518 | 0.242 |
| 8.779 | 0.439 | 0.208 | 9.885 | 0.569 | 0.266 |
| 11.706 | 0.538 | 0.253 | 11.298 | 0.619 | 0.279 |

**Table S6-** Electronic spectra data obtained for the **Au5** and **Au6** complexes.

| Au5 complex | | | | Au6 complex | | |
|---|---|---|---|---|---|---|
| Concentration ($10^{-6}$ mol $L^{-1}$) | $A_{223}$ | $A_{268}$ | $A_{364}$ | Concentration ($10^{-6}$ mol $L^{-1}$) | $A_{225}$ | $A_{255}$ |
| 1.381 | 0.227 | 0.088 | 0.089 | 3.320 | 0.298 | 0.146 |
| 2.763 | 0.271 | 0.115 | 0.102 | 4.980 | 0.346 | 0.161 |
| 8.288 | 0.409 | 0.195 | 0.136 | 6.640 | 0.395 | 0.173 |
| 9.669 | 0.476 | 0.236 | 0.158 | 9.960 | 0.495 | 0.213 |
| 11.051 | 0.523 | 0.258 | 0.163 | 11.620 | 0.551 | 0.232 |

**Figure S5.5** – (a) Emission spectra of **Au1** (blue line) and **Au2** (pink line) in $CH_3OH$ at 298 K; λexc = 255 nm for **Au1** and 270 nm for **Au2**; (b) Emission spectra of $CH_3OH$ at 298 K; λexc = 250 nm.

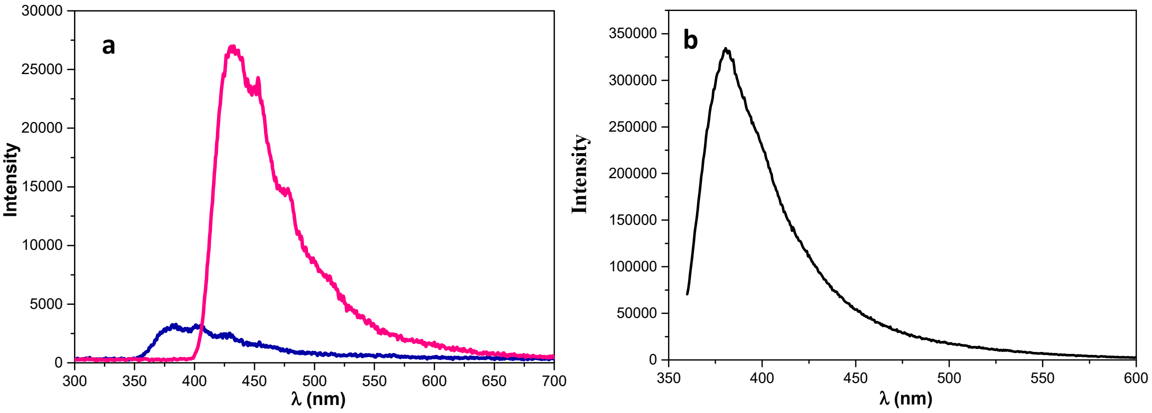


**Figure S5.6** – Emission spectra of **Au1** (blue dashed line) and **Au2** (pink dashed line) in MeOH at 77 K; λexc = 255 nm for **Au1** and 270 nm for **Au2**.

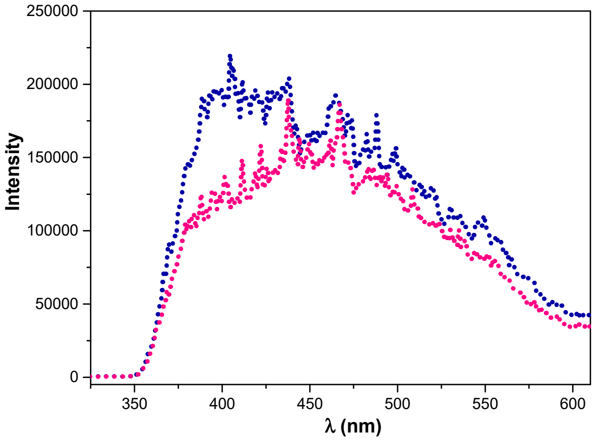

**Table S7.** Basis set functions used for gold complexes.

```
NewGTO H
S   3
1       12.98511484            0.0305015
2        1.95908134            0.2062392
3        0.44403649            0.7632592
S   1
1        0.11282695            1.0000000
P   1
1        0.80305095            1.0000000
end

NewGTO C
S   5
1     4949.46032594            0.0021254
2      758.82582124            0.0138797
3      172.62336223            0.0699682
4       47.77742113            0.2630053
5       15.15140603            0.6510212
S   2
1        5.39400345            0.7300080
2        2.11083628            0.2699919
S   1
1        0.52460137            1.0000000
S   1
1        0.15964728            1.0000000
P   4
1       18.97219923            0.0156205
2        4.18721595            0.0966388
3        1.21388239            0.3264242
4        0.38698643            0.5613164
P   1
1        0.12406489            1.0000000
D   1
1        0.61467289            1.0000000
end

NewGTO N
S   5
1     6711.76398035            0.0022301
2     1029.56088507            0.0140080
3      234.62552896            0.0699756
4       65.08616322            0.2624838
5       20.68134371            0.6513023
S   2
1        7.35948620            0.7394324
2        2.86133058            0.2605675
S   1
1        0.75772555            1.0000000
S   1
1        0.22278085            1.0000000
P   4
1       26.95312900            0.0161302
2        6.01777874            0.1007562
3        1.76062640            0.3305209
4        0.56064983            0.5525926
P   1
1        0.17525619            1.0000000
D   1
```

```
1        0.89591019             1.0000000
end

NewGTO O
S   5
1     8897.19445619             0.0023229
2     1365.26538810             0.0140091
3      311.21828724             0.0694697
4       86.33353862             0.2611441
5       27.42841118             0.6530540
S   2
1        9.74740298             0.7418317
2        3.77109950             0.2581682
S   1
1        1.04455407             1.0000000
S   1
1        0.30382295             1.0000000
P   4
1       34.75998468             0.0172481
2        7.80757529             0.1079455
3        2.29096903             0.3383584
4        0.71677956             0.5364479
P   1
1        0.21323853             1.0000000
D   1
1        1.25011953             1.0000000
end

NewGTO S
S   5
1    88298.52965731             0.0037231
2    13783.75049251             0.0144966
3     3273.99080251             0.0569731
4      933.93904998             0.2171714
5      294.47682844             0.7076356
S   2
1        5.19930244             0.5076446
2        2.03425969             0.4923553
S   1
1      101.30556573             1.0000000
S   1
1       37.95800089             1.0000000
S   1
1       15.05040359             1.0000000
S   1
1        0.38772280             1.0000000
S   1
1        0.15157298             1.0000000
P   6
1      759.75242296             0.0015116
2      180.00565026             0.0113965
3       56.73558346             0.0560869
4       20.36270362             0.1849910
5        7.76216777             0.3713178
6        3.02062956             0.3746960
P   1
1        1.11706212            -1.0000000
P   1
1        0.41415858             1.0000000
P   1
```

1 0.12911675 1.0000000
D 1
1 0.47158905 1.0000000
end

NewGTO Au
S 8
1 14703222.86676416 0.0037526
2 2262824.67803200 0.0100581
3 541256.94037958 0.0214627
4 164442.91034176 0.0419279
5 59155.59429110 0.0745368
6 24111.28878337 0.1319630
7 10152.24890131 0.2484028
8 4170.67168425 0.4678957
S 3
1 243.52806941 -0.4833215
2 117.22573806 -0.4189882
3 53.38778177 -0.0976901
S 2
1 1707.43394310 0.5704913
2 708.28135147 0.4295086
S 2
1 10.72476847 0.5023106
2 4.98291235 0.4976893
S 2
1 1.37574165 -0.7187708
2 0.66299967 -0.2812291
S 1
1 25.23167009 1.0000000
S 1
1 0.11066351 1.0000000
S 1
1 0.03911478 1.0000000
P 8
1 57867.45693148 0.0025583
2 14466.07282046 0.0069902
3 5096.80289128 0.0204152
4 2035.23445681 0.0610881
5 842.36068729 0.1629640
6 361.46395991 0.3385020
7 161.78689265 0.3128406
8 73.04451373 0.0946414
P 3
1 16.95126547 0.2800147
2 7.89562180 0.4069449
3 3.73415826 0.3130403
P 1
1 35.18821179 -1.0000000
P 1
1 1.40958119 1.0000000
P 1
1 0.52307734 1.0000000
P 1
1 0.12434867 1.0000000
P 1
1 0.03469933 1.0000000
D 5
1 1057.31409955 0.0149745
2 315.34985277 0.0904567

3 118.76210977 0.2923335
4 49.52241329 0.4170518
5 21.54470227 0.1851834
D 2
1 9.19880872 -0.7782852
2 3.88284809 -0.2217147
D 1
1 1.23471691 1.0000000
D 1
1 0.35702456 1.0000000
F 4
1 86.31773515 0.0583691
2 27.55700975 0.2444140
3 9.83940823 0.4153993
4 3.31580164 0.2818174
F 1
1 0.84103480 1.0000000

**Table S8 -** Vibrational modes and their theoretical assignments in related to the C=S bond.

| Compound | Vibrational frequency / $cm^{-1}$ | Vibrational mode assignment |
|---|---|---|
| $Au(HL1^{Ch})_2Cl$ | 790.64 / 794.59 | $\nu C{=}S + \delta_{oop}CH_{2(ciclohexane)}$ |
| $Au(HL2^{Ph})_2Cl$ | 744.73 / 746.69 | $\nu C{=}S + \delta NCN + \delta_{oop}CH_{2(ph)}$ |
| $Au(HL3^{FPh})_2Cl$ | 738.89 / 741.70 | $\nu C{=}S + \delta NCN + \delta_{oop}CH_{2(ph)}$ |
| $Au(HL4^{ClPh})_2Cl$ | 767.65 / 768.30 | $\nu C{=}S + \delta NCN + \delta_{oop}CH_{2(ph)}$ |
| $Au(HL5^{NO2Ph})_2Cl$ | 755.56 / 756.37 | $\nu C{=}S + \delta NCN + \delta_{oop}CH_{2(ph)}$ |
| $Au(HL6^{Al})_2Cl$ | 782.41 / 798.73 | $\nu C{=}S + \delta NCN + \delta_{roc\text{-}as}NH$ |

**Figure S6.1 -** Optimized structures of the silver complexes.

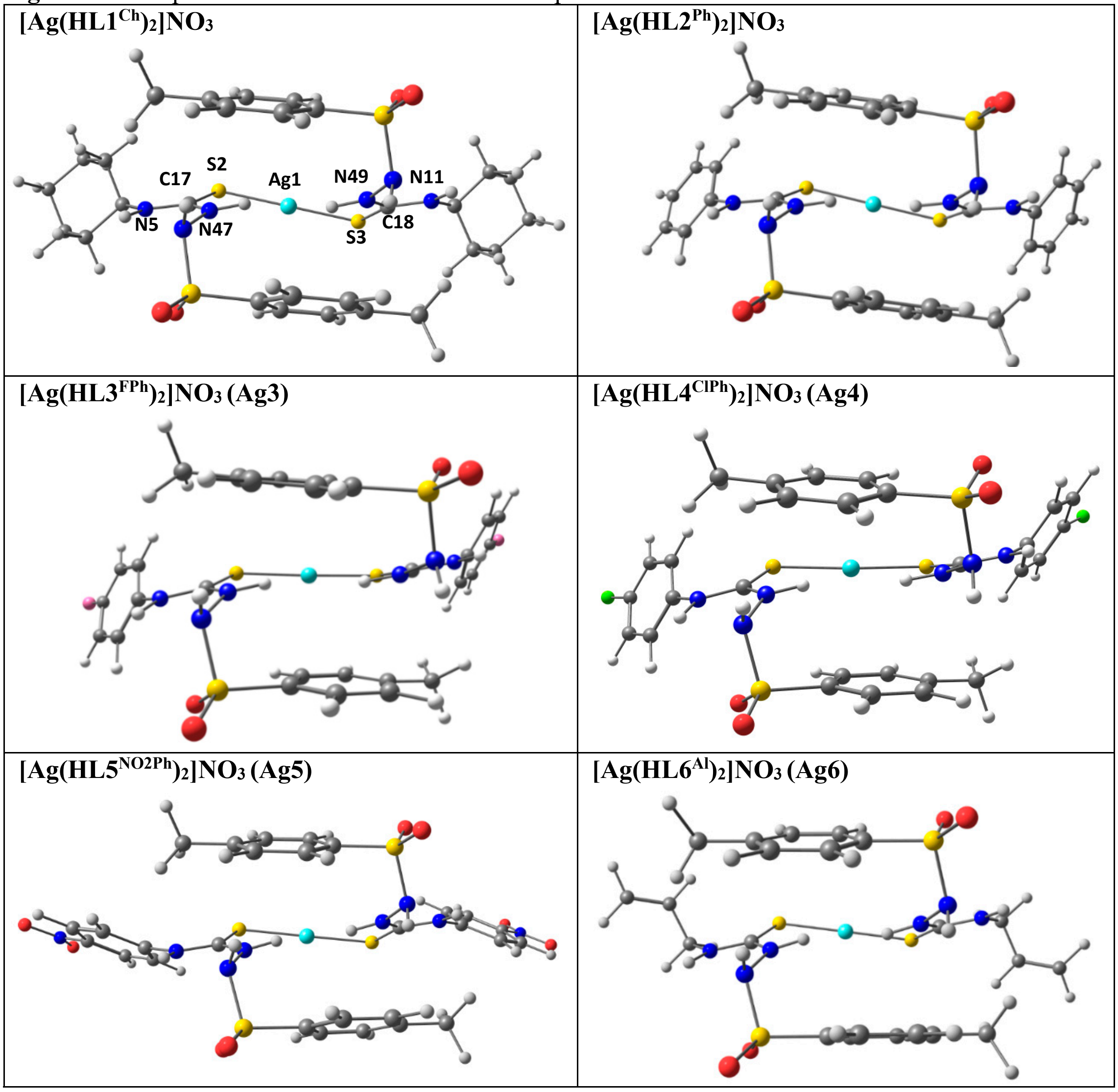


**Table S9 -** Main theoretical structural data of the silver complexes.

| | | Complexes | | | | | |
|---|---|---|---|---|---|---|---|
| | **Atoms Selected** | **Ag1** | **Ag2** | **Ag3** | **Ag4** | **Ag5** | **Ag6** |
| **Distances/ Å** | Ag1-S3 | 2.409 | 2.411 | 2.507 | 2.508 | 2.416 | 2.408 |
| | Ag1-S2 | 2.409 | 2.408 | 2.507 | 2.508 | 2.415 | 2.411 |
| **Angles/°** | S3-Ag1-S2 | 172.8 | 172.7 | 164.4 | 164.0 | 175.2 | 173.4 |
| **DIEDRAL /°** | Ag1-$S_2$-C17-$N_5$ | 17.4 | 14.2 | 11.8 | 11.7 | 13.7 | 16.8 |
| | Ag1-S3-C18-$N_{49}$ | -162.8 | -163.8 | -168.6 | -168.2 | -169.5 | -163.0 |
| | Ag1-S2-C17-N47 | -163.7 | -165.9 | -168.5 | -168.5 | -169.5 | -164.3 |
| | Ag1-S3-C18-$N_{11}$ | 18.3 | 16.5 | 11.8 | 12.1 | 13.7 | 18.1 |

**Figure S6.2 -** Optimized structures of the gold complexes.

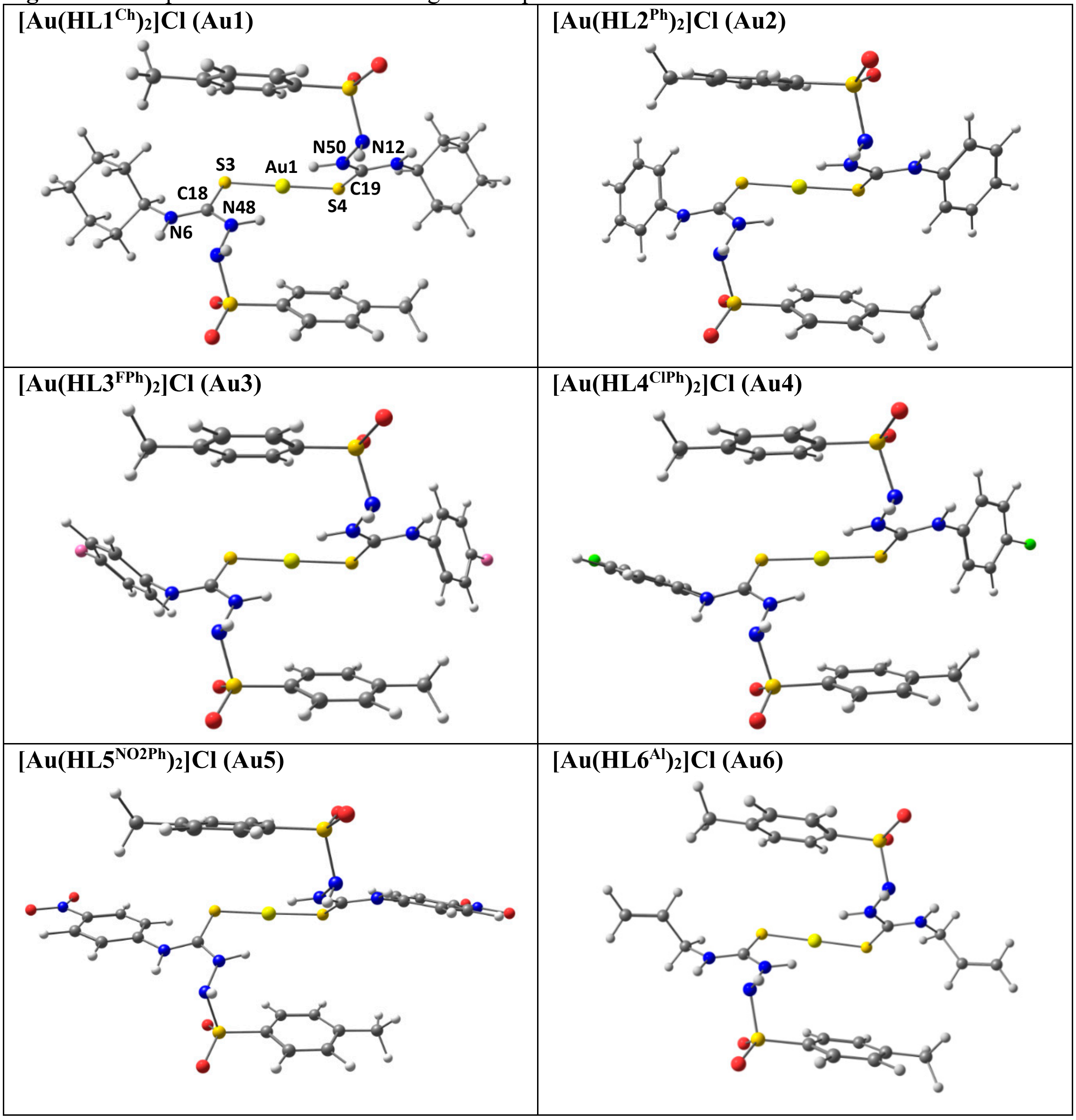


**Table S10 –** Main theoretical structural data of the gold complexes.

| | | Complexes | | | | | |
|---|---|---|---|---|---|---|---|
| | **Atoms Selected** | **Au1** | **Au2** | **Au3** | **Au4** | **Au5** | **Au6** |
| **Distances/ Å** | Au1-S4 | 2.284 | 2.289 | 2.287 | 2.287 | 2.286 | 2.284 |
| | Au1-S3 | 2.284 | 2.289 | 2.287 | 2.287 | 2.286 | 2.284 |
| **Angles/°** | S4-Au1-S3 | 174.0 | 175.0 | 174.3 | 175.0 | 175.1 | 174.8 |
| **DIEDRAL/°** | Au1-S4-C19-N12 | 11.1 | 15.3 | 9.2 | 6.9 | 6.2 | 8.4 |
| | Au1-S4-C19-N50 | -171.8 | -167.3 | -173.2 | -174.7 | -177.5 | -173.8 |
| | Au1-S3-C18-N48 | -173.4 | -176.0 | -176.0 | -178.1 | -178.2 | -174.3 |
| | Au1-S3-C18-N6 | 9.2 | 5.3 | 6.8 | 4.8 | 5.3 | 7.8 |

**Figure S6.3 -** HOMO and LUMO orbitals in the silver complexes.

| | |
|---|---|
| **$[Ag(HL1^{Ch})_2]NO_3$ (Ag1)** 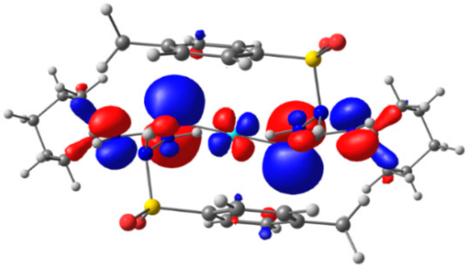 | 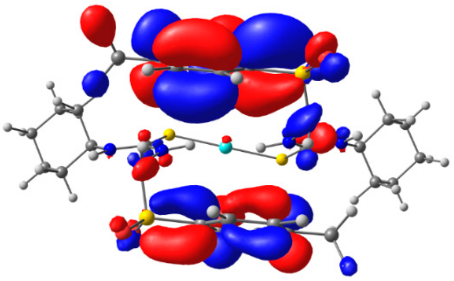 |
| **$[Ag(HL2^{Ph})_2]NO_3$ (Ag2)** 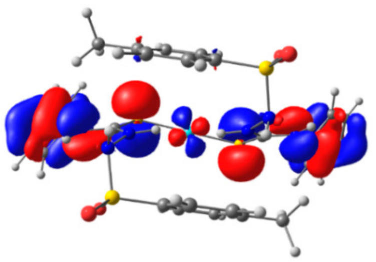 | 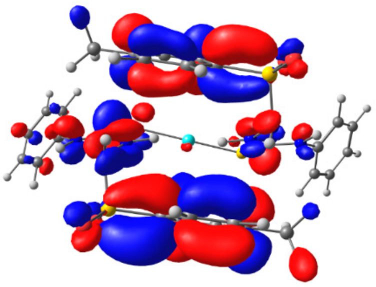 |
| **$[Ag(HL3^{FPh})_2]NO_3$ (Ag3)** 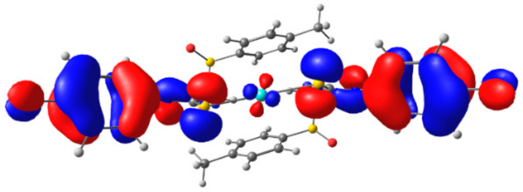 | 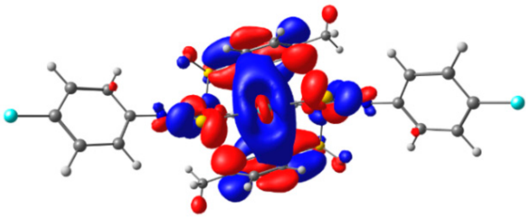 |
| **$[Ag(HL4^{ClPh})_2]NO_3$ (Ag4)** 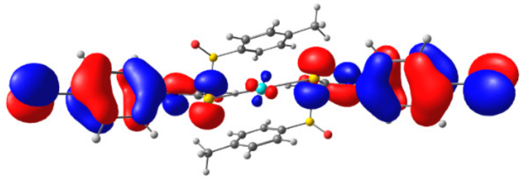 | 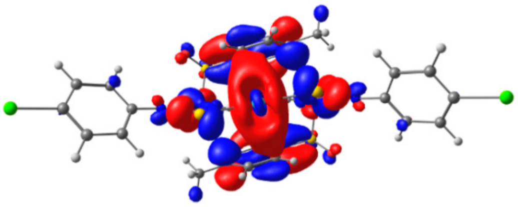 |
| **$[Ag(HL5^{NO2Ph})_2]NO_3$ (Ag5)** 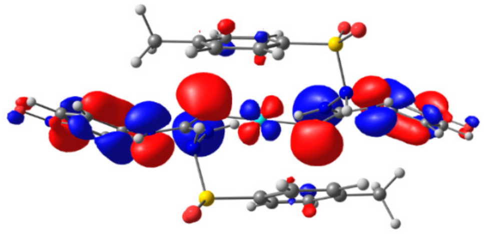 | 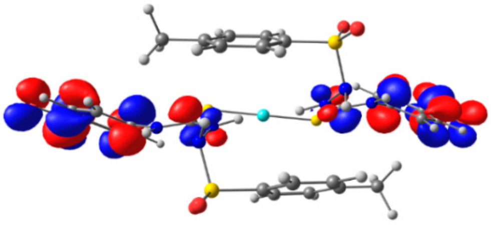 |
| **$[Ag(HL6^{Al})_2]NO_3$ (Ag6)** 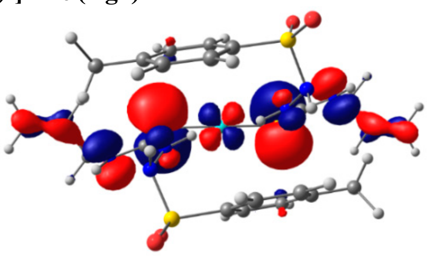 | 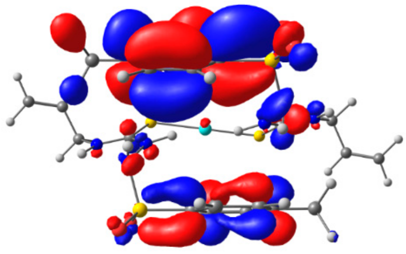 |

**Figure S6.4 -** HOMO and LUMO orbitals in the gold complexes.

| | |
|---|---|
| **[Au(HL1$^{Ch}$)$_2$]Cl (Au1)** 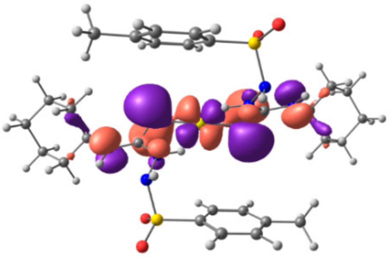 | 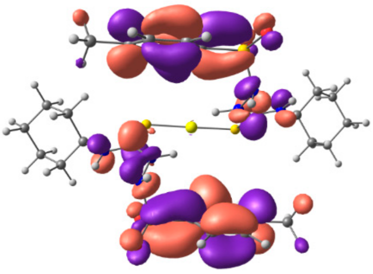 |
| **[Au(HL2$^{Ph}$)$_2$]Cl (Au2)** 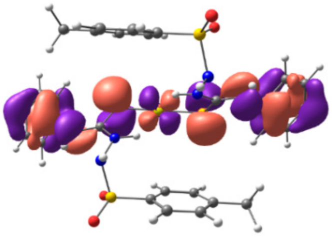 | 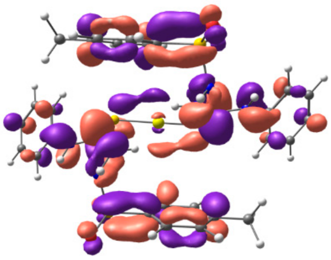 |
| **[Au(HL3$^{FPh}$)$_2$]Cl (Au3)** 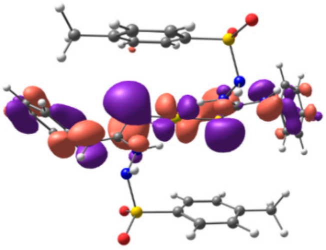 | 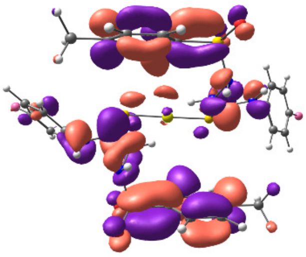 |
| **[Au(HL4$^{ClPh}$)$_2$]Cl (Au4)** 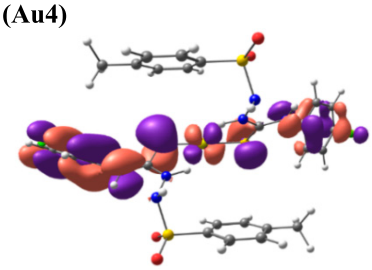 | 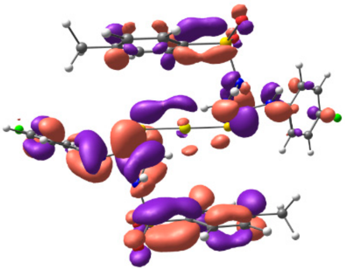 |
| **[Au(HL5$^{NO2Ph}$)$_2$]Cl (Au5)** 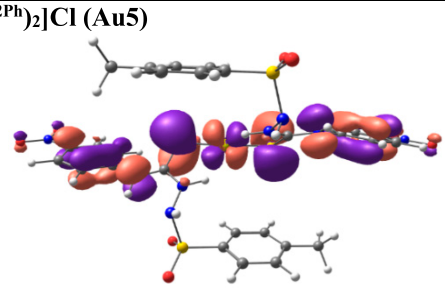 | 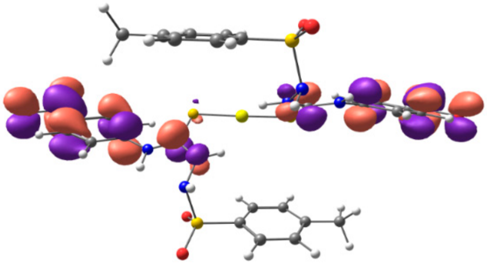 |
| **[Au(HL6$^{Al}$)$_2$]Cl (Au6)** 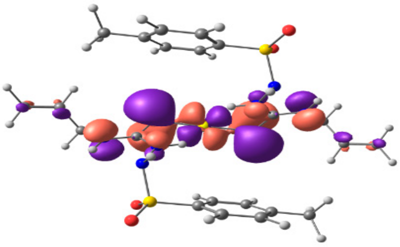 | 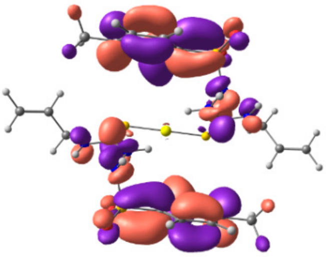 |

**Figure S6.5 –** Optimized structure of $[Au(HL3^{FPh})_2]^+$ (**Au3**) with the charges obtained for the AIM data.

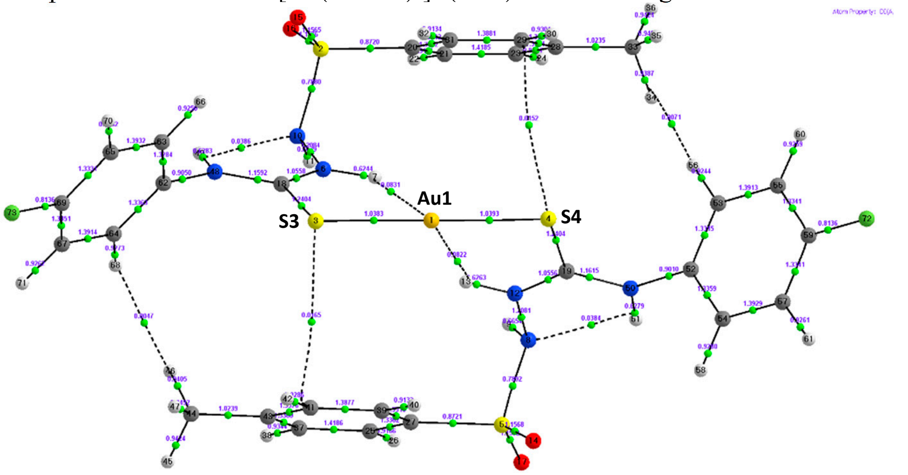


**Table S11 –** Electronic properties of the gold(I) complexes. The values are shown, respectively, for each bond in the order they appear.

| $[Au(HL1^{Ch})_2]Cl$ (**Au1**) | q | DI | $\rho$ | $\nabla^2\rho$ |
|---|---|---|---|---|
| Au | +0.1028 | | | |
| S3 | +0.0588 | | | |
| S4 | +0.0589 | | | |
| S3-Au-S4 | | 1.0459/1.0463 | +0.1114/+0.1115 | +0.1698/+0.1695 |
| S3-C18 | | 1.2225 | +0.1910 | -0.2597 |
| S4-C19 | | 1.2226 | +0.1910 | -0.2593 |
| $[Au(HL2^{Ph})_2]Cl$ (**Au2**) | q | DI | $\rho$ | $\nabla^2\rho$ |
| Au | +0.1229 | | | |
| S3 | +0.0710 | | | |
| S4 | +0.0661 | | | |
| S3-Au-S4 | | 1.0344/1.0396 | +0.1104/+0.1104 | +0.1704/+0.1705 |
| S3-C18 | | 1.2346 | +0.1909 | -0.2383 |
| S4-C19 | | 1.2355 | +0.1911 | -0.2403 |
| $[Au(HL3^{FPh})_2]Cl$ (**Au3**) | q | DI | $\rho$ | $\nabla^2\rho$ |
| Au | +0.1078 | | | |
| S3 | +0.0912 | | | |
| S4 | +0.0880 | | | |
| S3-Au-S4 | | 1.0383/1.0393 | +0.1111/+0.1111 | +0.1691/+0.1686 |
| S3-C18 | | 1.2404 | +0.1917 | -0.2292 |
| S4-C19 | | 1.2404 | +0.1917 | -0.2312 |
| $[Au(HL4^{ClPh})_2]Cl$ (**Au4**) | q | DI | $\rho$ | $\nabla^2\rho$ |
| Au | +0.1110 | | | |
| S3 | +0.0994 | | | |
| S4 | +0.0946 | | | |
| S3-Au-S4 | | 1.0350/1.0367 | +0.1110/+0.1110 | +0.1690/+0.1686 |
| S3-C18 | | 1.2446 | +0.1915 | -0.2251 |
| S4-C19 | | 1.2439 | +0.1916 | -0.2269 |
| $[Au(HL5^{NO2Ph})_2]Cl$ (**Au5**) | q | DI | $\rho$ | $\nabla^2\rho$ |
| Au | +0.1255 | | | |
| S3 | +0.1277 | | | |
| S4 | +0.1269 | | | |
| S3-Au-S4 | | 1.0285/1.0291 | +0.1112/+0.1112 | +0.1700/+0.1701 |
| S3-C18 | | 1.2611 | +0.1925 | -0.2081 |
| S4-C19 | | 1.2605 | +0.1925 | -0.2093 |
| $[Au(HL6^{Al})_2]Cl$ (**Au6**) | q | DI | $\rho$ | $\nabla^2\rho$ |
| Au | +0.1078 | | | |
| S3 | +0.0610 | | | |
| S4 | +0.0606 | | | |
| S3-Au-S4 | | 1.0432/1.0441 | +0.1113/+0.1114 | +0.1702/+0.1702 |
| S3-C18 | | 1.2238 | +0.1906 | -0.2545 |
| S4-C19 | | 1.2236 | +0.1907 | -0.2553 |

**Figure S7.1 –** Graph of the viability versus concentrations (μM) of the compounds.

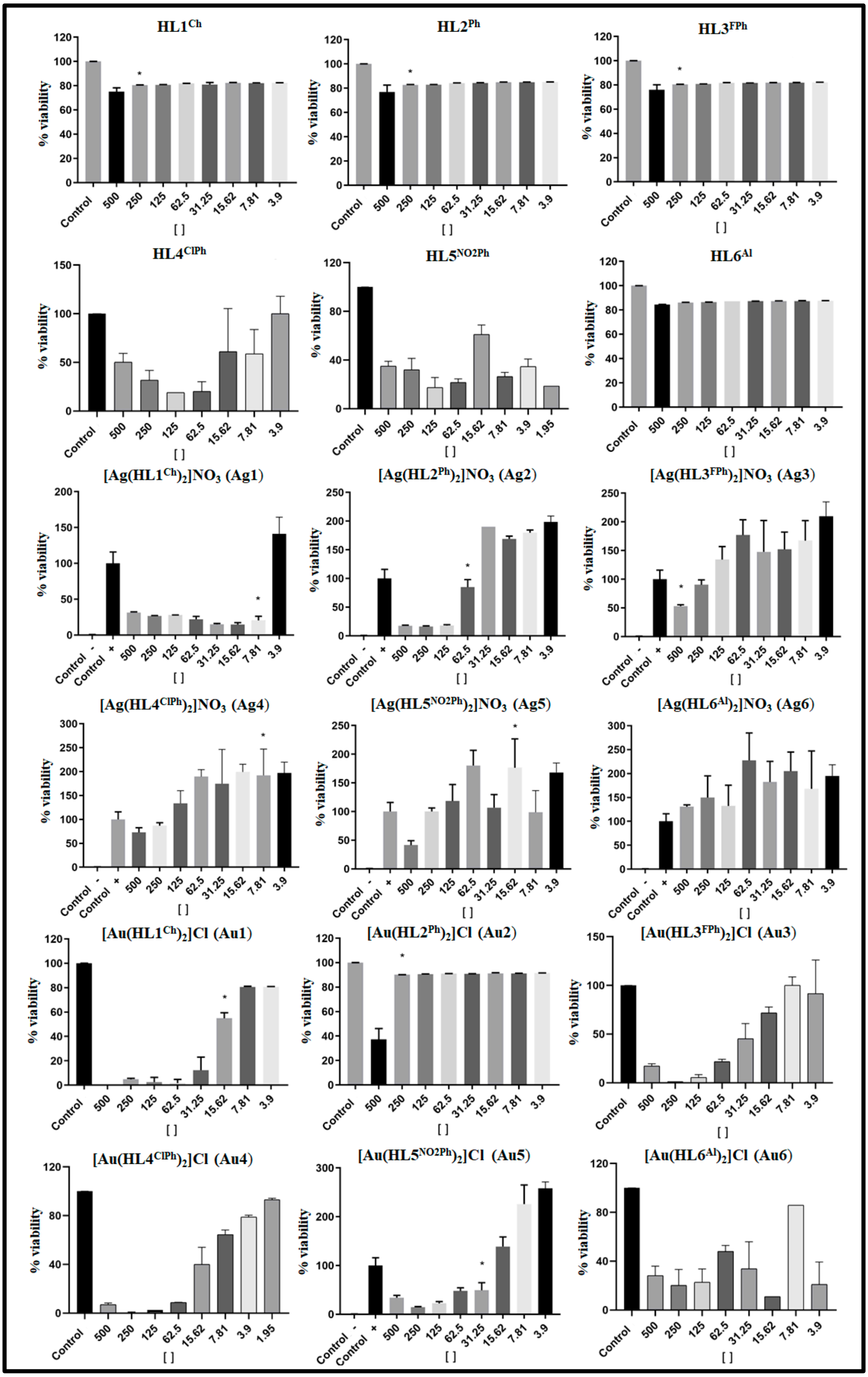

**Figure S7.2 –** Leishmanicidal activity against to the *Leishmania infantum* parasite by concentrations of the compound. Blue curve represents the reference leishmanicidal drug, Glucantime. Black curve represents each compound.

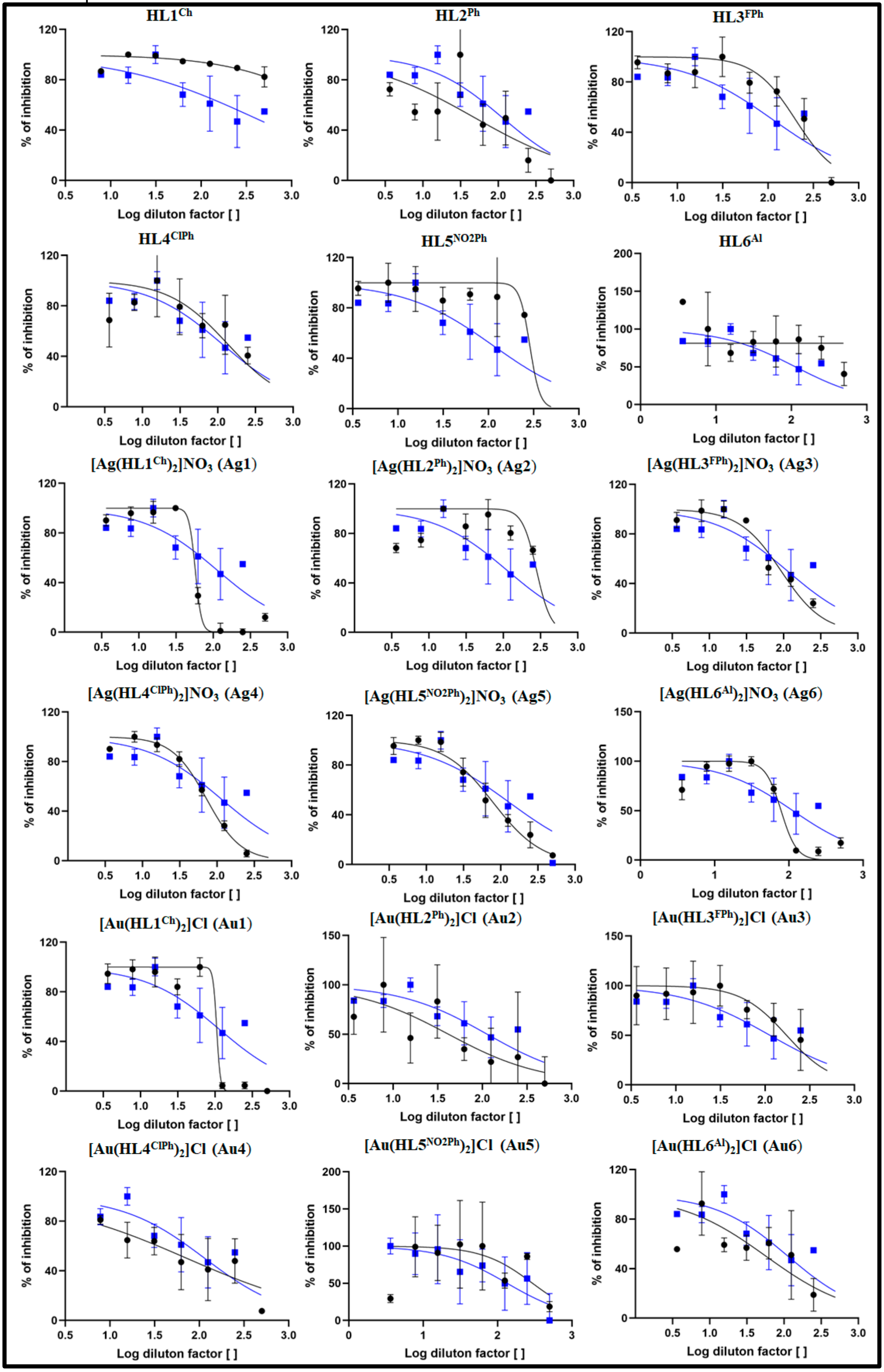

**Figure S7.3 –** Leishmanicidal activity against to the *Leishmania braziliensis* parasite by concentrations of the compound. Blue curve represents the reference leishmanicidal drug, Glucantime. Black curve represents each compound.

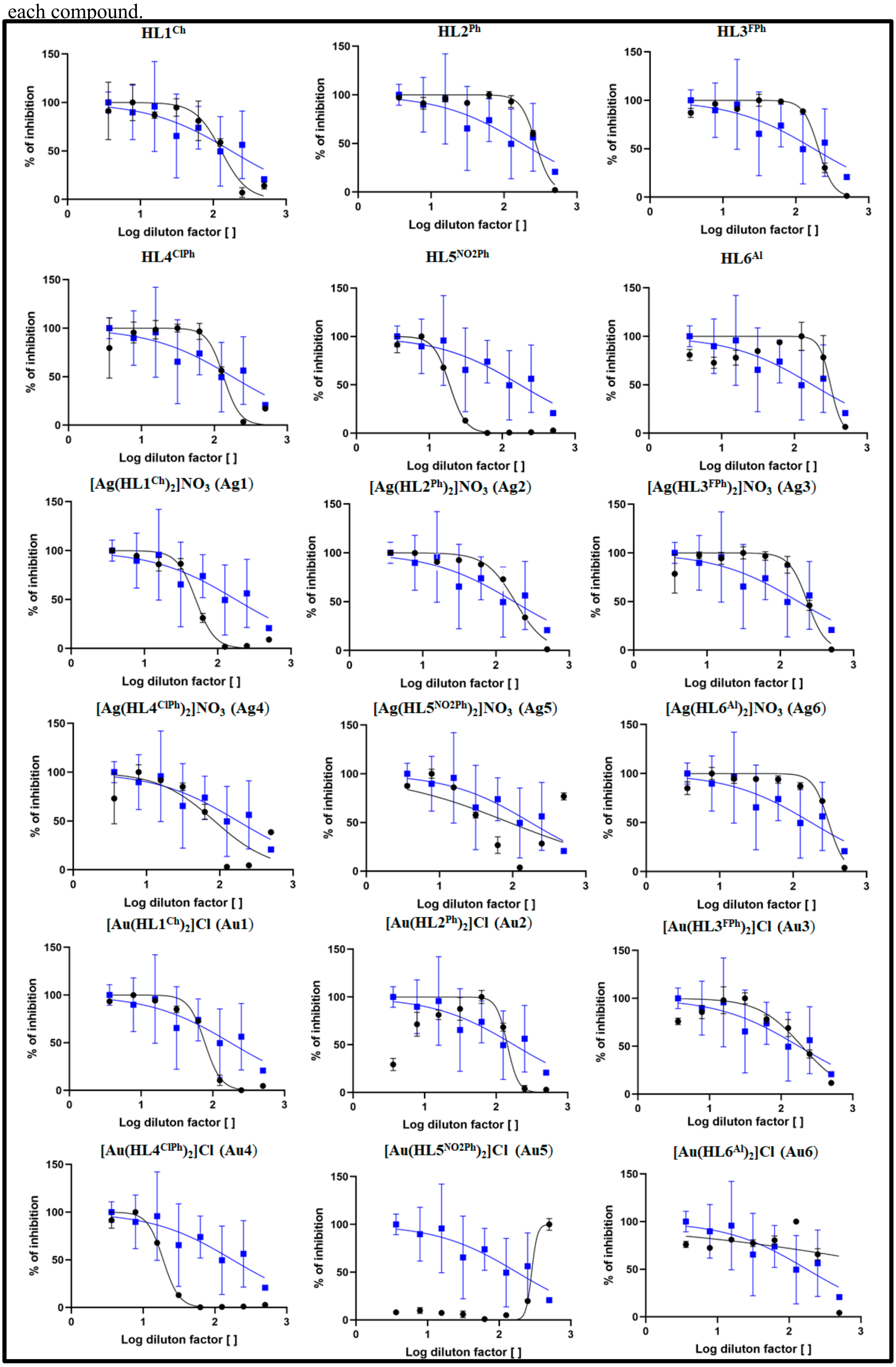

**Figure S7.4 –** (a) percentage of the number of infected in 200 macrophages by *Leishmania infantum*; (b) percentage of leishmaniasis that infect 100 cells/macrophages; (c) average percentage of leishmaniasis that infect 200 cells/macrophages; of the selected compounds.

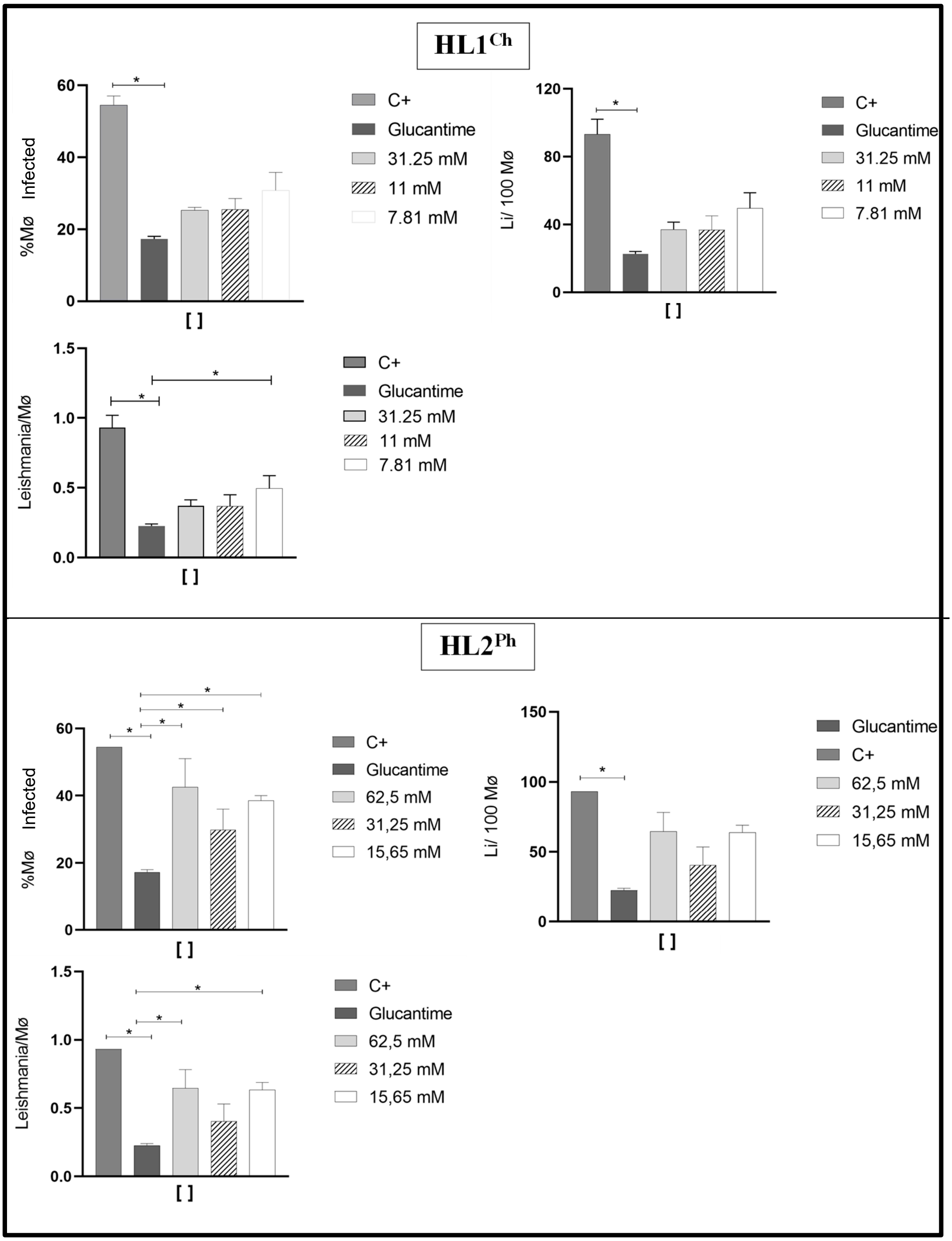

**$[Ag(HL3^{FPh})_2]NO_3$ (Ag3)**

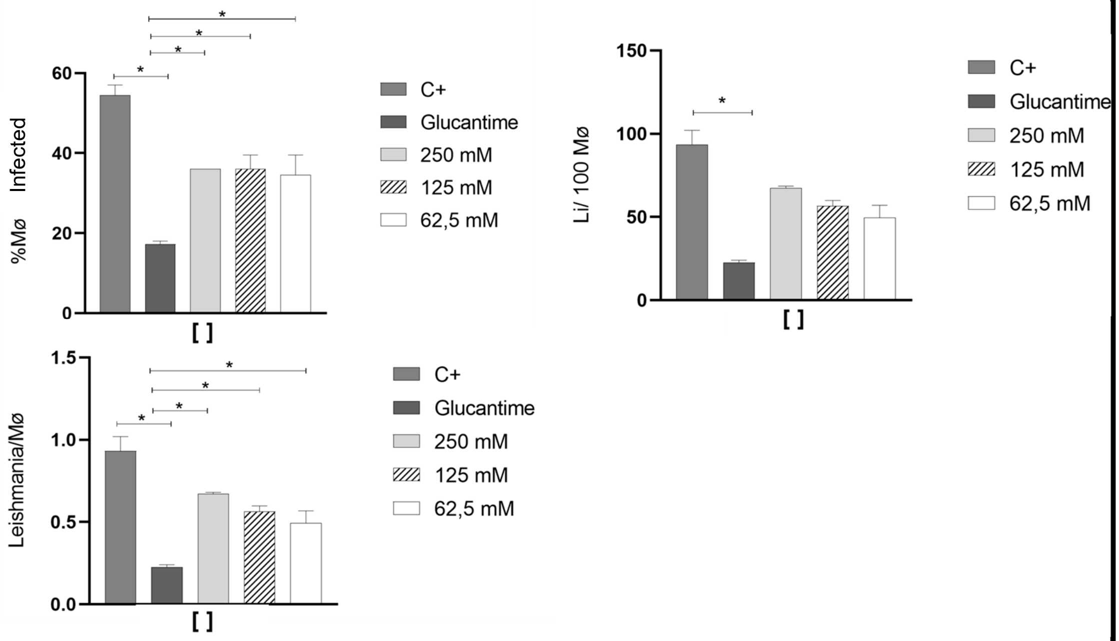


**$[Ag(HL4^{ClPh})_2]NO_3$ (Ag4)**

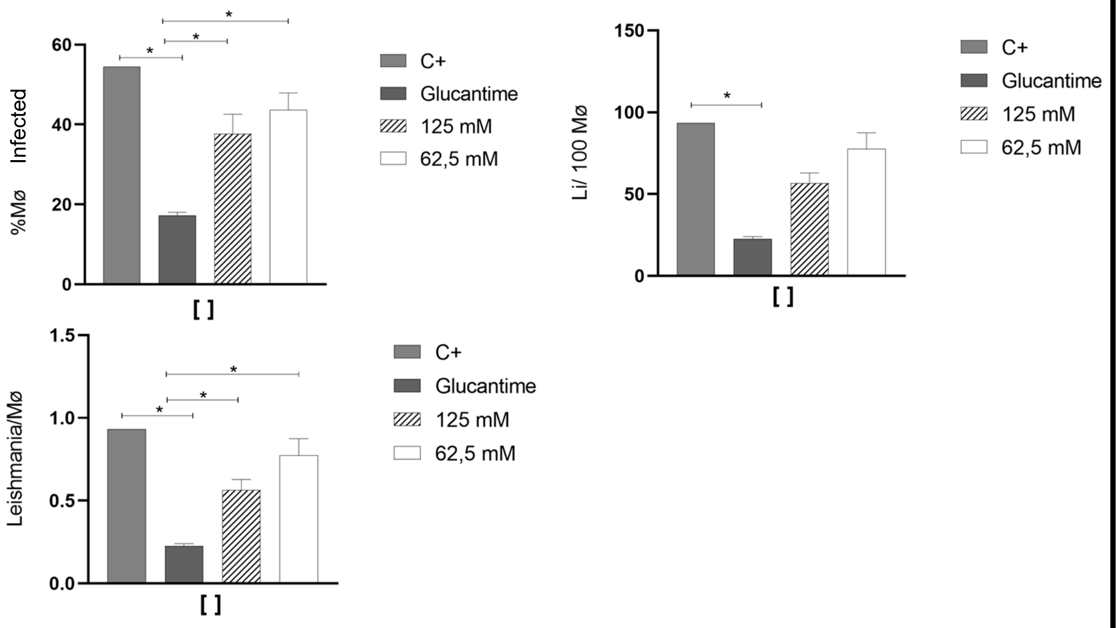

**[Ag(HL6$^{Al}$)$_2$]NO$_3$ (Ag6)**

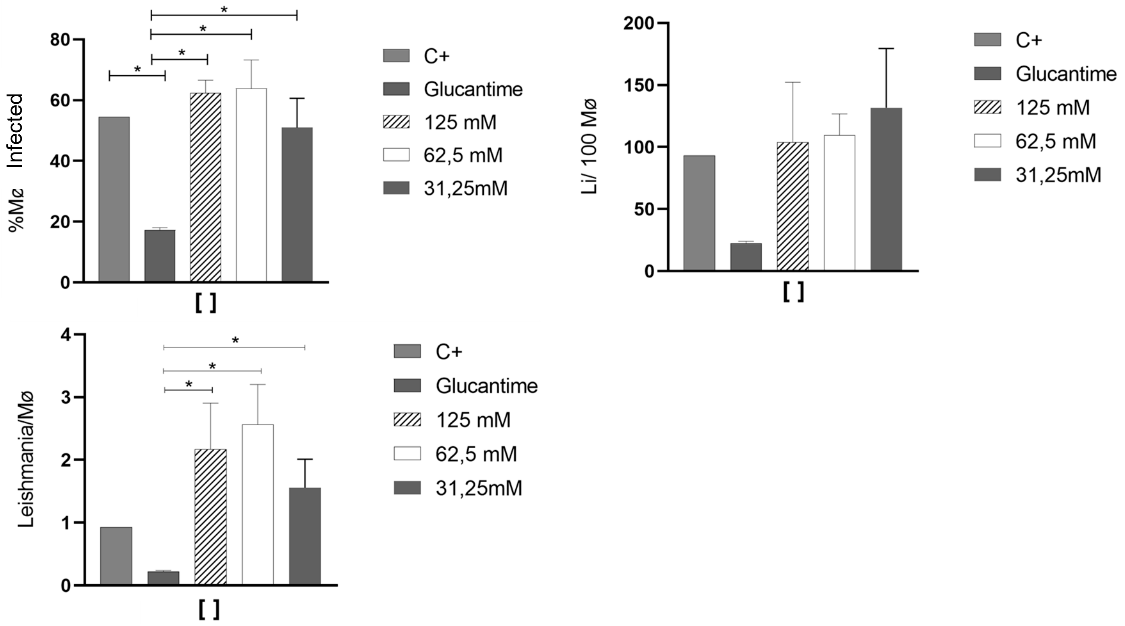


**[Au(HL2$^{Ph}$)$_2$]NO$_3$ (Au2)**

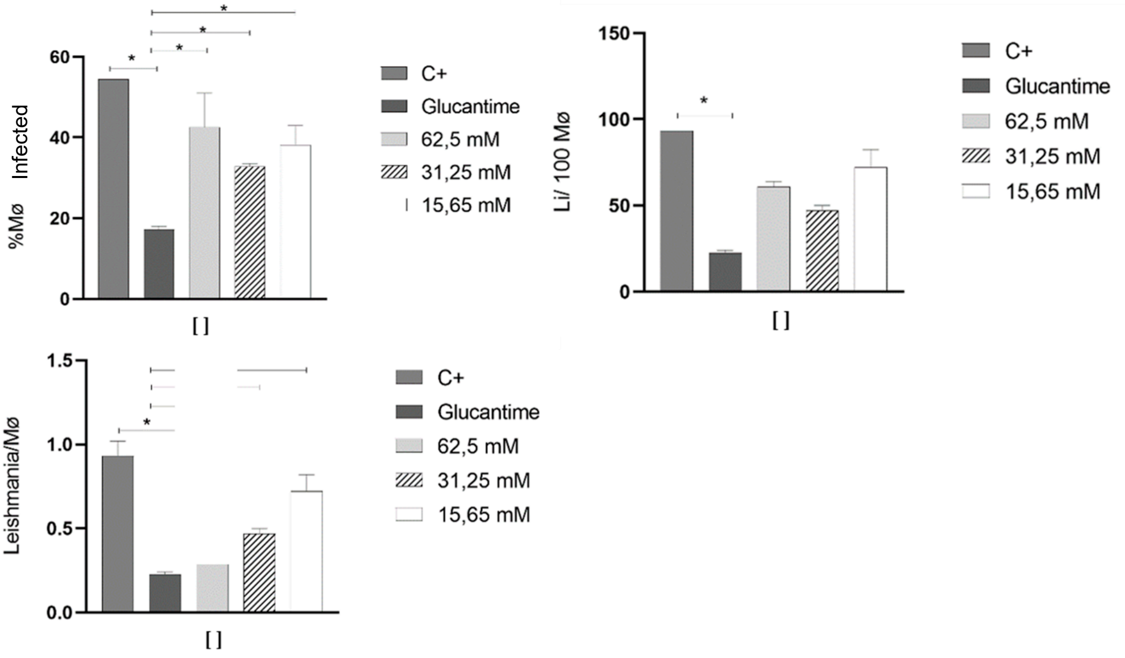

**Figure S7.5 -** Image captured under a fluorescence microscope (EVOS) of Raw macrophages infected by *Leishmania infantum* after treatment with a Glucantime (leishmanicidal drug) (A) and the selected compounds;

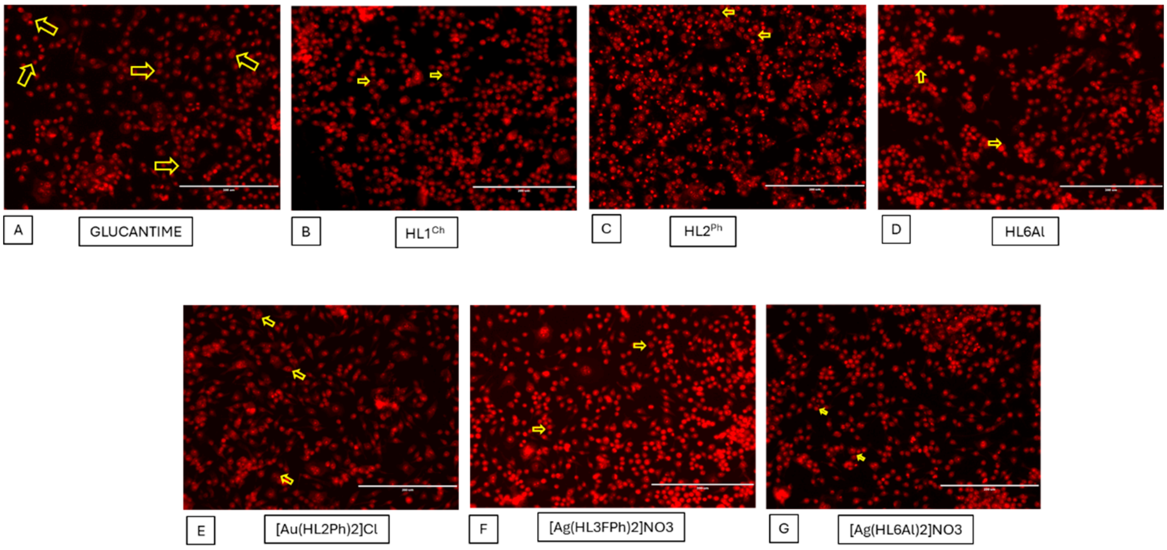

**Figure S7.6 –** (**A**) Fluorescence emission spectra ($\lambda_{ex}$ = 295 nm) of LbOYE protein in the concentration of 1 µmol $L^{-1}$ in the presence of increasing concentrations of the $HL5^{NO2Ph}$ compound. (**B**) Variation of maximum fluorescence intensities ($\lambda_{max}$ = 333 nm) and non-linear fit to the Hill equation (Equation 1). (**C**) Logarithmic relationship to obtain the number of bonding sites according to Equation 2. (**D**) Comparison between the temperatures of 25 and 37 °C. The spectra were obtained in Tris-HCl buffer, pH 8.0; 100 mmol $L^{-1}$ of NaCl and 2.5 % of DMSO.

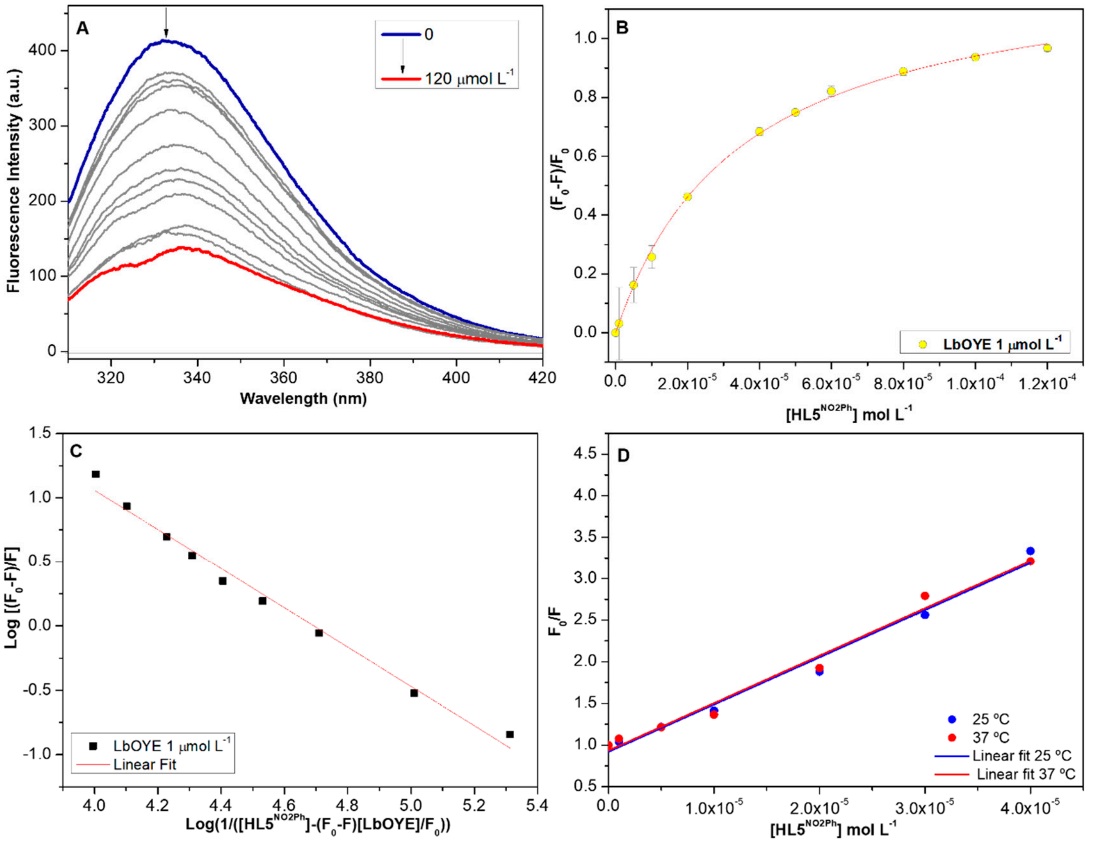


**Figure S7.7 –** Fluorescence emission spectra ($\lambda_{ex}$ = 295 nm) of LbOYE protein in the concentration of 1 µmol $L^{-1}$ in the presence of increasing concentrations of the **(A)** $HL1^{Ch}$ and **(B)** $HL3^{FPh}$. The spectra were obtained in Tris-HCl buffer, pH 8.0; 100 mmol $L^{-1}$ of NaCl and 2.5% of DMSO and 25 °C .

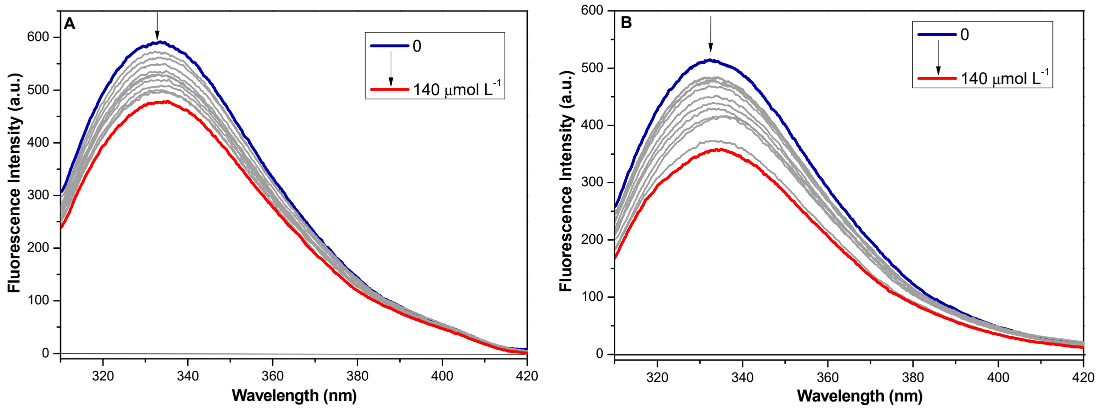

**Figure S7.8.** Fluorescence emission spectra ($\lambda_{ex}$ = 295 nm) of LbOYE protein in the concentration of 1 μmol $L^{-1}$ in the presence of increasing concentrations of the [Au(HL1$^{Ch}$)$_2$]Cl (**Au1**) complex. (B) Variation of maximum fluorescence intensities ($\lambda_{max}$ = 333 nm) and non-linear fit to the Hill equation (Equation 1). (C) Logarithmic relationship to obtain the number of bonding sites according to Equation 2. (D) Comparison between the temperatures of 25 °C and 37 °C. The spectra were obtained in Tris-HCl buffer, pH 8.0; 100 mmol $L^{-1}$ of NaCl and 2.5 % of DMSO.

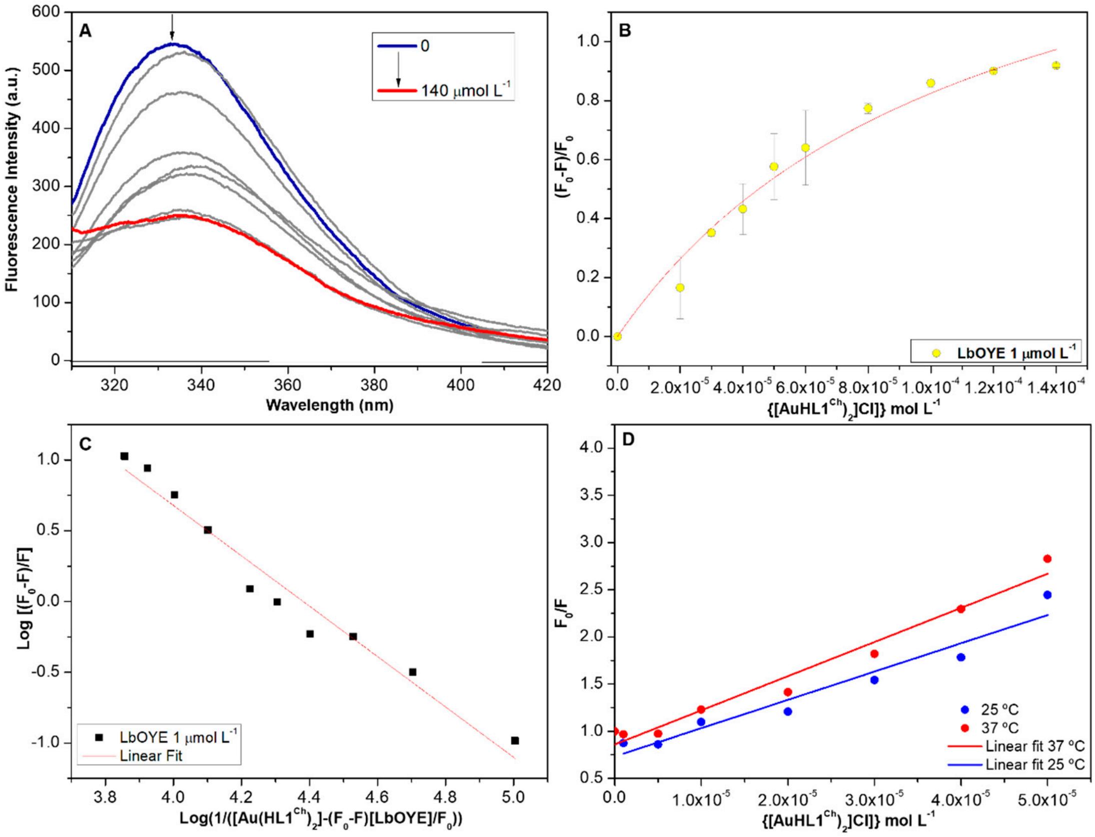

**Figure S8.1** – Representative figure of the HL1$^{Ch}$ ligand at the site of the OLD Yellow enzyme from *Leishmania braziliensis*.

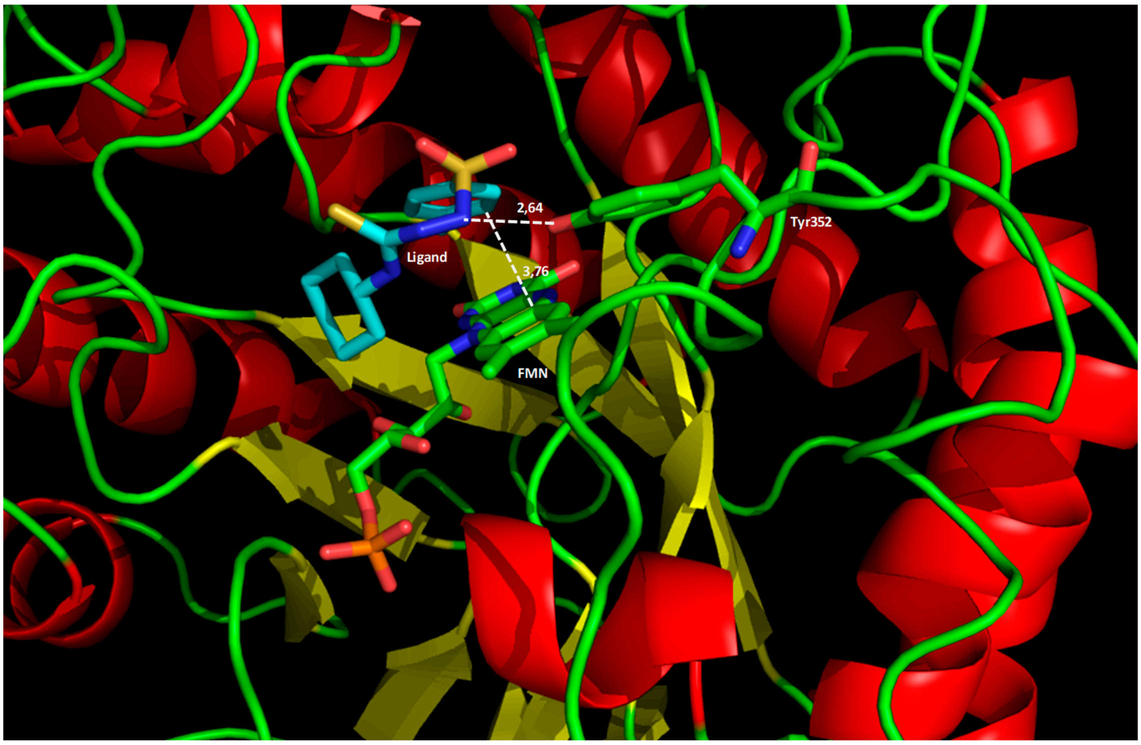


**Figure S8.2** – Representative figure of the HL3$^{FPh}$ ligand at the site of the OLD Yellow enzyme from *Leishmania braziliensis*.

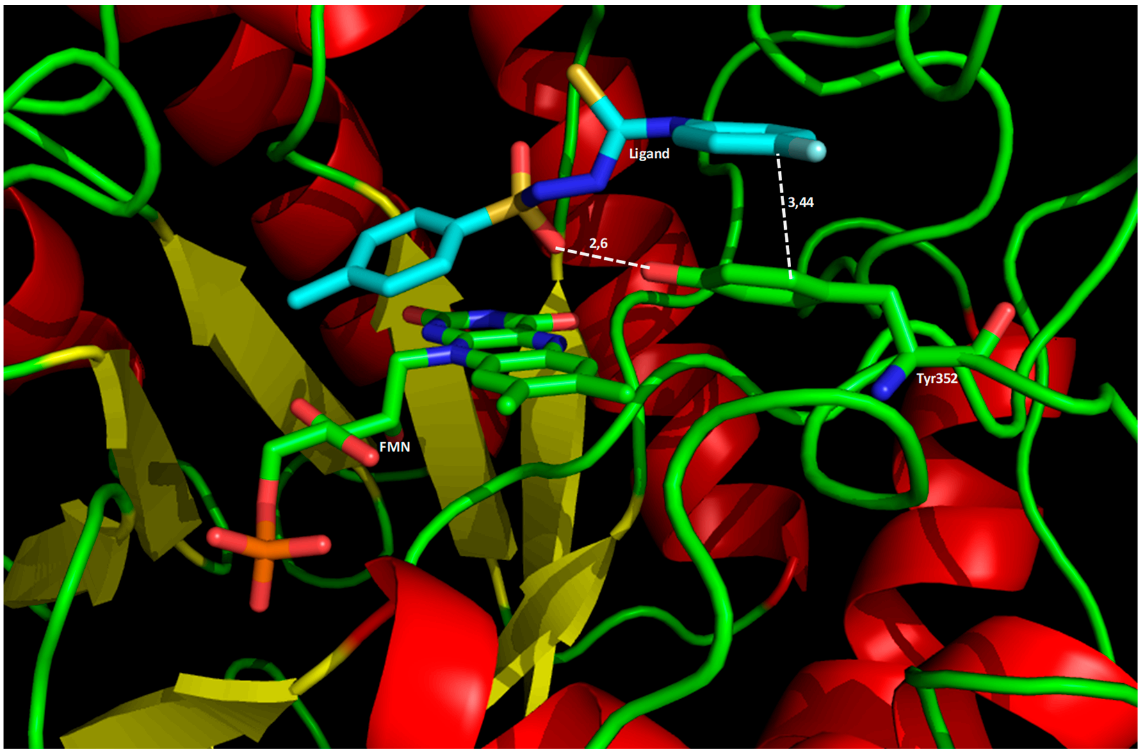

**Figure S8.34 –** Representative figure of the HL4$^{ClPh}$ ligand at the site of the OLD Yellow enzyme from *Leishmania braziliensis*.

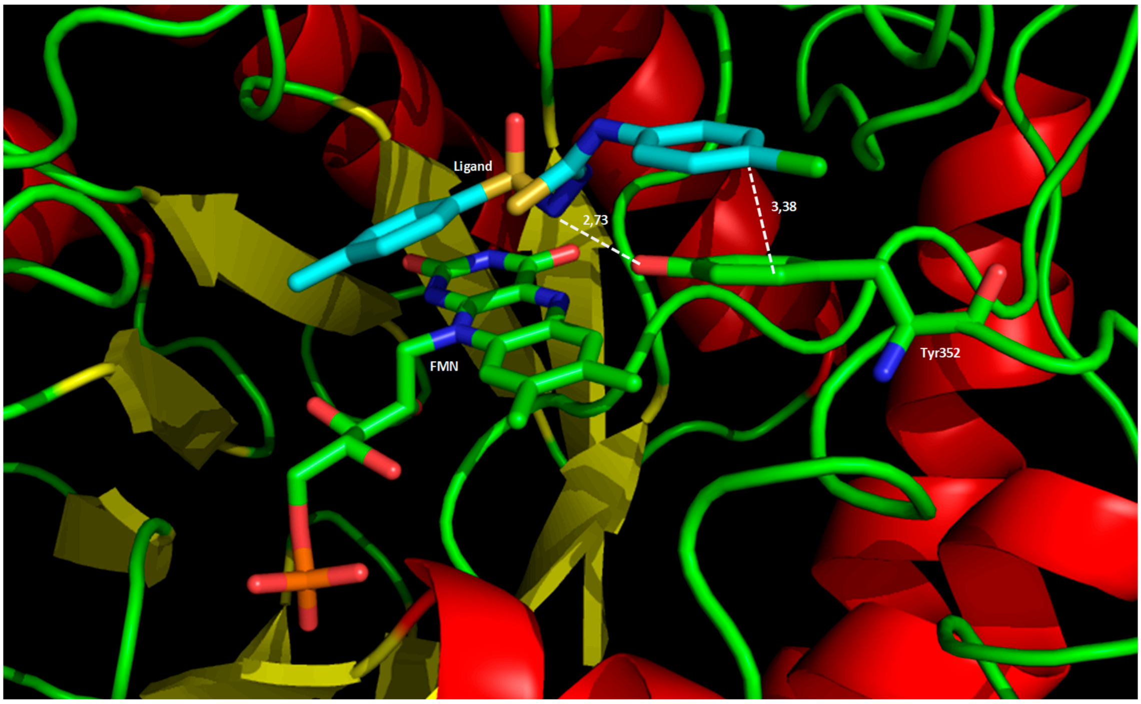


**Figure S8.35 –** Representative figure of the HL5$^{NO2Ph}$ ligand at the site of the OLD Yellow enzyme from *Leishmania braziliensis*.

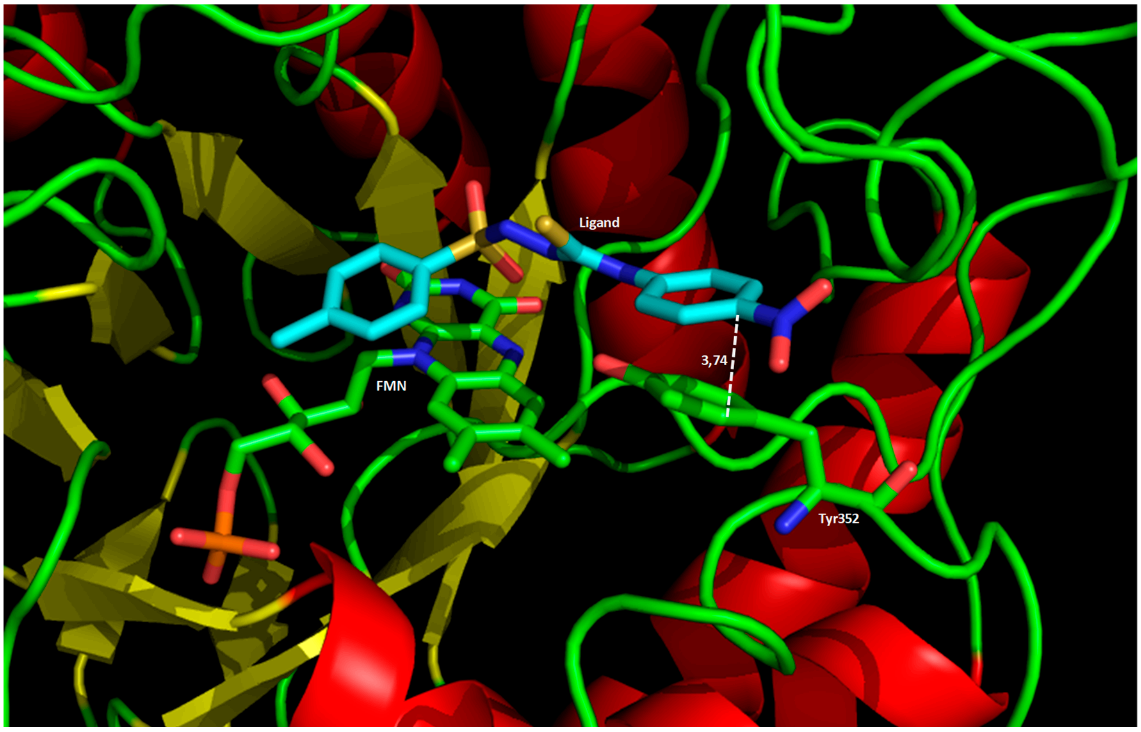

**Figure S8.36 – –** Representative figure of the HL6[Al] ligand at the site of the OLD Yellow enzyme from *Leishmania braziliensis*.

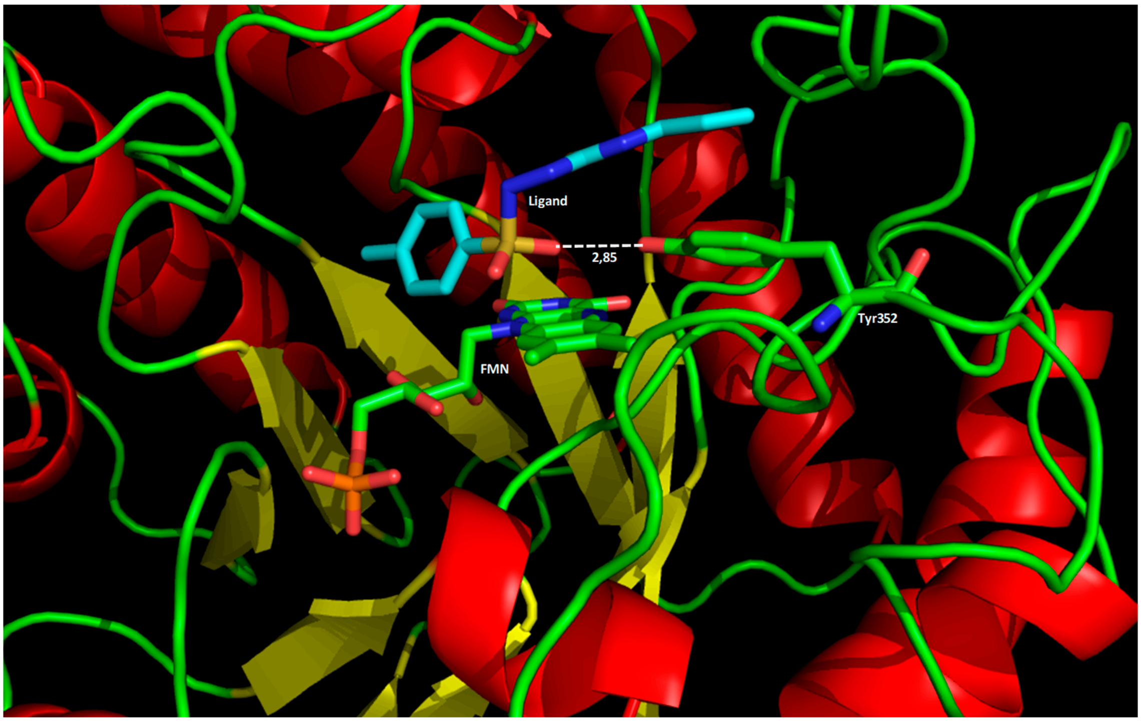


**Figure S8.37 –** Representative figure of the $[Ag(HL1^{Ch})_2]NO_3$ complex at the site of the OLD Yellow enzyme from *Leishmania braziliensis*.

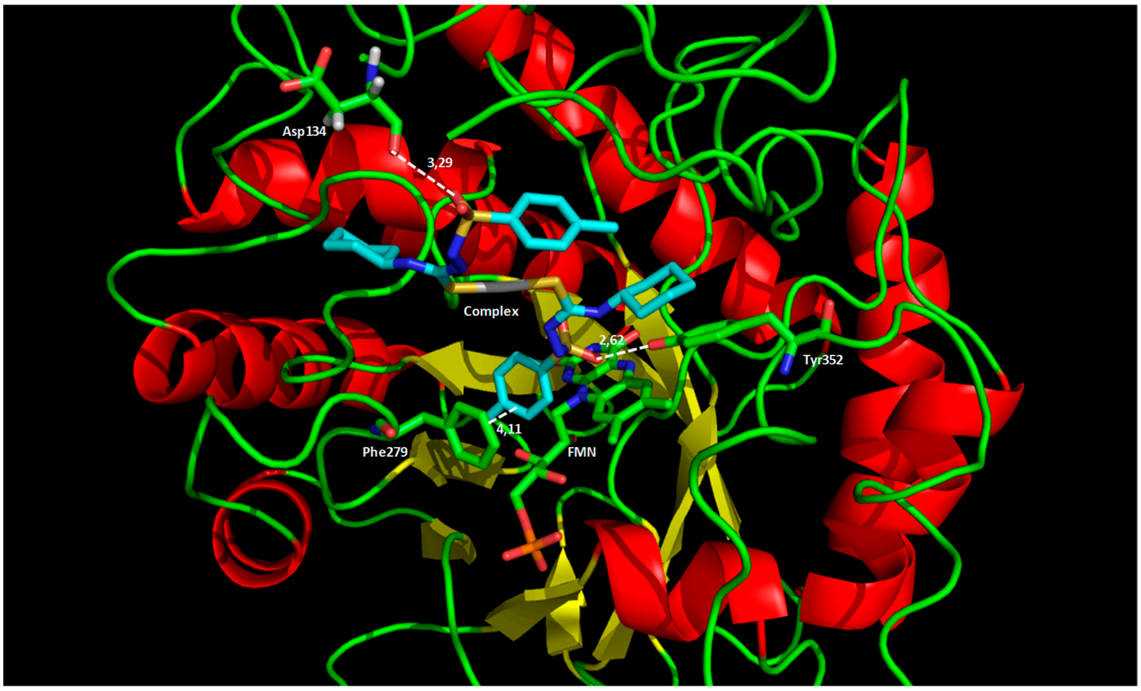

**Figure S8.38 –** Representative figure of the [Ag($HL2^{Ph}$)$_2$]$NO_3$ complex at the site of the OLD Yellow enzyme from *Leishmania braziliensis*.

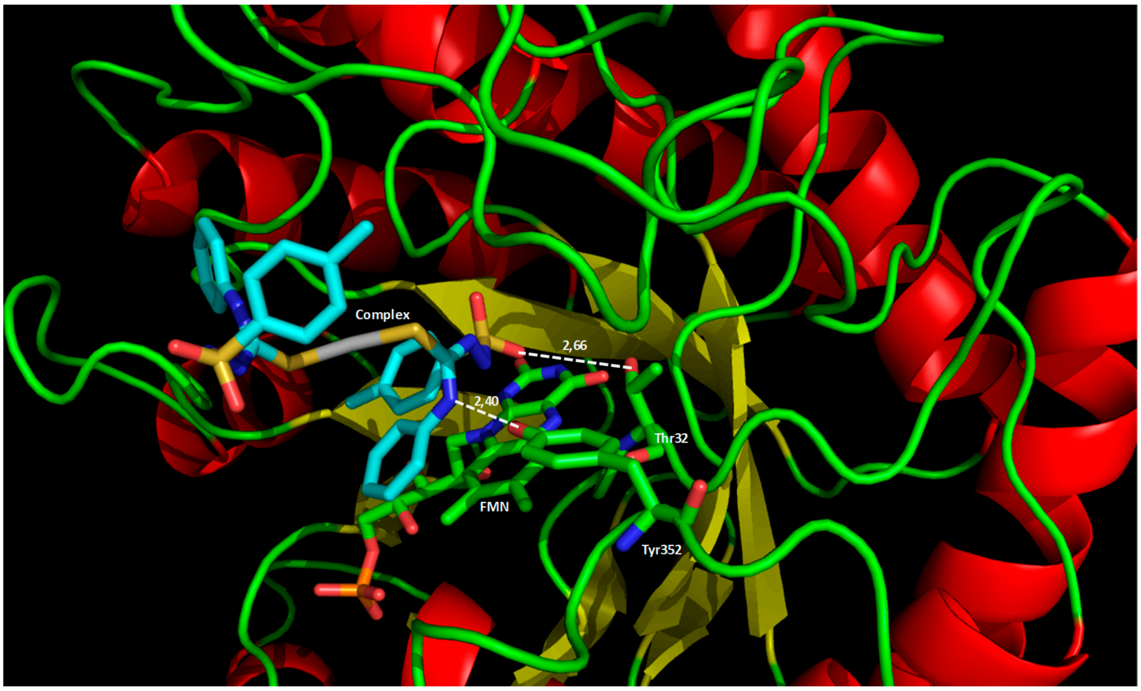


**Figure S8.39 –** Representative figure of the [Ag($HL3^{FPh}$)$_2$]$NO_3$ complex at the site of the OLD Yellow enzyme from *Leishmania braziliensis*.

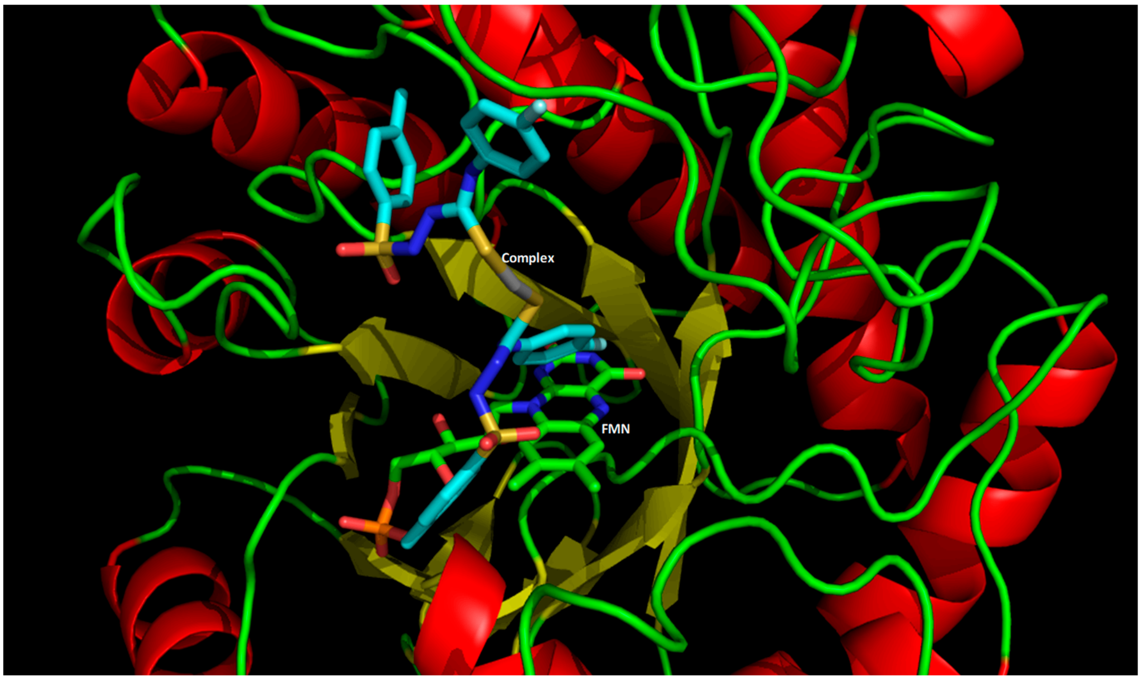

**Figure S8.40 –** Representative figure of the [Ag(HL4$^{ClPh}$)$_2$]$NO_3$ complex at the site of the OLD Yellow enzyme from *Leishmania braziliensis*.

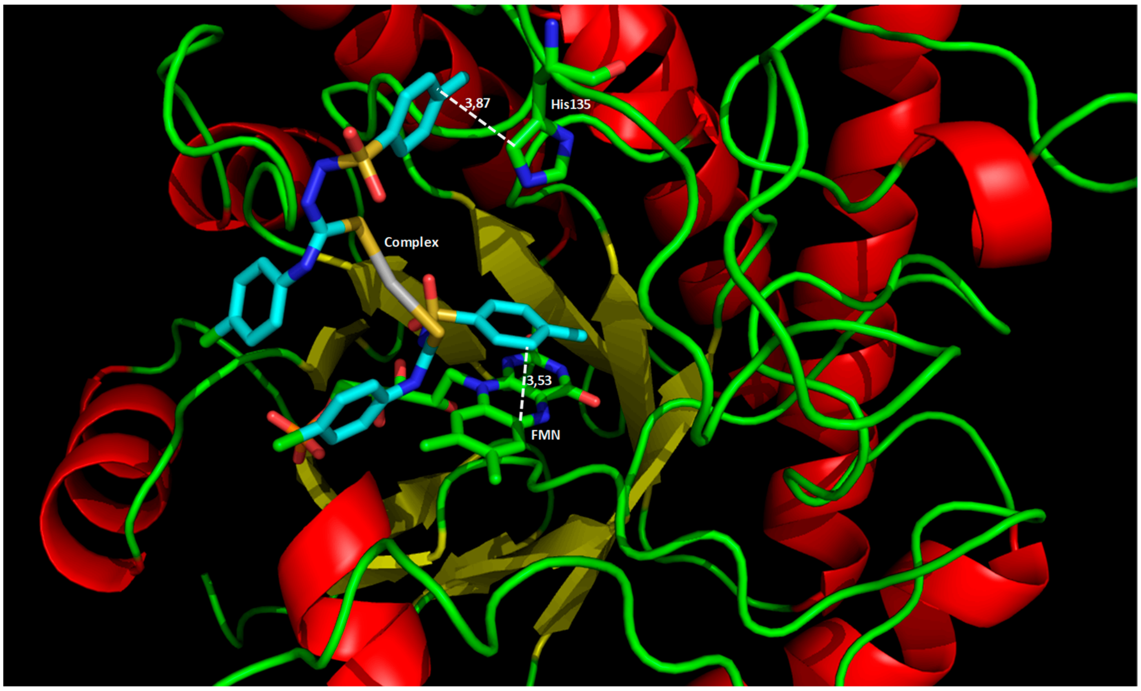


**Figure S8.41 –** Representative figure of the [Ag(HL5$^{NO2Ph}$)$_2$]$NO_3$ complex at the site of the OLD Yellow enzyme from *Leishmania braziliensis*.

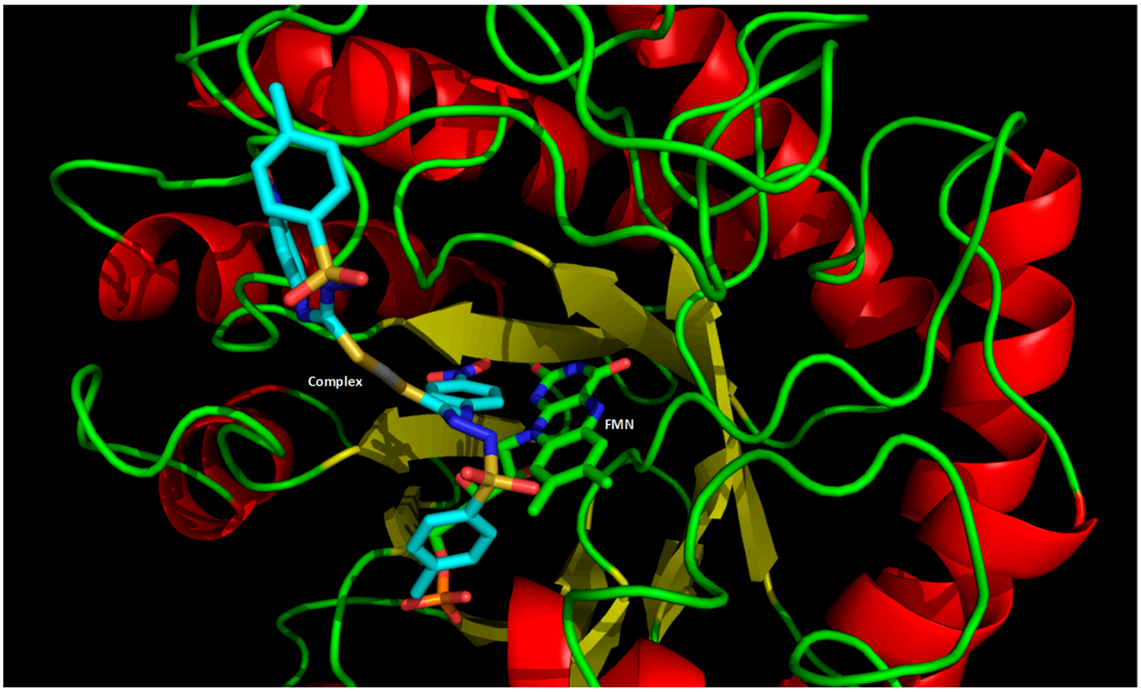

**Figure S8.42 –** Representative figure of the [Au(HL1$^{Ch}$)$_2$]Cl complex at the site of the OLD Yellow enzyme from *Leishmania braziliensis*.

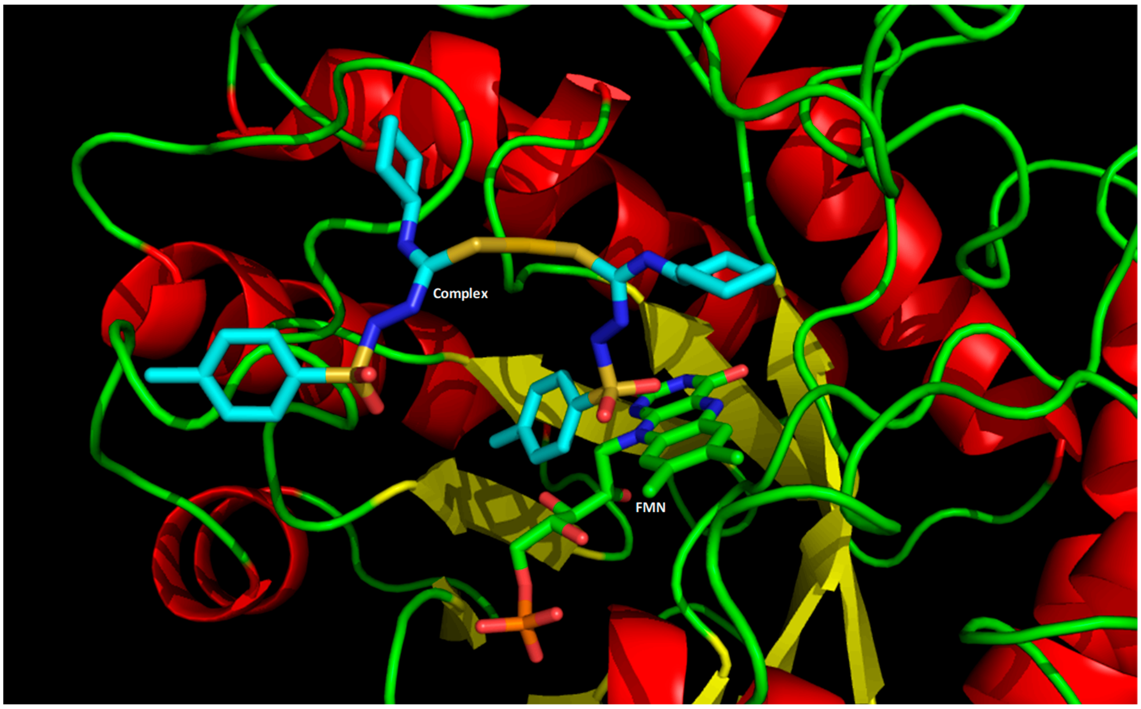


**Figure S8.43 –** Representative figure of the [Au(HL2$^{Ph}$)$_2$]Cl complex at the site of the OLD Yellow enzyme from *Leishmania braziliensis*.

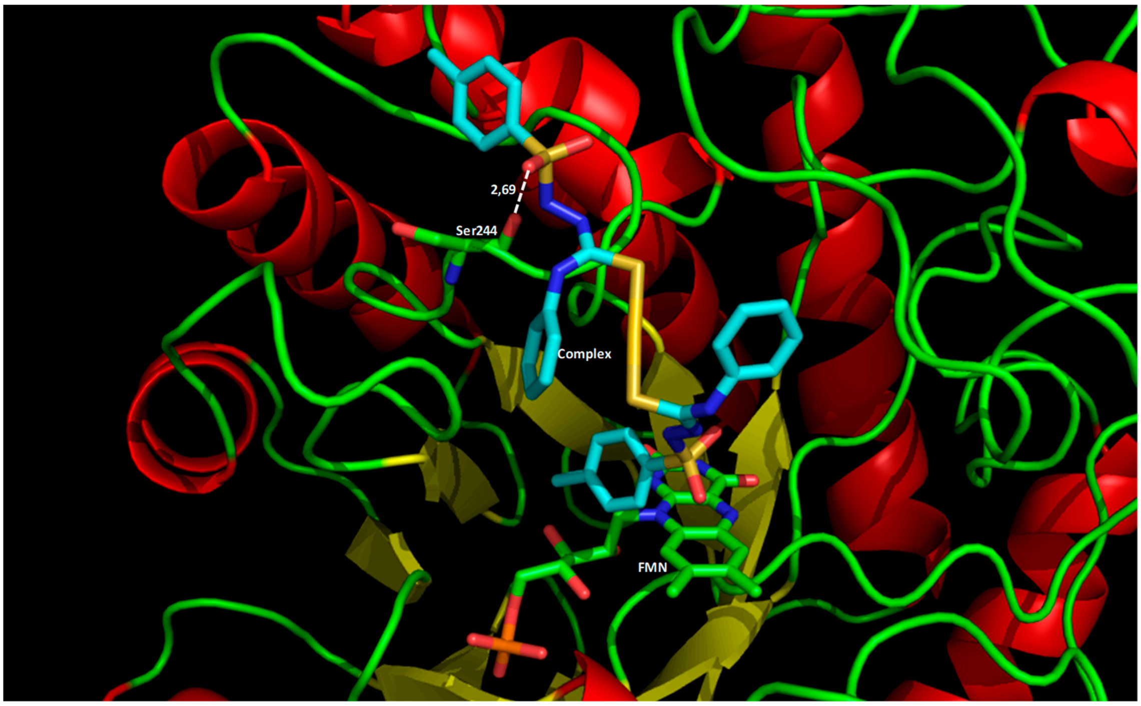

**Figure S8.44 –** Representative figure of the [Au(HL3$^{FPh}$)$_2$]Cl complex at the site of the OLD Yellow enzyme from *Leishmania braziliensis*.

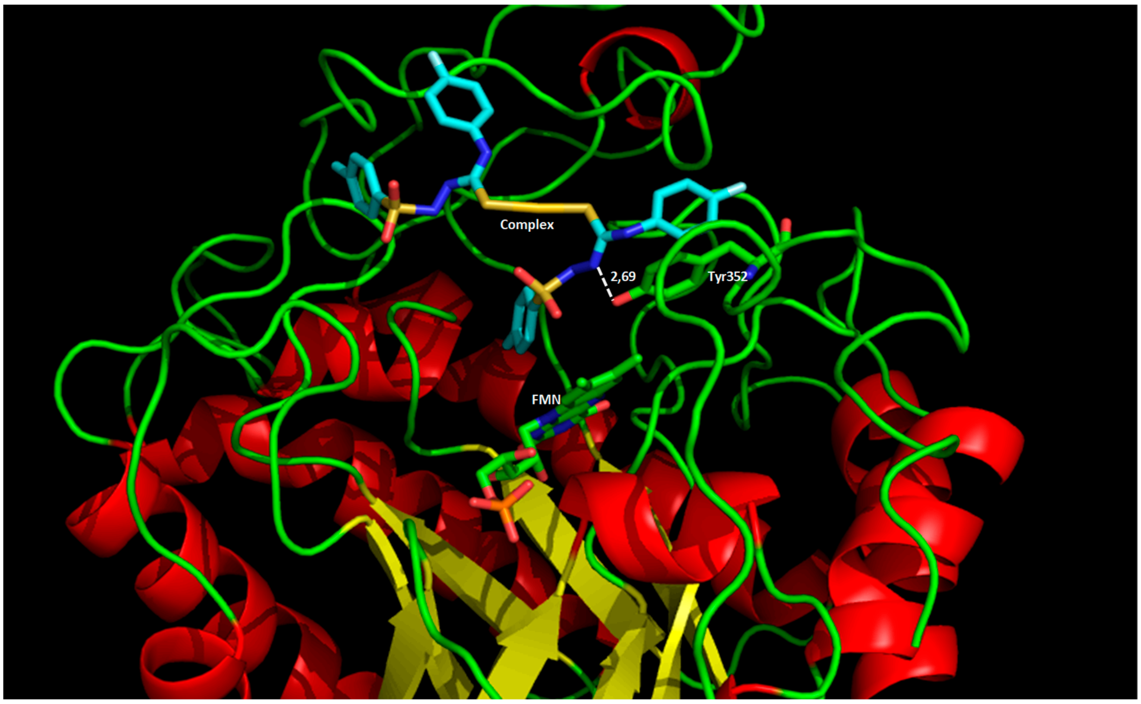


**Figure S8.45 –** Representative figure of the [Au(HL4$^{ClPh}$)$_2$]Cl complex at the site of the OLD Yellow enzyme from *Leishmania braziliensis*.

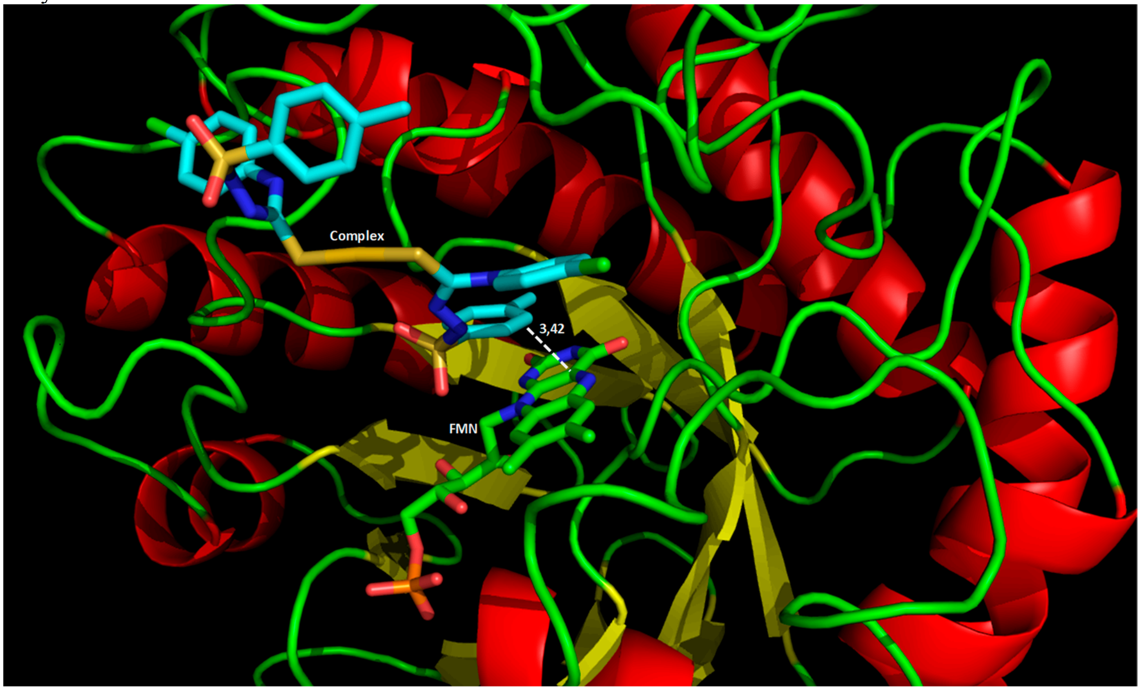

**Figure S8.46 –** Representative figure of the [Au(HL5$^{NO2Ph}$)2]Cl complex at the site of the OLD Yellow enzyme from *Leishmania braziliensis*.

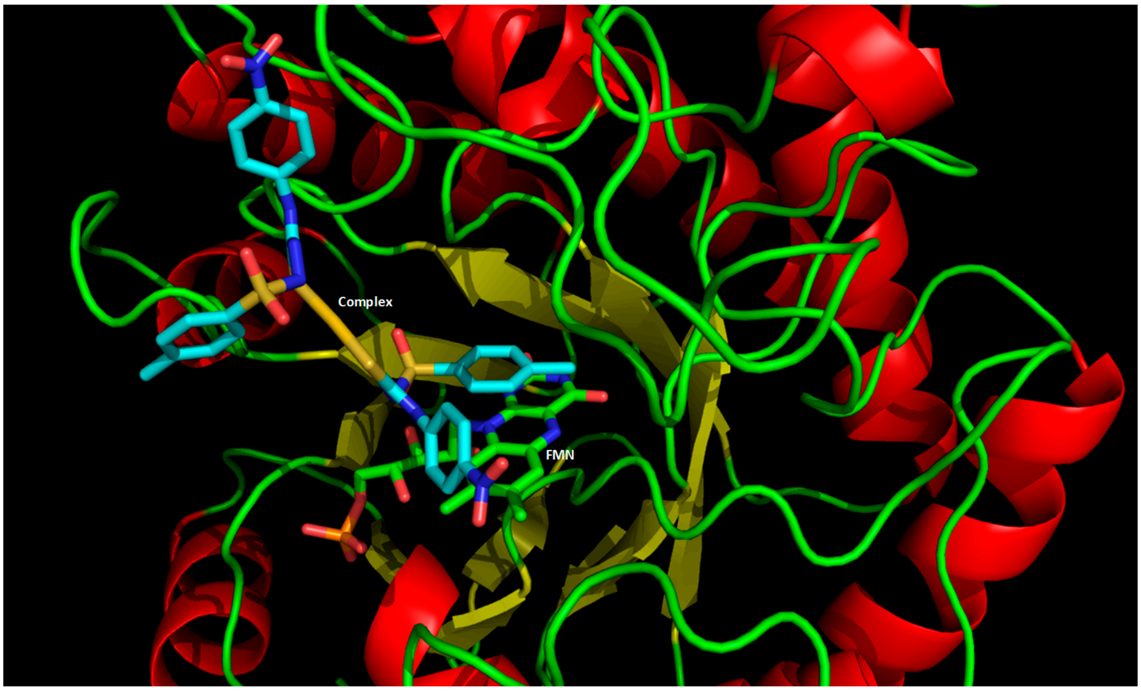

Supplement: Supplementary file 1 [file pharmaceutics-16-00452-s001.zip › pharmaceutics-2917125-supplementary.pdf]
